# A fruitful century for the scalable synthesis and reactions of biphenyl derivatives: applications and biological aspects

**DOI:** 10.1039/d3ra03531j

**Published:** 2023-06-16

**Authors:** Hajar A. Ali, Mohamed A. Ismail, Abd El-Aziz S. Fouda, Eslam A. Ghaith

**Affiliations:** a Chemistry Department, Faculty of Science, Mansoura University 35516 Mansoura Egypt Abdelghaffar@mans.edu.eg

## Abstract

This review provides recent developments in the current status and latest synthetic methodologies of biphenyl derivatives. Furthermore, this review investigates detailed discussions of several metalated chemical reactions related to biphenyl scaffolds such as Wurtz–Fittig, Ullmann, Bennett–Turner, Negishi, Kumada, Stille, Suzuki–Miyaura, Friedel–Crafts, cyanation, amination, and various electrophilic substitution reactions supported by their mechanistic pathways. Furthermore, the preconditions required for the existence of axial chirality in biaryl compounds are discussed. Furthermore, atropisomerism as a type of axial chirality in biphenyl molecules is discussed. Additionally, this review covers a wide range of biological and medicinal applications of the synthesized compounds involving patented approaches in the last decade corresponding to investigating the crucial role of the biphenyl structures in APIs.

## Abstract 2

This review provides recent developments in the current status and latest synthetic methodologies of biphenyl derivatives.

## Graphical abstract

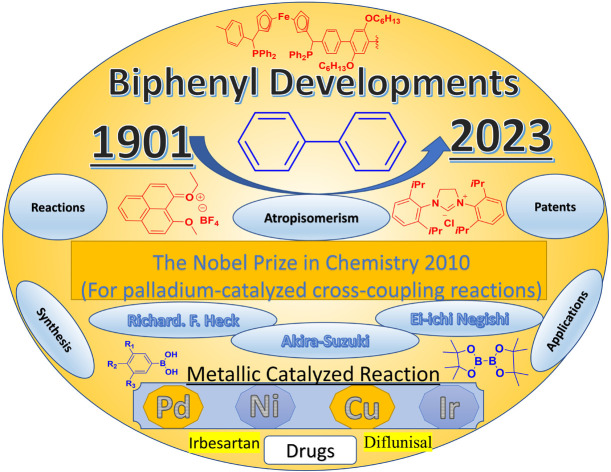

## 1. Introduction and scope

**Fig. 1:**
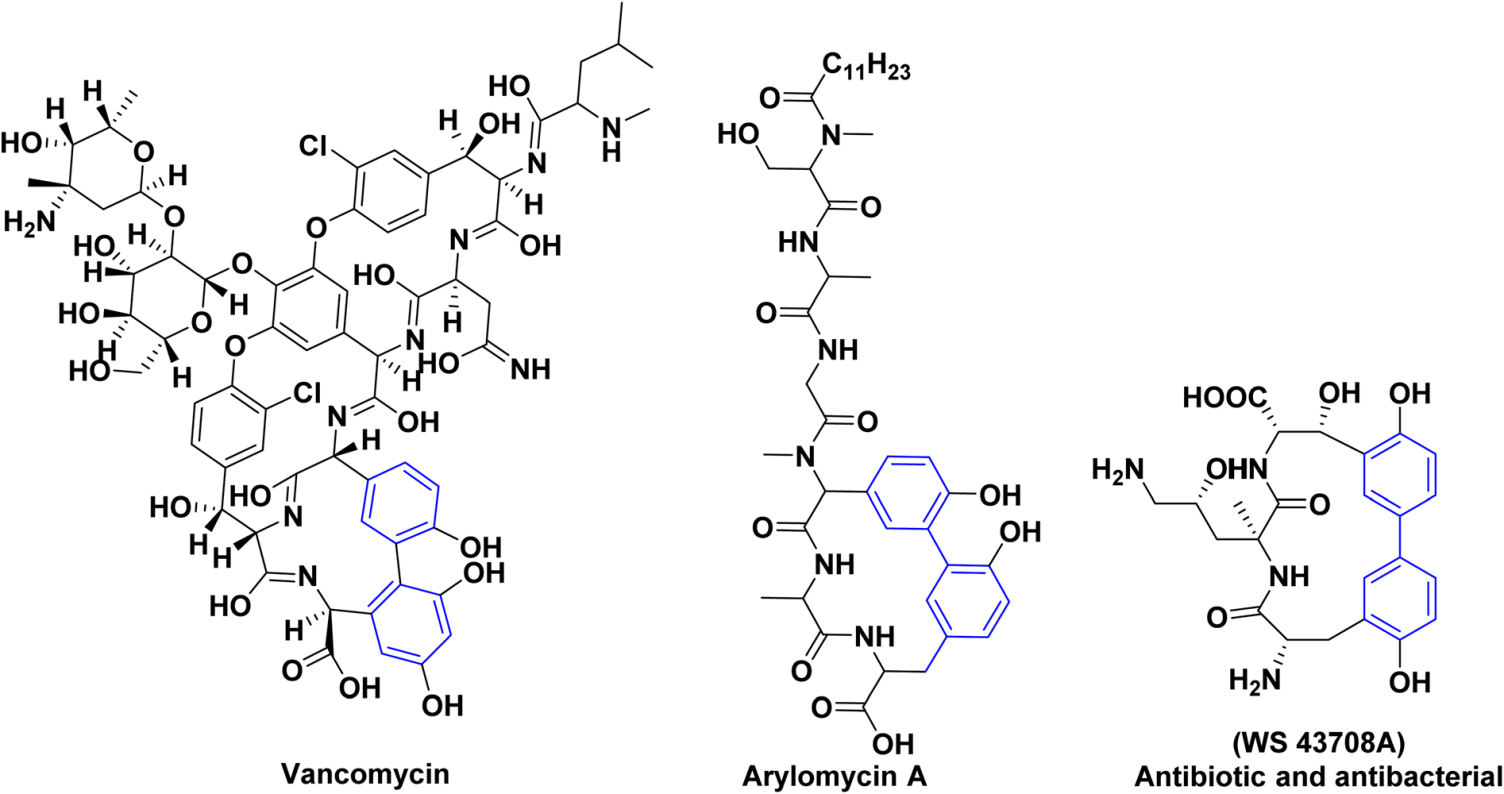
Exemplary skeletons for natural products containing biphenyl moieties.

For many decades, biphenyl compounds and their isosteres have been considered a fundamental backbone in synthetic organic chemistry and natural products due to their omnipresence in medicinally active compounds, marketed drugs and natural products. For example, there are various biologically active natural products that contain biaryl scaffolds (vancomycin,^1,2^ WS 43708A^3^ and Arylomycin A^4^) (Fig. 1).

**Fig. 2:**
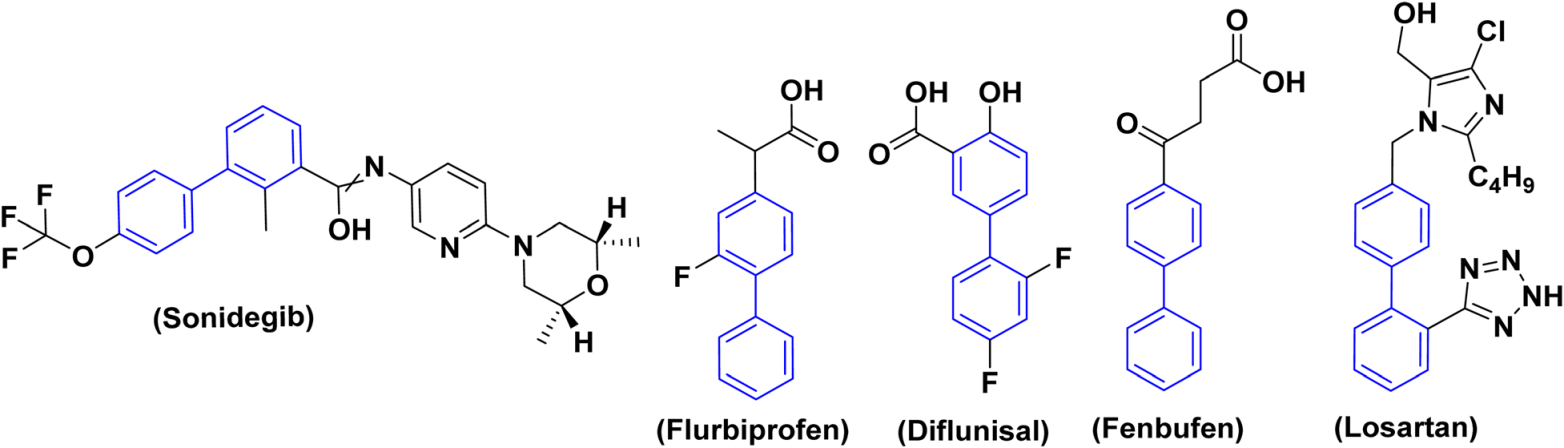
Representative examples for biphenyl containing marketed drugs.

Since biphenyls are neutral molecules without a functional group, functionalization is required for them to react. Biphenyls consist of two benzene rings linked at the [1,1′] position. The reactions of biphenyls are similar to benzene as they both undergo electrophilic substitution reaction. Biphenyl derivatives which are used to produce an extensive range of drugs, products for agriculture, fluorescent layers in organic light-emitting diodes (OLEDs)^5,6^ and building blocks for basic liquid crystals are significant intermediates in organic chemistry besides being the structural moiety of an extensive range of compounds with pharmacological activities.^7–10^ Whereas several biphenyl derivatives serve as versatile and multifaceted platforms in medicinal chemistry, as large number of biphenyl derivatives are patented and broadly used in medicine as the antiandrogenic,^11^ immunosuppressant, antifungal, antibacterial, antimicrobial, anti-inflammatory, anti-proliferative, osteoporosis, antihypertensive, antitumor, β-glucuronidase inhibition activity, anti-leukemia agent hypotensive, anti-cholinesterase, anti-diabetic and antimalaria drugs.^12^ In addition, 6-(3-(adamantan-1-yl)-4-methoxyphenyl)-2-naphthoic acid which trademark drug (adapalene) as a third-generation topical retinoid primarily used for treating acne vulgaris, anti-inflammatory, antibacterial.^13^ Another example is sonidegib which acts as a drug for basal cell carcinoma.^14^ Additionally, other manufactured naturally occurring chemicals, including the biphenyl nucleus, have shown noteworthy biological activities, such as the antipyretic properties of fenbufen^15^ and flurbiprofen and their role as non-steroidal anti-inflammatory drugs (NSAIDs) (Fig. 2).^16^

## 2. Synthesis of biphenyl systems

Wurtz reported the first trial for carbon–carbon bond formation reactions between two alkyl halides in the presence of sodium metal. Then, Fitting expanded this work to include the C(sp^2^)–C(sp^2^) homodimerization of aryl halides. Whereby, Ullmann described the Cu-catalyzed homocoupling reaction involving halo-arenes in 1901. In 1914, Bennett and Turner demonstrated the homodimerization of Grignard reagent and phenyl magnesium bromide which promoted by chromium(iii) chloride (CrCl_3_) or anhydrous cupric chloride (CuCl_2_).^17^ Kumada depicted the reaction of aryl halides with arylmagnesium halide or organozinc in the presence of transition metal catalysts.^18–20^ Whereas, Hiyama produced biaryls *via* coupling the readily available organosilanes with organohalides.^21,22^ In the same context, Negishi *et al.*^23^ reported a regio- and chemoselective method for biaryl synthesis.^24,25^ In 1986, a new methodology for cross coupling reaction of organic electrophiles with organostannanes was discovered by Stille.^26,27^ After that, Suzuki–Miyaura cross coupling became one of the most effective and widely used ways for forming carbon–carbon bonds.^15,28,29^ As biphenyl scaffolds are a key step to produce bioactive molecules used for medicine. In addition, biphenyl compounds were isolated by distillation from natural sources such as crude oil, natural gas, and coal tar.^6^ Several methods are available in the literature for the synthesis of substituted biphenyl scaffolds *through* using various chemical building blocks according to the following reaction routes:

### 2.1 Wurtz–Fittig reaction

**Scheme 1:**
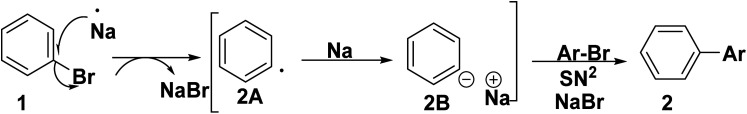
Free radical mechanism for Wurtz–Fittig reaction.

The history of the chemistry of biphenyl derivatives can be traced back almost 160 years ago. As the synthesis trials of biphenyl scaffolds started from 1855 till the end of the 19th century by many researchers as in 1855. Wurtz reported the first trial for C(sp^3^)–C(sp^3^) bond formation *through* using the sodium-mediate coupling reaction of two alkyl halides.^17^ In 1862, the reaction was extended by Fittig to include coupling between aryl anion and an electrophile such as aryl halide in the presence of various metal surface such as Na, Li, Au, Cu and Ag.^30^ Wurtz–Fittig reaction could be achieved *via* the free radical mechanism^31^ in three steps; dehalogenation, followed by diffusion of the dehalogenated molecules, and finally, coupling process between two aryl halides 1 to afford biphenyl derivative 2.^32^ It is worthy to mention that, bulky substitution group leads to kinetically prevent Wurtz–Fittig coupling as one of its drawbacks.^33^ Also, Wurtz–Fittig reaction plays an important role in the synthesis of thermoplastics polymer synthesis (polyphenylenes and oligophenylenes) (Scheme 1).^34^

### 2.2 Ullmann reaction

**Scheme 2:**
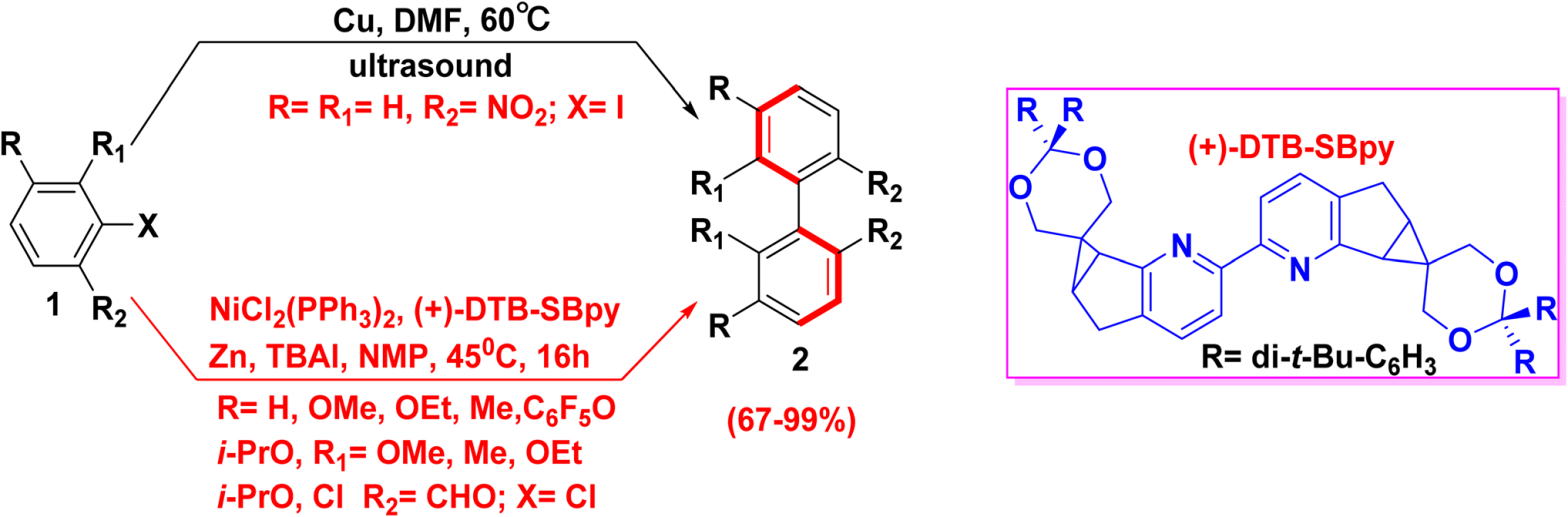
General Ullman cross-coupling reaction for the synthesis of biphenyl derivatives.

The Ullmann reaction was known since 1901,^35^ as it has attracted the attention of many chemists trying to synthesize chiral substituted biphenyl. Moreover, the Ullmann reaction usually is achieved at lower temperatures and shorter time frame *through* coupling the aryl halide 1 in the presence of powdered copper or nickel catalysts, but it has changed in recent years.^36,37^ It is worth mentioning that, the degree of stability of substituted chiral biphenyl depends on the nature of the *ortho* substituents when this asymmetric Ullman coupling.^38^ Whereas, enantioselective biphenyls 2 were obtained *via* the reaction of *ortho*-chlorinated benzaldehyde derivatives 1 in the presence of Ni catalyst (Scheme 2).^39^

### 2.3 Bennett–Turner

**Scheme 3:**
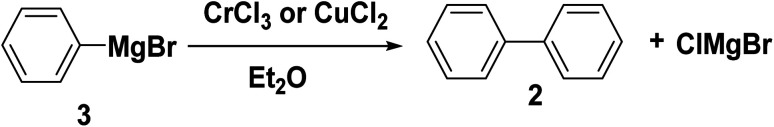
Preparation of biphenyls *via* Bennett–Turner.

In 1914, the coupling reaction was investigated by Bennett–Turner^40^ who reported the synthesis of biphenyl compound 2*via* homocoupling reaction of phenylmagnesium bromide (3) and CrCl_3_ in diethyl ether (Et_2_O), incidental to an attempt to prepare organochromium compound.^41^ Bennett–Turner reaction can be achieved by using CuCl_2_.^42^ In this context, noteworthy is the contribution of Krizewsky with Turner using a CuCl_2_ promoted homocoupling reaction instead of CrCl_3_ (Scheme 3).^17,42,43^

**Scheme 4:**
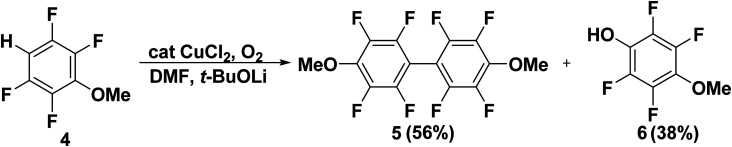
Copper catalyzed reaction of aryl lithium with oxygen.

Ultimately, treatment of the tetrafluoro-3-methoxybenzene (4) with CuCl_2_ and lithium *tert*-butoxide (*t*-BuOLi) in the presence of O_2_ gas produced a mixture of biphenyl coupling product 5 (56%) and the corresponding phenol 6 (38%). The proposed mechanism involves *in situ* deprotonation with oxidative dimerization has been studied by Do *et al.*^44^ The production of corresponding phenol was reasonable *through* trapping of the formed aryl lithium with oxygen gave rise to an aryl anion species which was less prone to oxygenation and more likely to form a copper adduct that would undergo oxidative coupling reaction (Scheme 4).

**Scheme 5:**
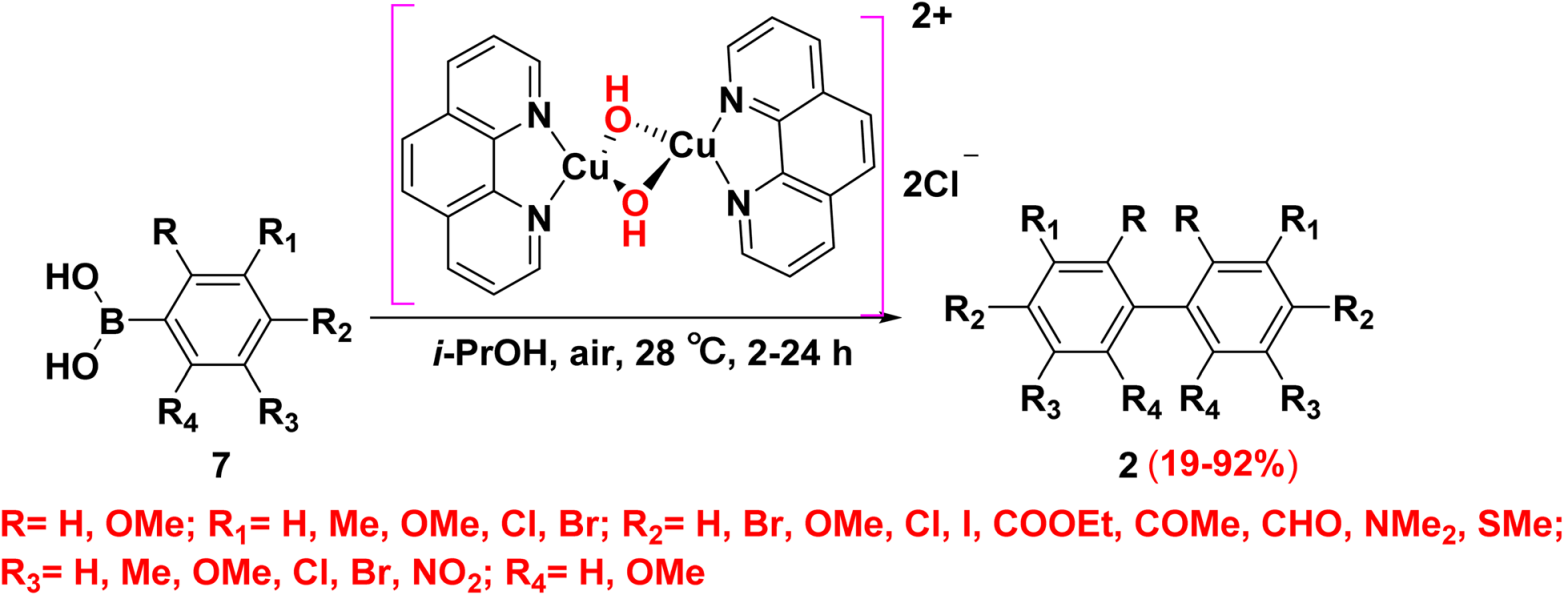
A reasonable mechanism for the Cu-catalyzed homocoupling of aryl boronic acids.

In 2003, Demir and coworkers reported using of low-cost copper salts (Cu(i) and Cu(ii)) as they are able to mediate the dimerization of arylboronic acids 7 to afford the corresponding symmetrical substituted biaryl 2 in good yields. The reaction can be possibly catalyzed under an oxygen atmosphere without a significant loss of yields.^45^ In 2009, Kirai *et al.*^46^ improved the previous protocol for oxidative dimerization which relying on catalysts that would facilitate transmetalation. As they reported a new effective methodology for homocoupling of aryl boronic acid derivatives 7 under stirring conditions *through* using the catalytic amount of [{(phen)Cu(μ-OH)}_2_Cl_2_]·3H_2_O. It is worthy to mention that the homocoupling reaction proceeds without any additives such as oxidant or base. Besides, this method tolerates different substituents on the arylboronic acids such as nitro group, halogens and carbonyls (Scheme 5).

**Scheme 6:**
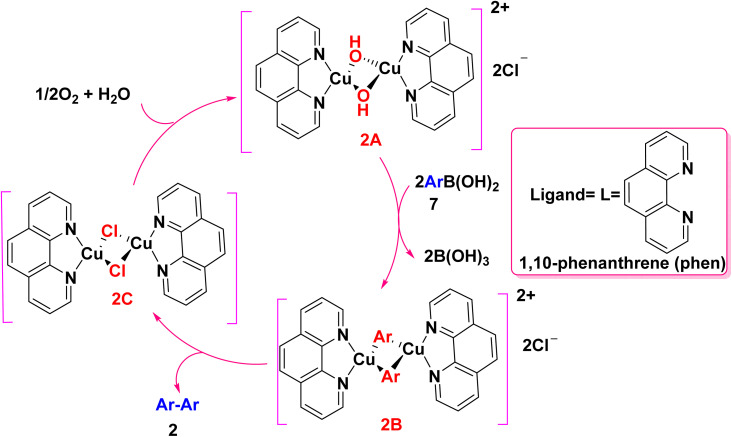
Possible mechanism for copper catalyzed homocoupling reaction.

The formation of symmetrical substituted biaryl 2 is explained according to the following postulated mechanism: the reaction proceeds by the hydroxido ligand attacks the oxophilic boron center, followed by transmetalation of arylboronic acids 7 with (μ-hydroxido)copper(ii) complex 2A to yield bimetallic arylcopper(ii) intermediate 2B without using any base. Biaryl products 2 afford *through* the concomitant one-electron reduction of each copper center [2LCu^II^–Ar → 2LCu^I^ + Ar–Ar]. As the binuclear copper models efficiently activate molecular oxygen, in the same time the molecular oxygen smoothly bind to the resulting bimetallic intermediate [{(phen)Cu^I^(μ-Cl)}_2_] 2C, which is reoxidized to afford (μ-hydroxido)copper(ii) complex 2A (Scheme 6). The present binuclear O_2_ activation and binuclear reductive elimination mechanism is quite different from that proposed for palladium-catalyzed aerobic homocoupling.^46^

### 2.4 Kumada cross coupling

**Scheme 7:**
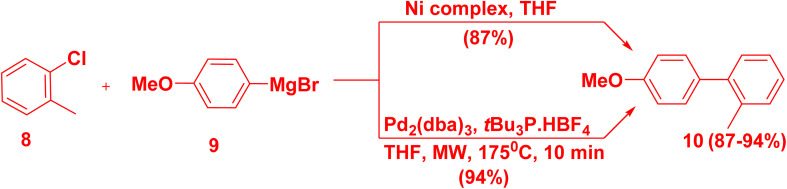
Kumada cross-coupling reaction for biaryl synthesis.

Since 1972, Kumada catalyzed cross coupling reaction of organozinc or organomagnesium with arylhalide in the presence of Pd, Ni, Cu, Fe or Co catalyst.^18–20^ However, this reaction was limited with Pd catalyst compared to Ni catalyst due to the high reactivity of organomagnesium,^47^ as well as less toxicity and non-expensive Ni catalyst.^48^ Besides, arylchlorides were preferable than aryliodides and arylbromides because of low price. Treatment of chloromethylbenzene 8 with (methoxyphenyl)magnesium bromide 9 in the presence of Ni complex and THF afforded 4′-methoxy-2-methyl-biphenyl 10 in (87%) yield.^49^ Whereas, in the presence of Pd catalyst, product 10 produced in (94%) yield (Scheme 7).^50^

**Scheme 8:**
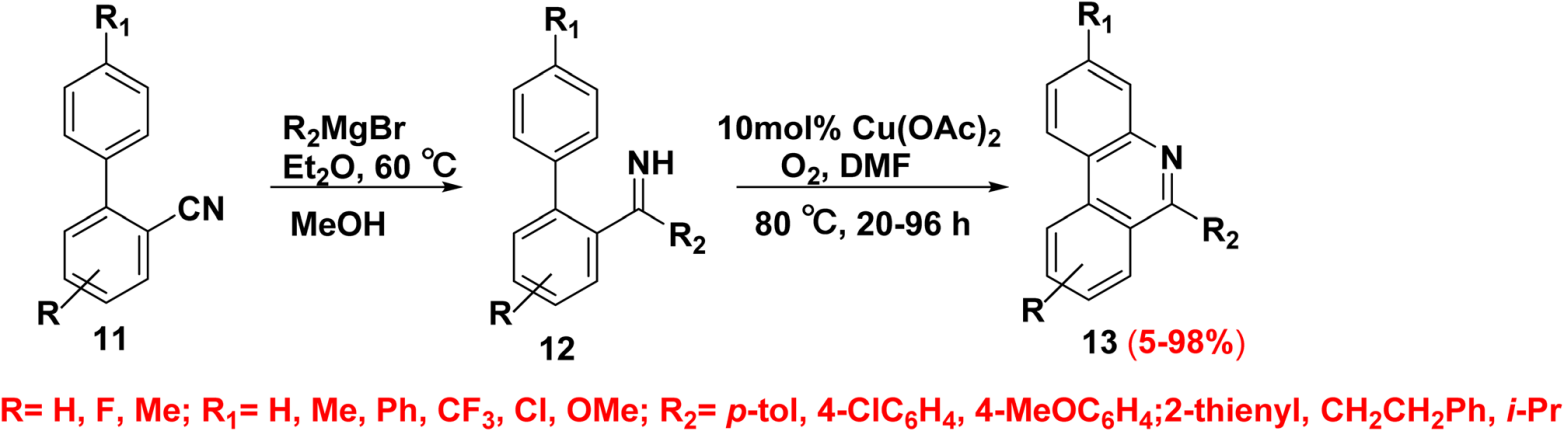
Copper catalyzed oxidation phenanthridine.

Recent research has largely been focused on directed C–H activation. Primary imines 12 produced by a Grignard reagent's nucleophilic addition to nitrile group 11 which is susceptible to attack make it easier to introduce copper(ii) into a close C–H bond. Primary imine derivatives 12 underwent a closure under oxygen-mediated elimination affording the respective phenanthridine derivatives 13 (Scheme 8).^51,52^

**Scheme 9:**
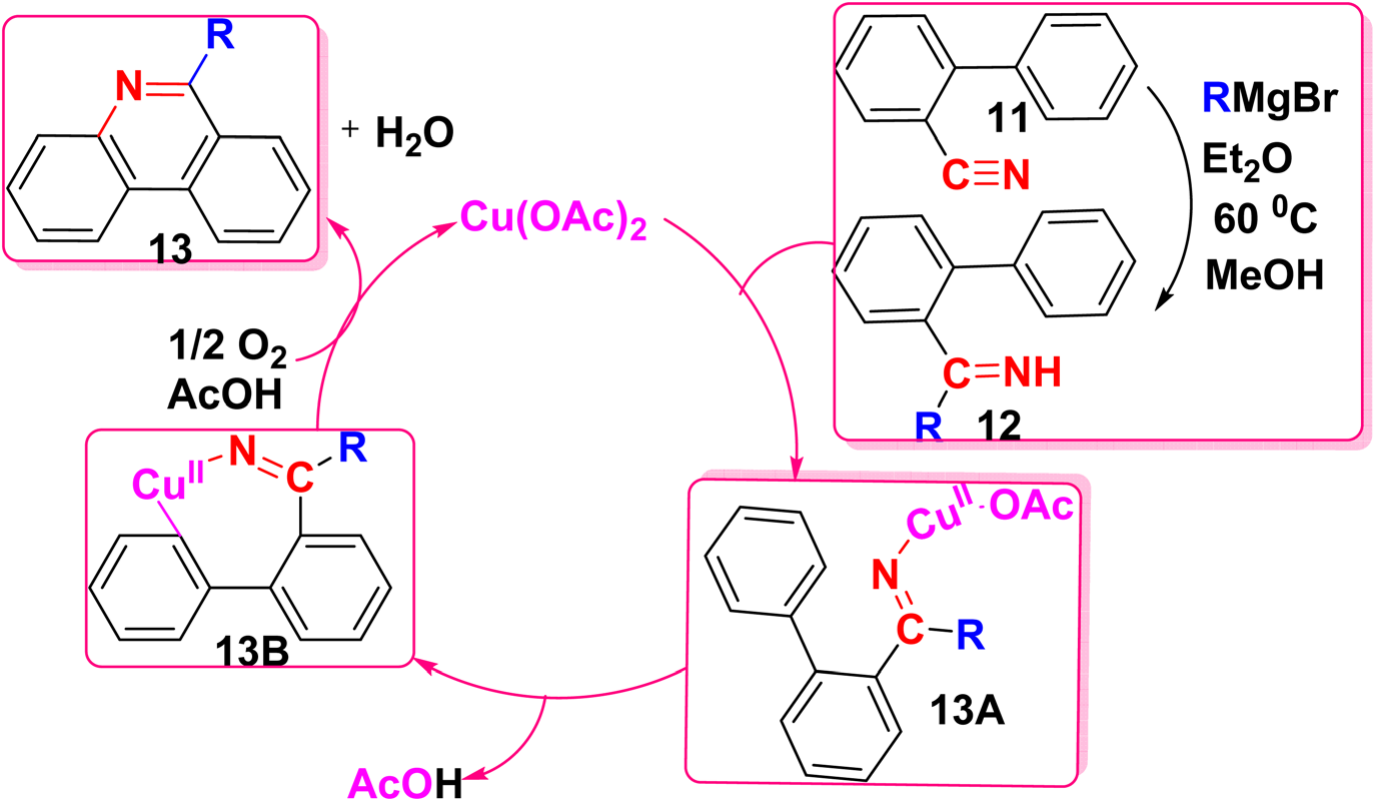
Mechanism for the oxidative formation of phenanthridine.

The proposed mechanism for the synthesis of the phenanthridine derivatives 13 is illustrated in Scheme 9. This involves the nucleophilic addition of biaryl-2-carbonitriles 11 and Grignard reagents to produce N–H imines 12, followed by their Cu-catalyzed C–N bond led to formation of the aromatic C–H bond, in which molecular oxygen is a necessary component to complete the catalytic process (Scheme 9).^51,52^

### 2.5 Hiyama cross-coupling

**Scheme 10:**
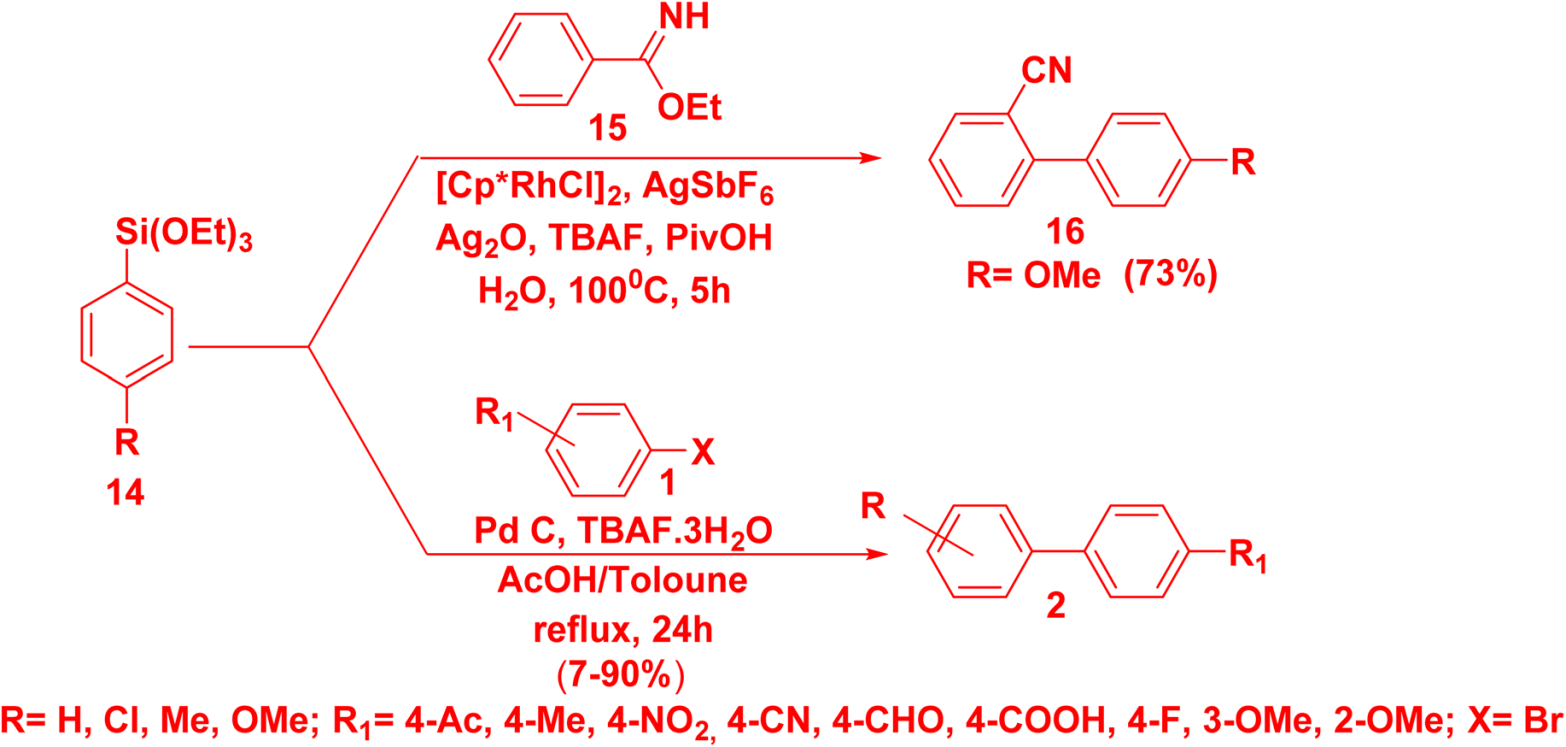
Synthesis of biphenyls 2*via* Hiyama cross-coupling.

One of the most important corn stone for synthesizing biaryl is Hiyama cross-coupling due to the distinctive features of organosilane reagent such as less reactivity, availability, non-toxicity, high sustainability and cheap, in addition to its ability to functionalize stereo and regioselective compounds.^53–55^ Rhodium-catalyzed *o*-C–H arylation reaction of arylsilane 14 with ethyl benzimidate 15 with in the presence of pentamethylcyclopentadienyl rhodium dichloride dimer [Cp*RhCl_2_]_2_, silver hexafluoroantimonate(v) (AgSbF_6_), silver oxide (Ag_2_O) and TBAF produced biphenyl-2-carbonitrile derivative 16.^56^ Whereby, refluxing a mixture of arylsilane 14 with arylhalide 1 in a mixture of AcOH and toluene, Pd catalyst, and tetrabutylammonium fluoride (TBAF) as a base afforded biphenyl 2. Furthermore, this method was applied under mild condition and tolerated high functional groups (Scheme 10).^57–59^

**Scheme 11:**
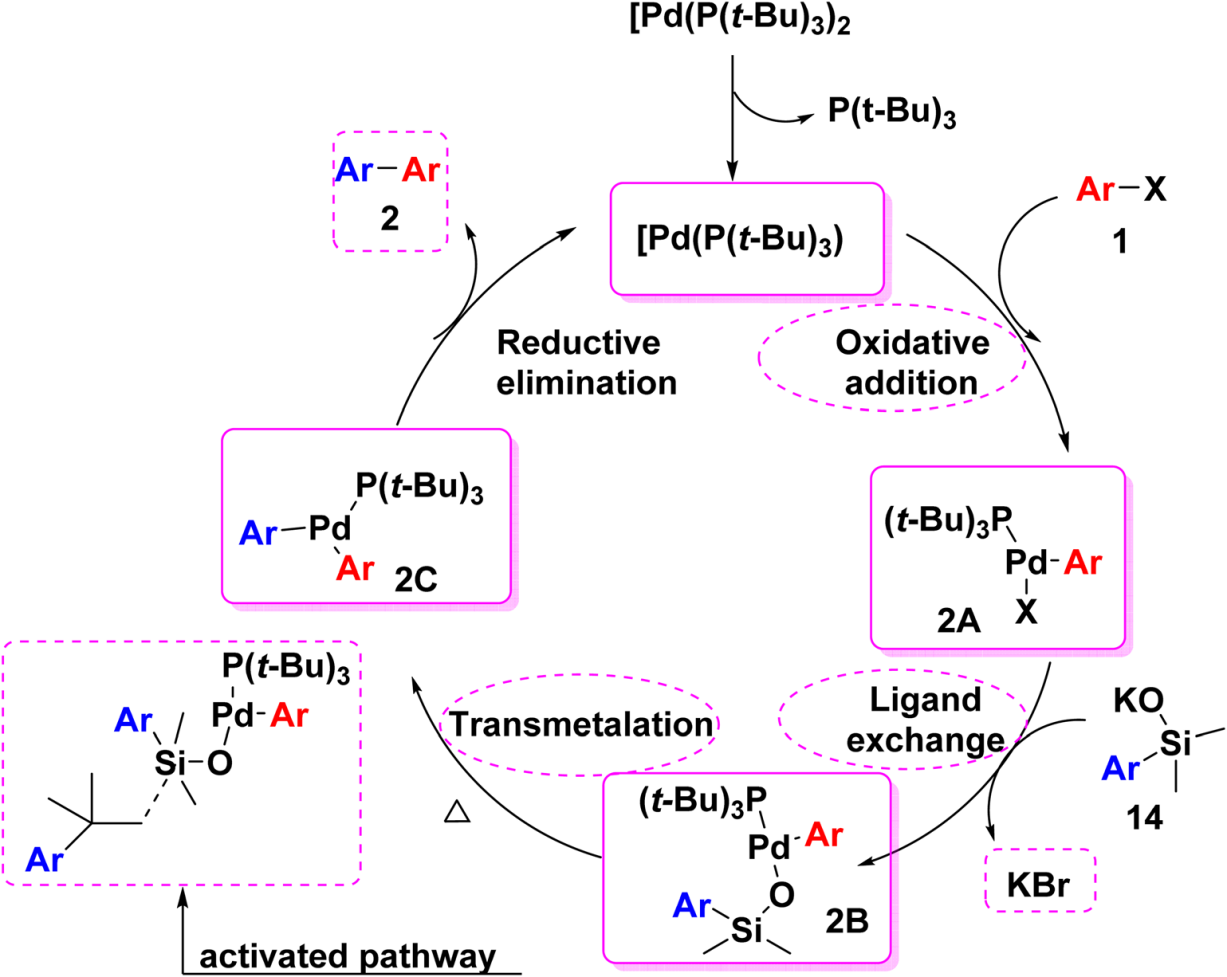
The postulated mechanism for Hiyama cross-coupling reaction.

Noor and coworkers^57^ suggested the proposed mechanism for Hiyama cross coupling. Analogous to other metal catalyzed cross coupling mechanism, Hiyama cross coupling passed through three steps. The oxidative addition of aryl halide to palladium metal leaded to the conversion of Pd(0) to Pd(ii). Then, transmetallation step occurred in the presence of base such as (KF, *n*-Bu_4_NF) which encouraged the C–Si bond to split and establish a new C–Pd bond.^53,60^ Finally, in the reductive elimination step a new C–C bond was formed and Pd returned back to the oxidation state (0) (Scheme 11).^24^

### 2.6 Suzuki–Miyaura cross-coupling

**Scheme 12:**
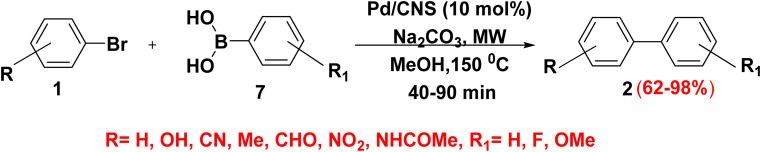
Suzuki coupling reaction toward synthesis of biphenyl derivatives.

In the last decade, Suzuki–Miyaura coupling reaction (SMC) has witnessed an increase interest due to its widespread academic and industrial application in the production of fine chemicals, polymers, materials, and pharmaceuticals.^61^ SMC reaction is also used to tag DNA with luminous intercalator substituents for DNA sequencing and diagnostics.^62^ In 1979, Akira Suzuki, Norio Miyaura and Kinji Yamada reported SMC which is the most common method in the formation of carbon–carbon bond in drug discovery.^28,29^ In the context, the reaction of various aryl bromide derivatives 1 with commercially available aryl boronic acids 7 under action of palladium-catalyzed Suzuki coupling reaction on porous carbon nanospheres (Pd/CNS) afforded biphenyl derivatives 2. It is worthy to mention that, using (Pd/CNS) as catalyst is preferable than other Pd salts due to its highly effective, and lower cost (Scheme 12).^63^

**Scheme 13:**
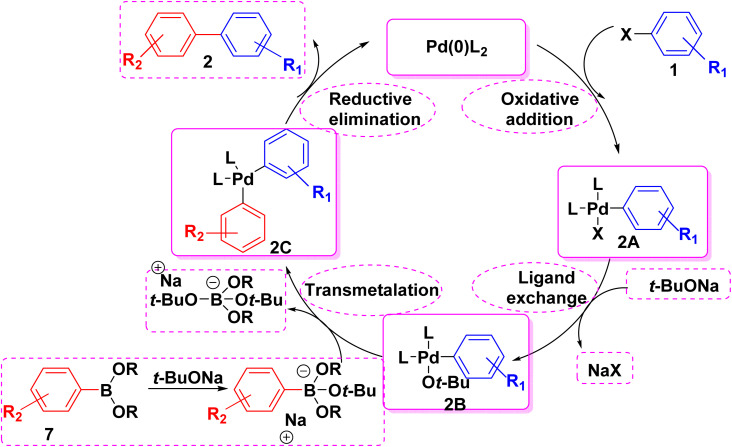
The postulated mechanism for biphenyl synthesis *via* Suzuki–Miyaura coupling reaction.

The simple mechanism for (SMC) reaction passed *through* three steps.^23,64^ The first step is the oxidative addition in which organo palladium(ii) complex 2A is formed *via* coupling of aryl halide 1 with palladium catalyst and this is happening *via* breaking carbon–halogen bond and by squeezing palladium between the aryl and halogen group. The second step is the transmetalation whereas organoboron compound is converted to nucleophilic borate in the presence of base such as sodium *tert*-butoxide and carbonate salts then attacking on a Pd(ii) complex 2B to form 2C complex. Finally, reductive elimination step in which the two aryl groups are reductively eliminated from Pd(ii) complex 2C and combine together to form C–C bond and the palladium is reformed (Scheme 13).^65,66^

**Scheme 14:**
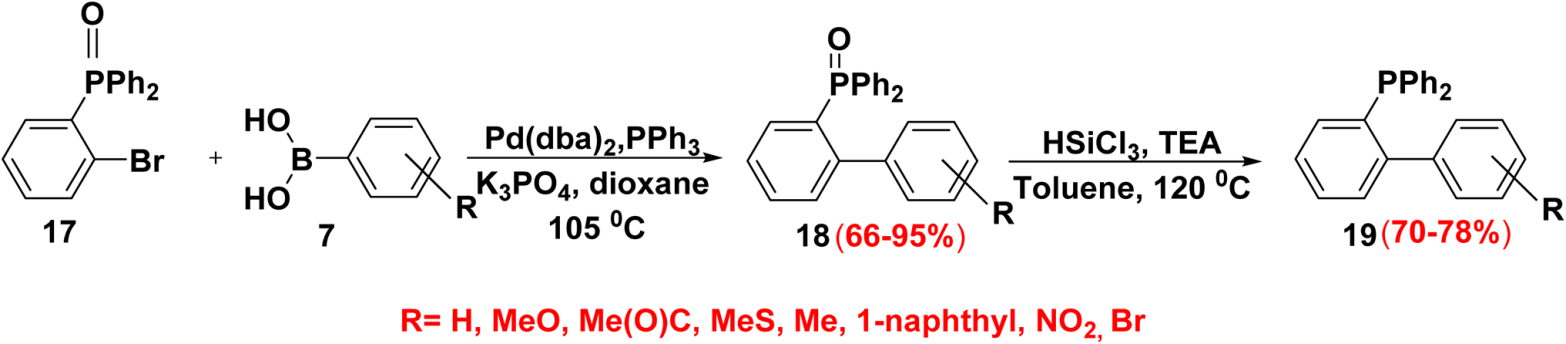
Synthetic pathway to generate biphenyl-based phosphine.

Biphenyl phosphine was effective ligand in metal catalyzed reaction as hydroformylation, asymmetric hydrogenation and allylic alkylation. Reaction of equimolar amount of (2-bromophenyl)diphenylphosphine oxide ((2-BrC_6_H_4_)OPPh_2_) 17 with aryl boronic acids 7 in the presence of bis(dibenzylideneacetone)palladium catalyst (Pd(dba)_2_), triphenylphosphine (PPh_3_) (4 equiv.) and potassium phosphate (K_3_PO_4_) (2 equiv.) as a base in refluxing dioxane at 105 °C yielded biphenyl phosphine oxide derivatives 18 followed by reduction with a mixture of trichlorosilane and catalytic drops of triethylamine (TEA) produced biphenyl phosphine derivatives 19 (Scheme 14).^67^

**Scheme 15:**
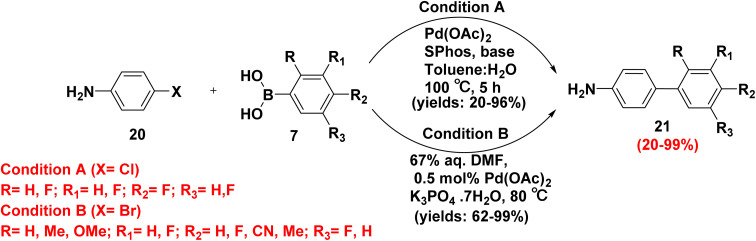
Preparation of amino biphenyl derivatives.

Substituted biphenyl anilines play a vital role in the synthesis of pharmaceutical, dyes, organometallic complexes and ferromagnetic materials due to the ability of amino group to react with aldehyde and ketone to produce Schiff products with strong electronic donor and versatile frameworks.^68^ Furthermore, treatment of 4-chloroaniline (20) with fluorinated phenyl boronic acid 7 in refluxing a mixture of toluene and water with a catalytic amount of palladium acetate (Pd(OAc)_2_) and bulky phosphine ligand (SPhos) yielded fluorinated aminobiphenyl derivatives 21.^69^ Analogously, refluxing of 4-chloroaniline (20) with fluorinated phenyl boronic acid 7 with DMF in the presence of catalytic Pd(OAc)_2_ and K_3_PO_4_ as a base at 80 °C afforded fluorinated aminobiphenyl derivatives 21 (Scheme 15).^68^

**Scheme 16:**
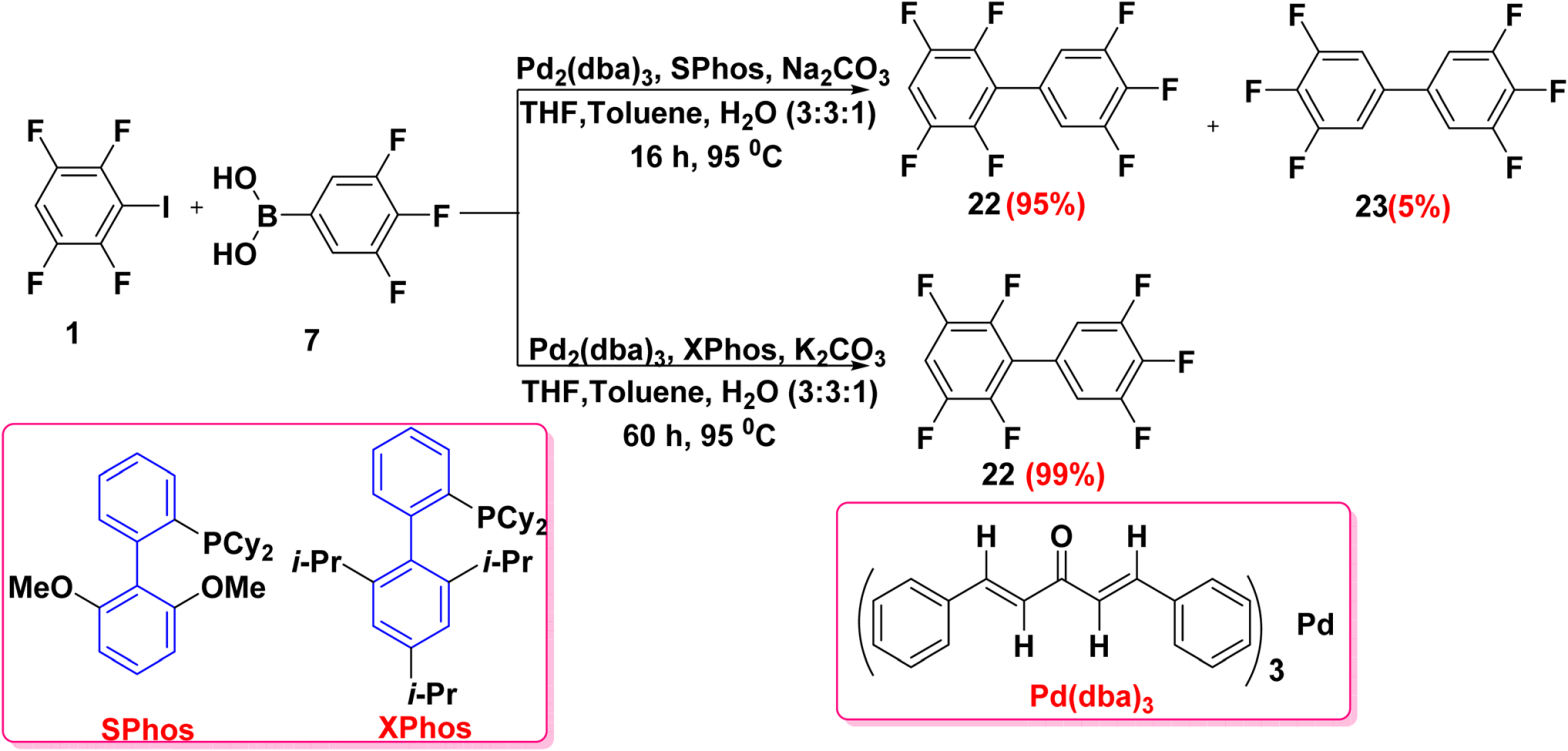
Preparation of fluorinated biphenyl *via* Suzuki–Miyaura cross coupling reaction.

Fluorinated biphenyls are scaffolds for various applications due to their rigidity, chemical stability and electron poor nature. Furthermore, fluorinated biphenyls are used to develop OLEDs, liquid crystal displays (LCDs), organic semiconductors, metal–organic frameworks (MOF), and organic polymer of intrinsic microporosity (OMIMs). In addition, organoflourine as substituents of biphenyl have become widespread drug motifs, as they affect nearly adsorption, metabolism, distribution and excretion properties of lead compounds.^70^ Bulfield *et al.*^71^ prepared a mixture of fluorinated biphenyl compounds 22, 23*via* Suzuki–Miyaura cross coupling reaction of tetrafluoroiodobenzene 1 with trifluoroboronic acid 7 in the presence of sodium carbonate (Na_2_CO_3_) as a base and tris(dibenzylideneacetone)dipalladium(0) (Pd_2_(dba)_3_) as a catalyst with SPhos as a ligand in refluxing with a mixture of THF, toluene, and H_2_O. Whereas, using XPhos as a ligand and potassium carbonate (K_2_CO_3_) as base afforded fluorinated biphenyl 22 only with an excellent yield (99%) (Scheme 16). The reactivity of aryl halides is dependent on the dissociation energy of the C–X bond according to (I > Br > Cl > F). As a larger halogen atom will be a better leaving group due to having a lower bond dissociation, thus increasing the reactivity. As a result of aforementioned information, boronic derivative 7 couples with iodide atom faster than fluorine atom (Scheme 16).^72^

**Scheme 17:**
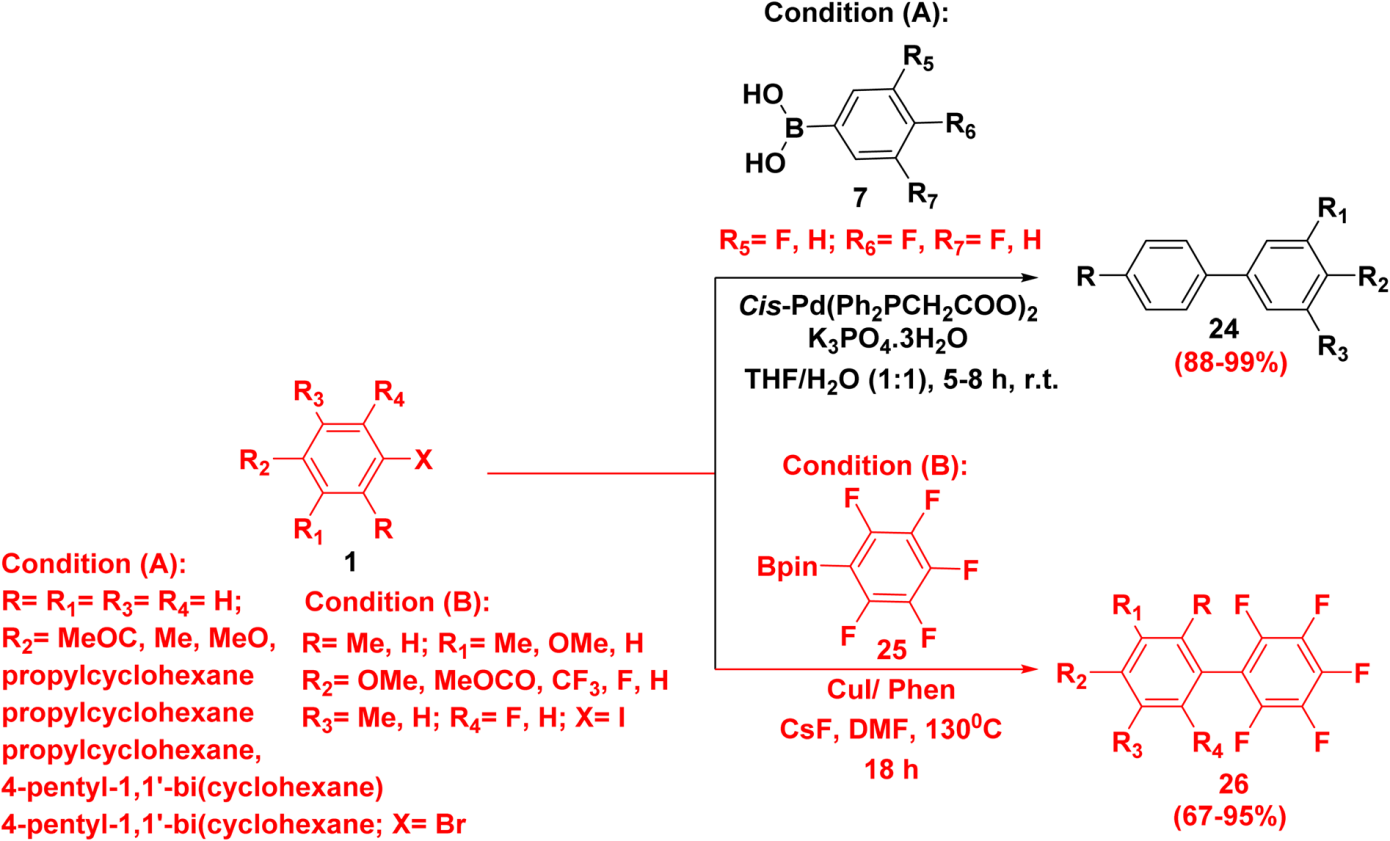
Formation of fluorinated biphenyls.

Whereby, reaction of aryl halide 1 with various fluorinated boronic acids 7 in the presence of palladium catalyst complex and K_3_PO_4_ as a base in a refluxing solvent mixture of tetrahydrofuran (THF) and water furnished fluorinated biphenyl 24.^6^ Additionally, fluorinated biphenyl 26 was synthesized by refluxing iodobenzene 1 and arylboronate 25 in Cu catalyst, phenanthroline ligand (phen) and DMF (Scheme 17).^73^

**Scheme 18:**
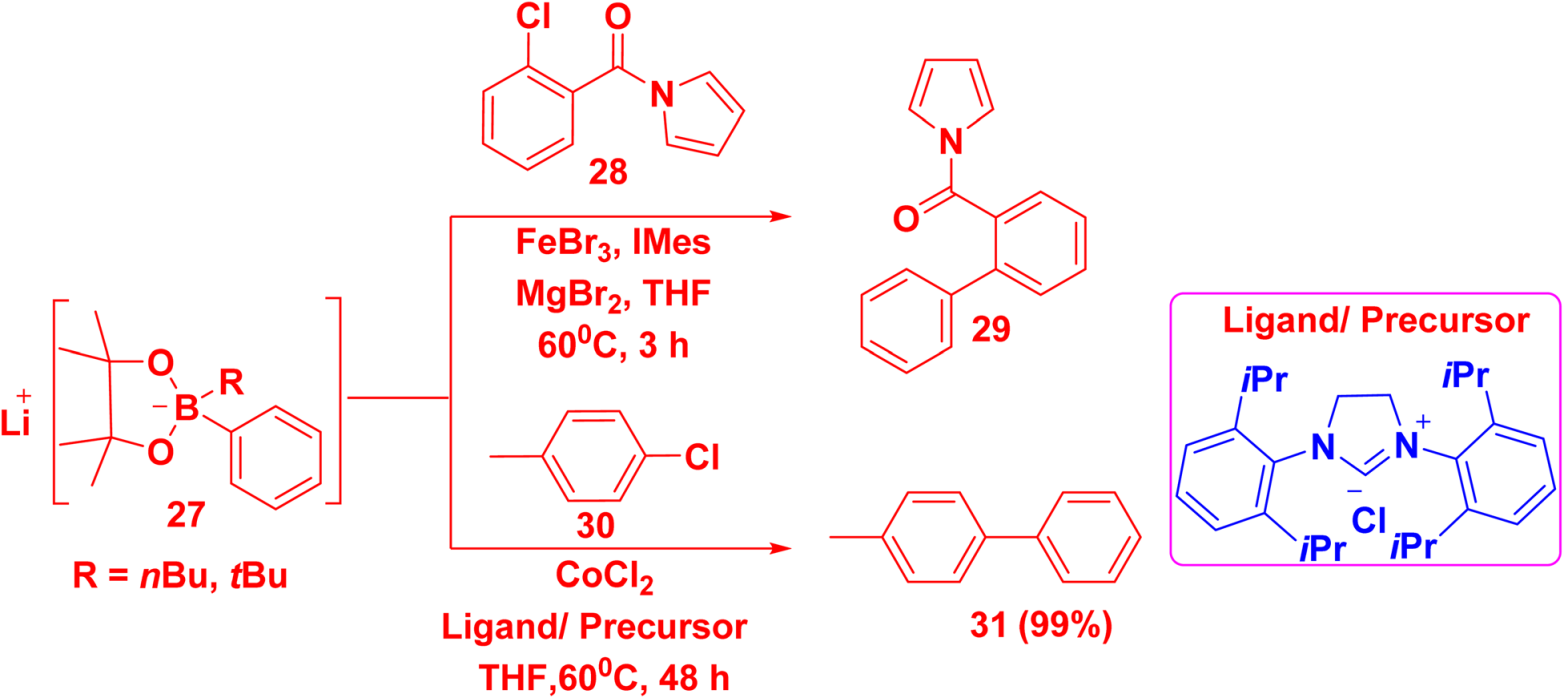
Iron catalyzed Suzuki–Miyaura cross coupling reaction for biphenyl synthesis.

Palladium catalyst was predominantly used in Suzuki coupling reaction, but due to its scarcity and high price, it was replaced by Ni and Co catalyst. Cobalt salts have stronger catalytic activity and a reduced tendency to yield homocoupling by-products. However, iron was considered the best substitute for a Pd catalyst because it was the least toxic and the most abundant transition metal.^74^ Biphenylpyrrole derivative 29 was obtained *via* the reaction of arylhalide 28 with lithium arylboronates 27 in the presence of Fe catalyst and 1,3-bis(trimethylphenyl)-1,3-dihydro-2*H*-imidazol-2-ylidene (IMes) as a ligand.^75^ Whereas, the reaction of chloromethylbenzene 30 with 27 in the presence of cobalt(ii) chloride (CoCl_2_) and a ligand/precursor named bis(2,6-diisopropylphenyl)-1*H*-imidazol-3-ium afforded methylbiphenyl 31 (Scheme 18).^76^

**Scheme 19:**
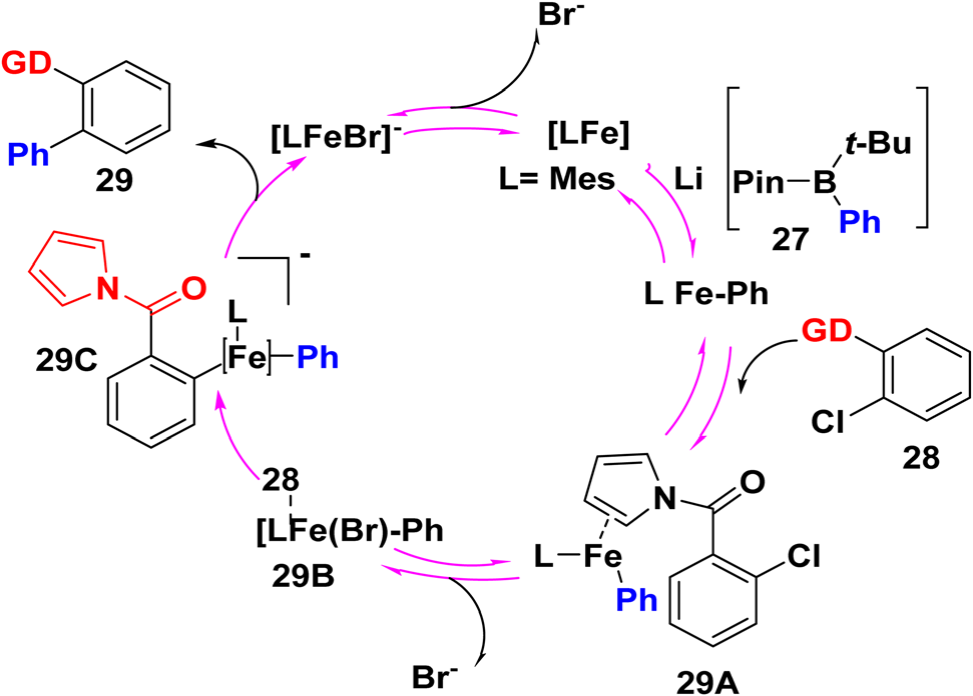
The suggested mechanism for biphenylpyrrole synthesis.

The suggested mechanism involved the formation of a low-valent molecular iron complex that engages in the reversible coordination of both bromide and the pyrrole-containing substrate 28. The C–Cl bond in 28 is reductively activated by the resulting ferrate complex. The biphenylpyrrole product 29 was delivered by reductive elimination, and the active catalyst was renewed by bromide abstraction (Scheme 19).^75^

**Scheme 20:**
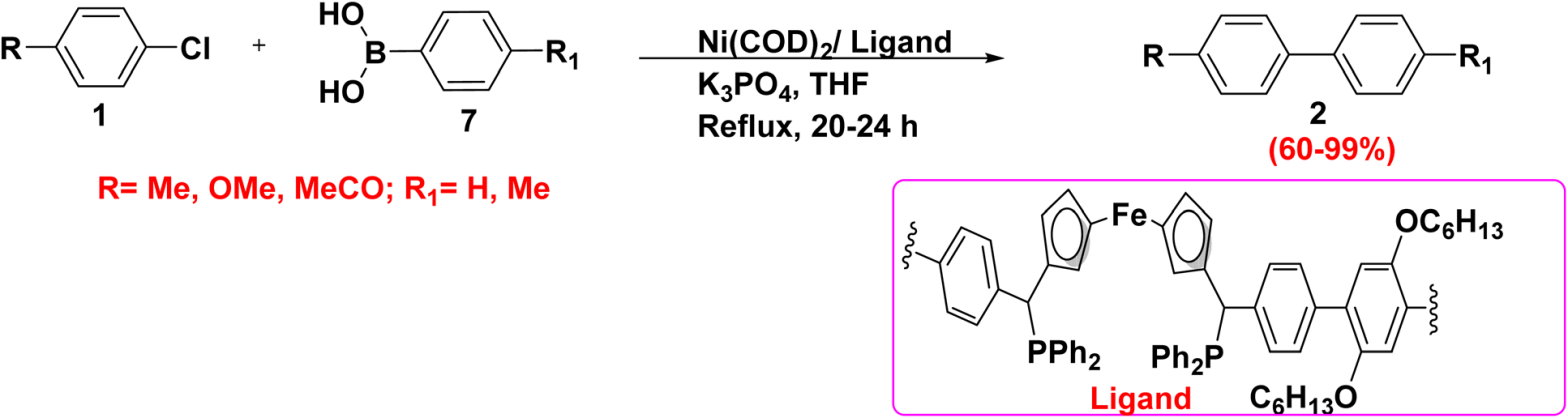
Synthesis of biphenyl derivatives 2*via* nickel catalyzed SMC.

Additionally, nickel catalyzed Suzuki coupling reaction of chlorobenzene derivative 1 with substituted phenylboronic acid 7 in the presence of Ni catalyst, ferrocenylmethylphosphines derivative as a ligand, K_3_PO_4_ and THF afforded biphenyl derivative 2 (Scheme 20).^77^

**Scheme 21:**
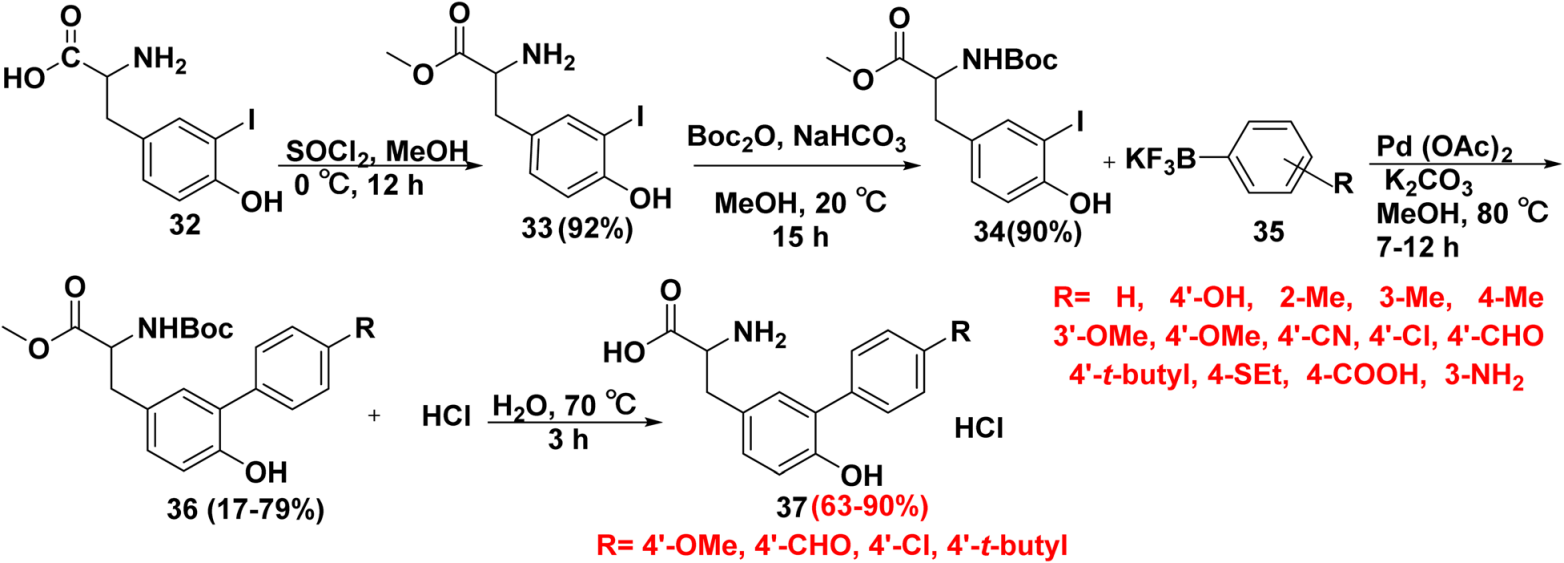
Synthetic strategy for preparation of biphenyl tyrosine using aryl trifluoroborate salts.

Biphenyl tyrosine derivatives are biologically active molecules including arylomycin A_2_ act as an antibacterial drug. Tyrosine derivatives were keys for the synthesis of biaryl derivatives *via* SMC reaction. Nevertheless, tyrosine compounds must be protected in order to react. 3-Iodo tyrosine (32) was esterified using methanol (MeOH) with thionyl chloride (SOCl_2_) to afford 2-amino-3-iodophenyl-propanoate 33. Then, the amino group was protected using *tert*-butyloxycarbonyl (*t*-Boc) and sodium bicarbonate (NaHCO_3_) in MeOH to furnish *N-tert*-butyloxycarbonyl-3-iodotyrosine methyl ester (34). Refluxing *N-tert*-butyloxycarbonyl-3-iodotyrosine ester 34 in the presence of Pd(OAc)_2_, K_2_CO_3_ and hetero aryl trifluoroborate salt 35 in MeOH to afford *t*-butoxycarbonyl-(6-hydroxy-[biphenyl]-yl)propanoate derivatives 36. Finally, deprotection of compounds 36 was achieved *via* heating them with dil. HCl at 70 °C for 3 h to give the free amino acid 37 (Scheme 21).^4^

**Scheme 22:**
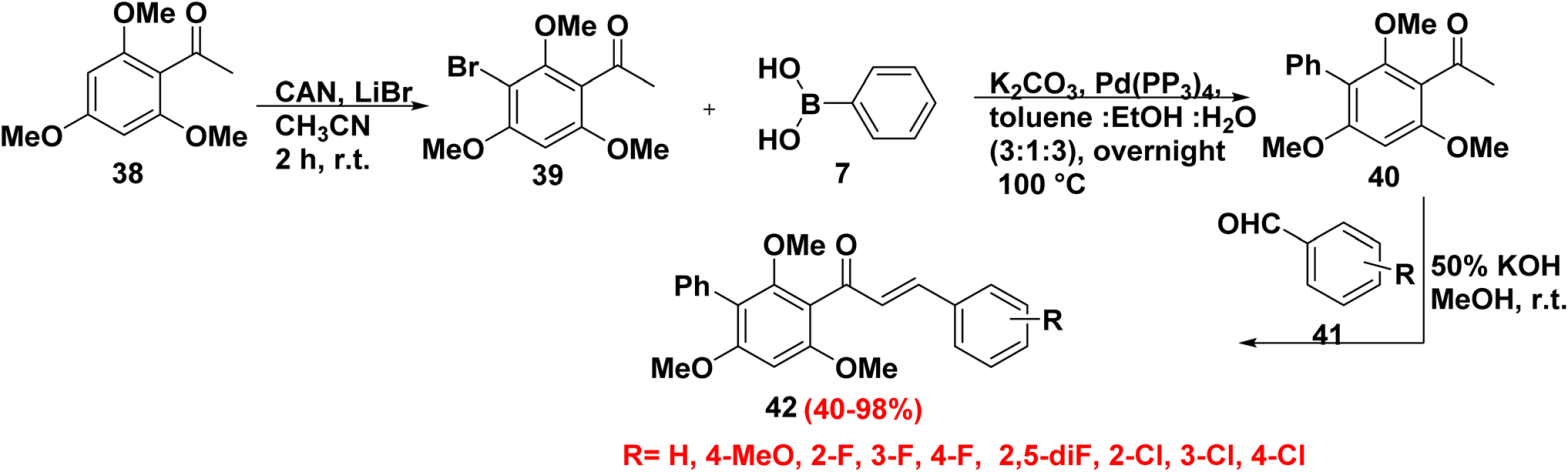
Formation of biphenyl chalcone derivatives by SMC.

Burmaoglu *et al.*^78^ synthesized new biphenyl chalcone derivatives 42 according to the reaction sequences. First, preparation of 3-bromo-2,4,6-trimethoxyacetophenone (39) *through* bromination reaction of 2,4,6-trimethoxy acetophenone (38) with ceric ammonium nitrate (CAN) and lithium bromide (LiBr) in acetonitrile (CH_3_CN), followed by applying SMC of compound 39 with phenylboronic acid (7) in the presence of Pd(PPh_3_)_4_ and K_2_CO_3_ in a mixture of ethanol (EtOH) and toluene afforded 2,4,6-trimethoxy-[1,1′-biphenyl]ethan-1-one 40 (Scheme 22). Claisen–Schmidt condensation reaction of substituted biphenyl 40 and benzaldehydes 41 yielded biphenyl chalcone derivatives 42 (Scheme 22). Consequently, biphenyl-substituted chalcone scaffolds which are classified as privileged metabolic enzyme inhibitor motifs which essential support in the treatment of Alzheimer's disease (AD) and glaucoma due to hydrophobic effect and their ability to bind with multiple receptors.

**Scheme 23:**
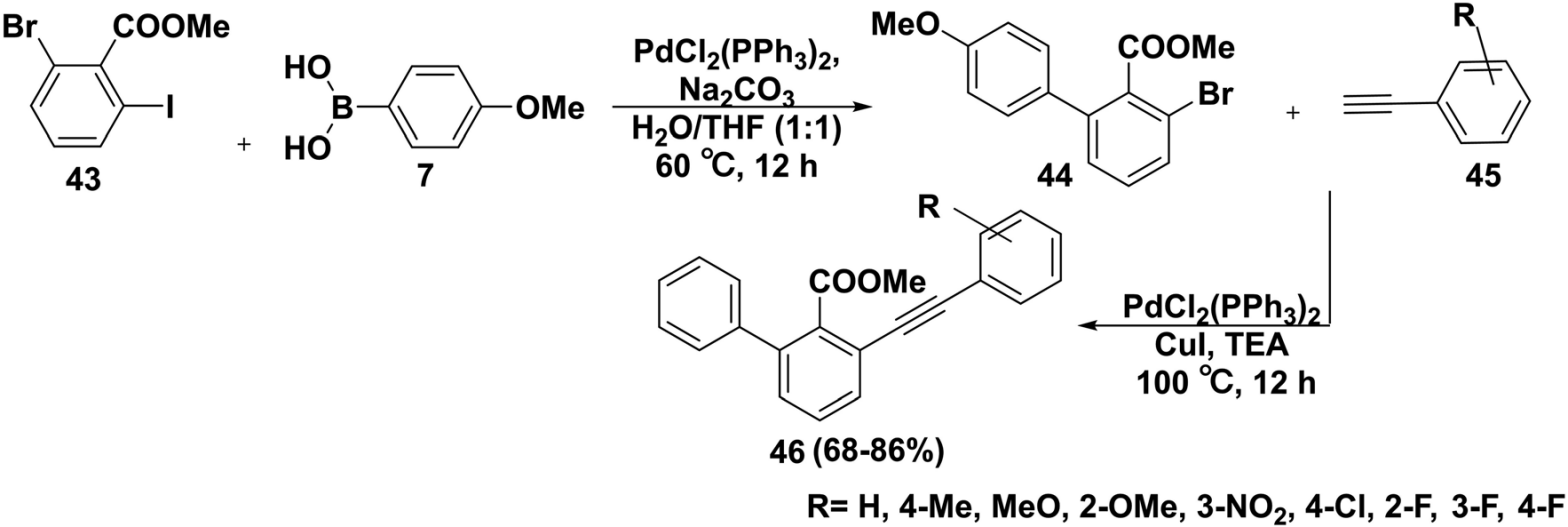
Synthesis of phenylacetylene derivatives.

Treatment of methyl 2-bromo-6-iodobenzoate (43) with (4-methoxyphenyl)boronic acid (7) in the presence of Na_2_CO_3_, bis-triphenylphosphine palladium dichloride (PdCl_2_(PPh_3_)_2_), and a mixture of H_2_O and THF afforded methyl-3-bromo-4′-methoxy-[1,1′-biphenyl]-2-carboxylate (44). In order to produce biphenyl acetylene derivatives 46, compound 44 was reacted with phenylacetylene derivatives 45 ^79^ (Scheme 23). Diphenylacetylene-based all-*trans* retinoic acid (ATRA) analogues with enhanced stability may hold tremendous promise as therapies for large number of cancers and neurological conditions.^80^ In addition, diphenylacetylene derivatives acted as antimicrobial peptide mimetics.^81^

**Scheme 24:**
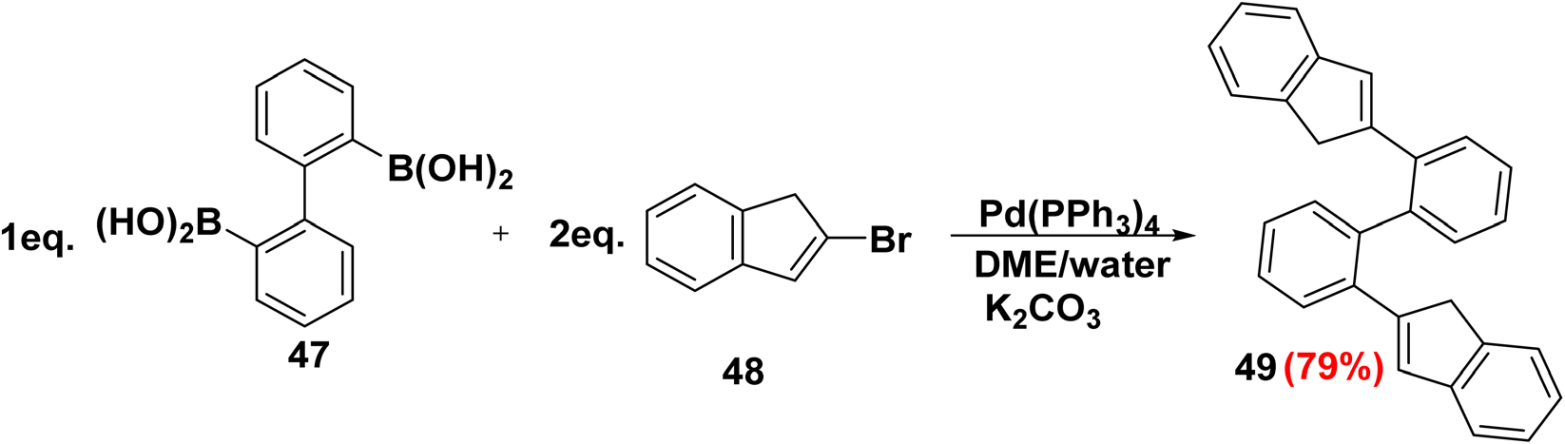
Synthesis of (1*H*-inden-2-yl) biphenyl *via* palladium-catalyzed Suzuki–Miyaura coupling reaction.

Over the past decade, olefinic polymerization which catalyzed by transition metals has great prominence in both academic and industrial research.^82,83^ Due to the recent adoption of high-throughput screening (HTS) methods, the field of olefin polymerization facilitated by Ziegler–Natta catalysts is particularly active.^84^ A significant number of novel nonmetallocene ligands have been discovered using these techniques for polymerization catalysis. Application of SMC of 2,2′-biphenyldiboronic acid 47 with 2-bromoindene 48 in the presence of tetrakis(triphenylphosphine)palladium [Pd(PPh_3_)_4_] furnished polycyclic 2,2′-di(1*H*-inden-2-yl)-1,1′-biphenyl (49) which showed promising activities in olefin metathesis polymerization (Scheme 24).^85^

**Scheme 25:**
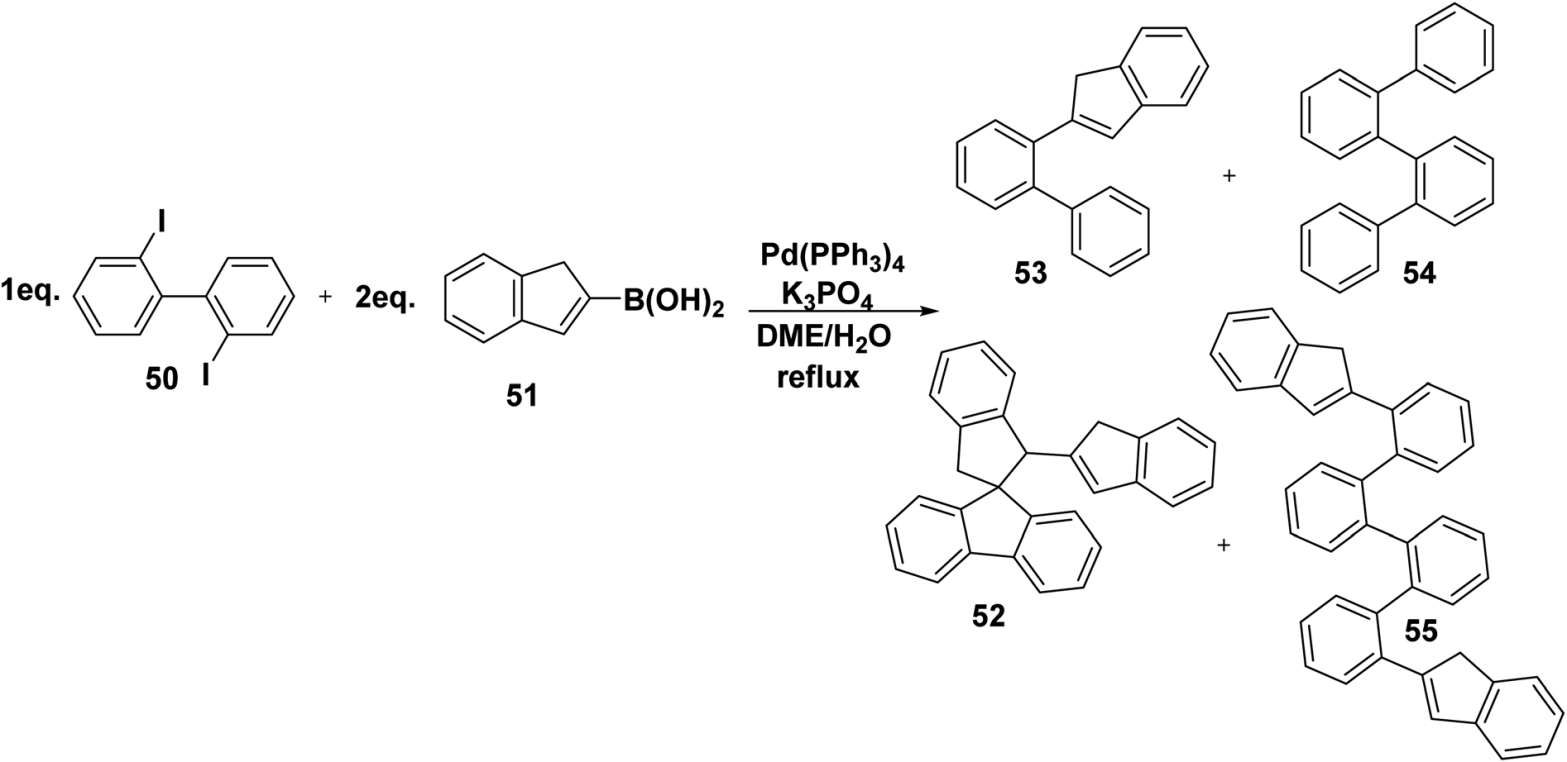
Synthesis of polycyclic biphenyl compounds.

Polyaromatic hydrocarbons are distinguishable class of aromatic compounds. As aromatic polyphenyl scaffolds give a crowded molecule that usually relieves the congestion by distortion from planarity displaying highly reversible electrochemical properties as this merit shared by a few organic compounds.^86^ Whereas, a mixture of four different polycyclic products 52–55 were furnished by reaction of 2,2′-diiodo-biphenyl 50 with (1*H*-inden-2-yl)boronic acid (51) in the presence of palladium catalyst [Pd(PPh_3_)_4_] and K_3_PO_4_ in refluxing DME/H_2_O (Scheme 25).^85^

**Scheme 26:**
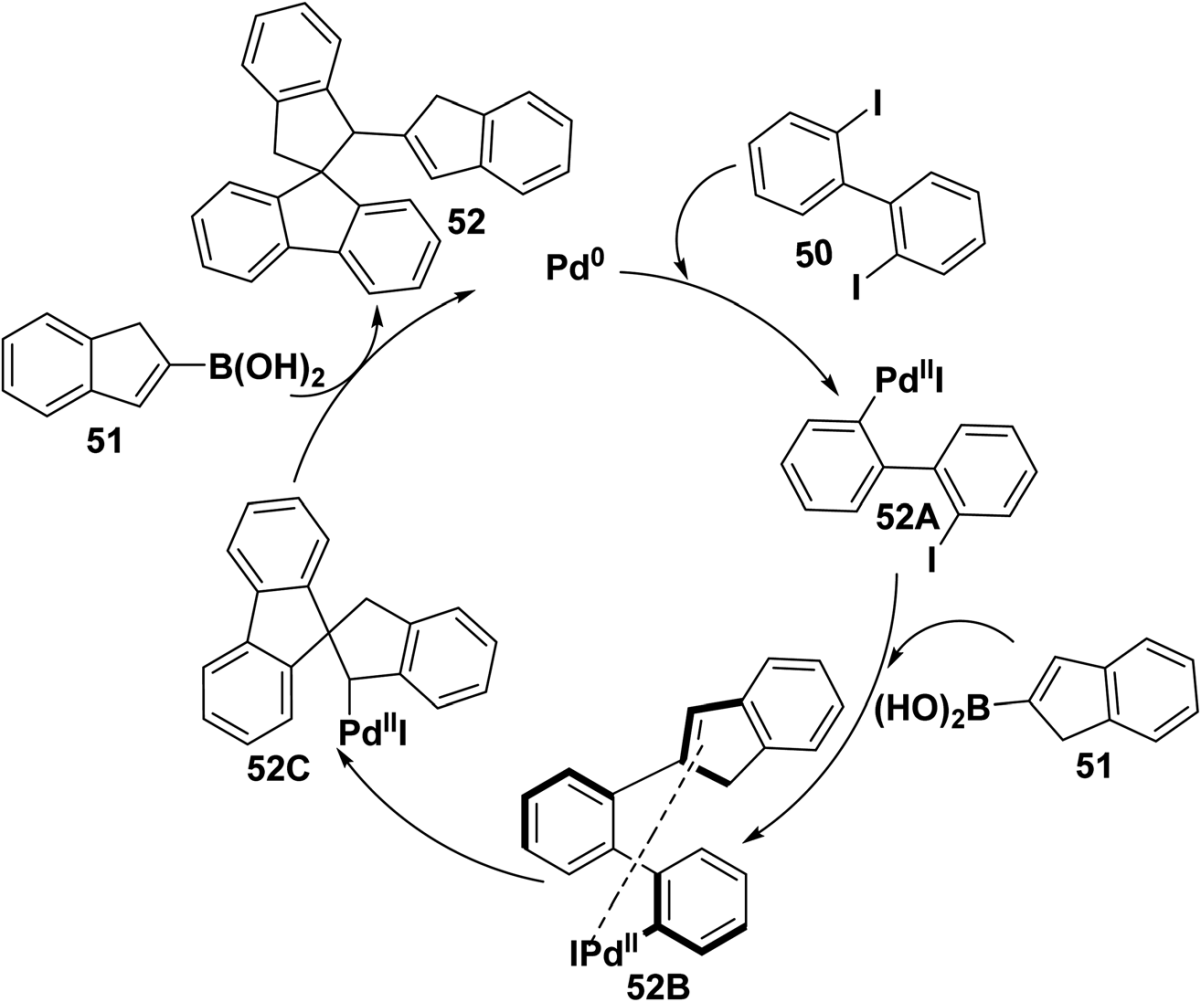
The proposed mechanism for the formation of polycyclic product 52.

Therefore, a proposed mechanism for the formation of 1*H*-inden-1′,3′-dihydrospiro[fluorene-9,2′-indene] 52 is depicted in Scheme 26. Initially, a palladium catalyst is inserted between C–I bond, then (1*H*-inden-2-yl) boronic acid (51) is coupled with (2′-iodo-[1,1′-biphenyl]-2-yl) palladium(ii)iodide (52A), followed by intramolecular Heck reaction from 52B to 52C. Finally, Suzuki coupling reaction of (1′,3′-dihydrospiro[fluorene-9,2′-inden]-1′-yl) palladium(ii) iodide (52C) with (1*H*-inden-2-yl) boronic acid (51) to afford 1*H*-inden-2-yl-dihydrospiro[indene] 52 with 58% yield (Scheme 26).^85^

**Scheme 27:**
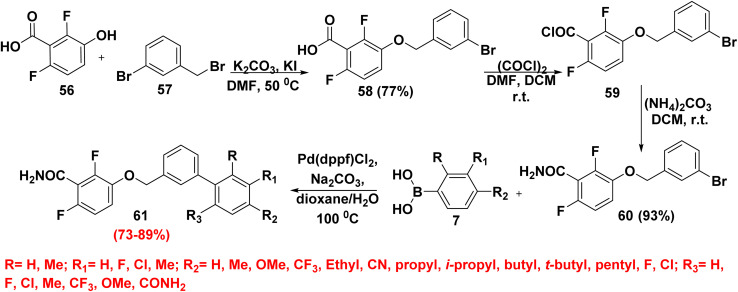
Formation of biphenyl benzamide derivatives.

Biphenyl benzamide derivatives inhibit the filamenting temperature-sensitive mutant Z (FtsZ) protein, in addition to having antibacterial activities.^87^ Firstly, 2,6-difluoro-3-hydroxybenzoic acid (56) was alkylated with 3-bromobenzyl bromide (57) in the presence of sodium iodide (KI) and K_2_CO_3_ to furnish fluorinated acid derivative 58. Then, fluorinated acid derivative 58 was reacted with oxalyl chloride (COCl)_2_ in the presence of a mixture of dimethylformamide and dichloromethane (DMF/DCM) to afford difluorobenzoyl chloride derivative 59. The acid was transformed to benzamide 60 by using oxalyl chloride, then treatment with ammonium carbonate ((NH_4_)_2_CO_3_). Finally, biphenyl benzamide derivatives 61 were produced in a wide range of yields (73–89%) *via* coupling commercially available boronic acids 7 with compound 60 (Scheme 27).^87^

**Scheme 28:**
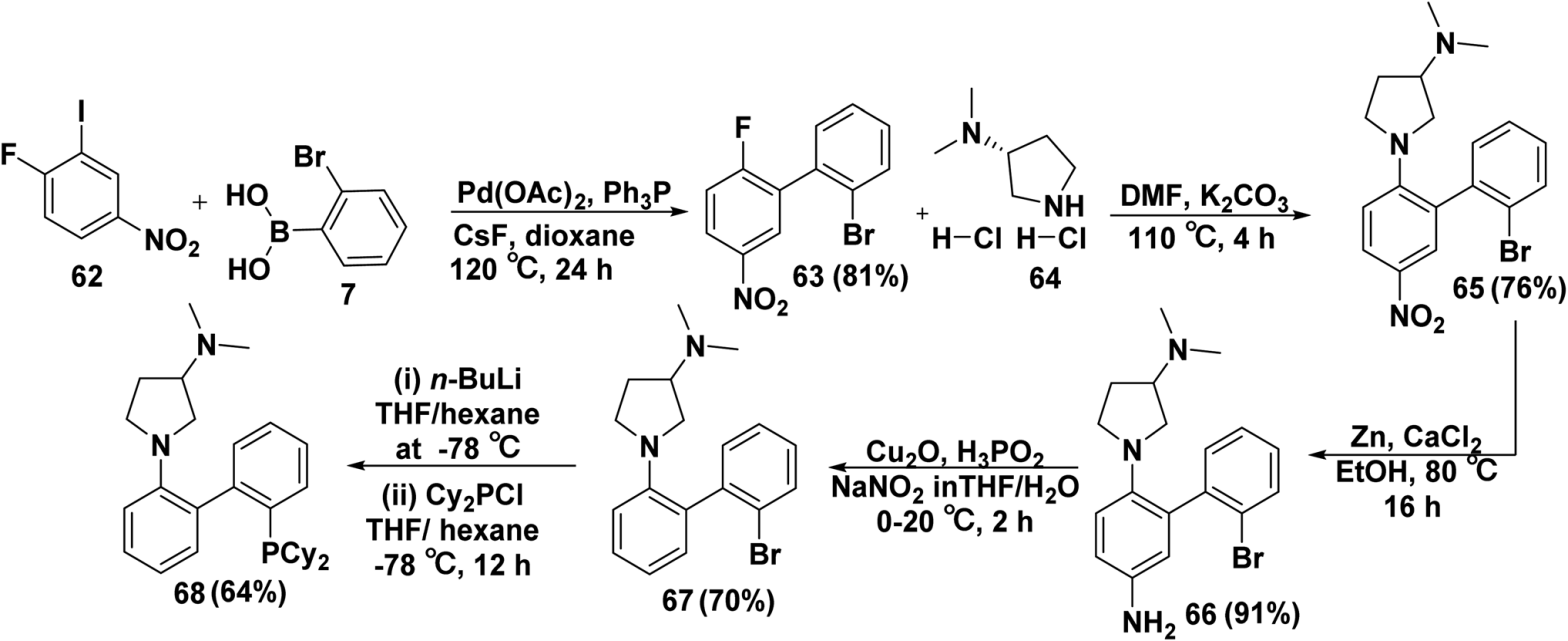
Synthesis of *N*-biphenyl pyrrolidine derivatives.

*N*-Biphenyl pyrrolidine derivatives are employed as ligand in arylaminations that are catalyzed by palladium. Furthermore, 2′-bromo-2-fluoro-5-nitro-1,1′-biphenyl (63) were produced in (81% yield) *via* Suzuki–Miyaura cross coupling between 2-iodo 4-nitro fluorobenzene (62) and boronic acid 7 in the presence of palladium catalyst and PPh_3_ in refluxing dioxane. Then, the fluorine atom was nucleophilically substituted by (*R*)-(3-*N*,*N*-dimethylamino)pyrrolidine (64) in the presence of K_2_CO_3_ in DMF to yield 1-(2′-bromo-5-nitro-[1,1′-biphenyl]-2-yl)-*N*,*N*-dimethylpyrrolidin-3-amine (65). (*R*)-[1-(2′-Bromo-5-amino-biphenyl]-2-yl)-pyrrolidin-3-yldimethylamine (66) was produced by refluxing zinc powder and calcium chloride (CaCl_2_) with compound (*R*)-65 in EtOH. After that, hypophosphorous acid (H_3_PO_2_) was added to compound (*R*)-66 in a mixture of THF and H_2_O, followed by treatment with cupper(i)oxide, and sodium nitrite (NaNO_2_) furnished bromo(biphenyl-2-yl)-dimethylpyrrolidin-3-amine 67. Finally, 2′-(dicyclohexylphosphaneyl)-[1,1′-biphenyl]-2-yl-*N*,*N*-dimethylpyrrolidin-3-amine 68 was obtained in two steps involving adding *n*-butyl lithium dropwise to compound (*R*)-67 in a mixture of THF and hexane as first step. In the second step, chlorodicyclohexylphosphane (Cy_2_PCl) was added with stirring to replace bromide by dicyclohexylphosphane group (Scheme 28).^88^

**Scheme 29:**
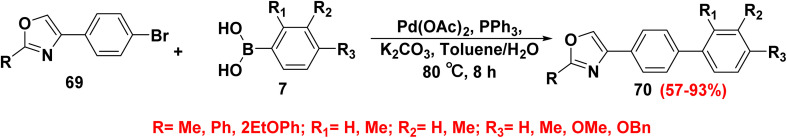
Suzuki coupling reaction for synthesis of biphenyl oxazole derivatives.

The synthesis of biphenyl oxazole derivatives 70 was reported by Ahmad *et al.*^89^ under mild conditions which were enzyme inhibitors and estimated as therapeutic seeds for ailments associated with NPP1 and NPP3 isozymes *via* Suzuki–Miyaura cross coupling of bromo-phenyloxazole 69 with various boronic acids 7 in a mixture solvent system (toluene/water) (Scheme 29).

**Scheme 30:**
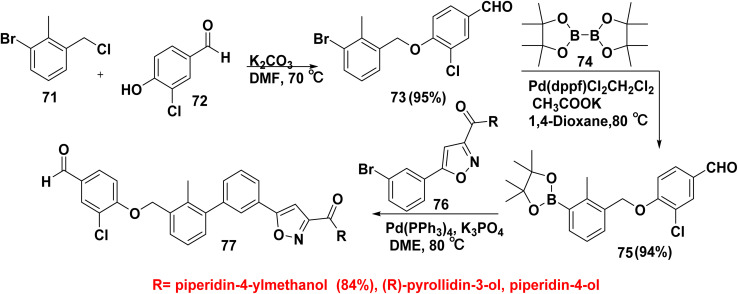
Synthesis of biphenyl oxazole derivatives.

Moreover, an efficient synthesis of biphenyl oxazole derivatives 77 was achieved *through* alkylation reaction of 1-bromo-3-(chloromethyl)-2-methylbenzene (71) with 3-chloro-4-hydroxybenzaldehyde (72) produced 4-((3-bromo-2-methylbenzyl)oxy)-3-chlorobenzaldehyde (73). Next, refluxing 4-((3-bromo-2-methylbenzyl)oxy)-3-chlorobenzaldehyde (73) with bis(pinacolato)diboron (B_2_pin_2_) (74) in 1,4-dioxane and potassium acetate (CH_3_COOK) in the presence of 1,1′-bis(diphenylphosphino)ferrocene-palladium(ii)dichloride diCrClchloromethane complex [pd(dppf)Cl_2_CH_2_Cl_2_] afforded 3-chloro-4-((2-methyl-3-(tetramethyl-1,3,2-dioxaborolan-2-yl)benzyl)oxy)benzaldehyde (75). Finally, compound 75 was reacted with (3-bromophenyl)isoxazole derivatives 76*via* Suzuki coupling reaction yielded biphenyl oxazole derivatives 77 (Scheme 30).^90^

### 2.7 Stille cross coupling

**Scheme 31:**
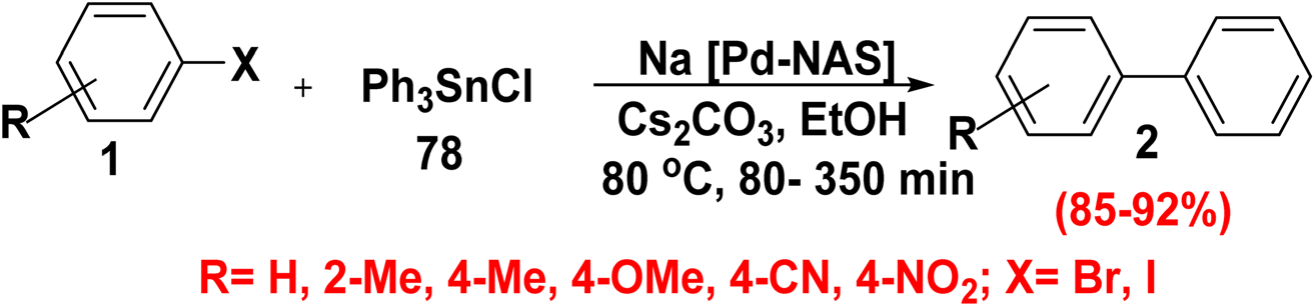
Synthesis of parent biphenyl *via* Stille cross coupling.

Stille cross coupling reaction is one-step coupling reaction for nucleosides synthesis was first reported in 1986.^26,91^ Stille cross coupling is still a significant reaction to form C–C bond, and one of the most selective and general methods for palladium catalyzed cross coupling reaction. Therefore, it was constituted to produce a various ring system bearing functional groups and to synthesize several alkenes, alkenyls, oligoarylenes and biaryls.^92–94^ The interesting advantage of Stille reaction is the using of organotin compounds which are mild reagents and tolerate numerous of functional groups. On the other hand, the only drawback is the water insoluble tin reagents and toxicity of the hydrophobic.^92^ Stille cross coupling reaction of aryl halide 1 with triphenyl tin chloride (Ph_3_SnCl) (78) in the presence of catalytic amount of palladium grafted on natural asphalt sulfonate Na[Pd-NAS], cesium carbonate (Cs_2_CO_3_) as a base in refluxing ethanol furnished biphenyl derivatives 2 (Scheme 31).^88,95^

**Scheme 32:**
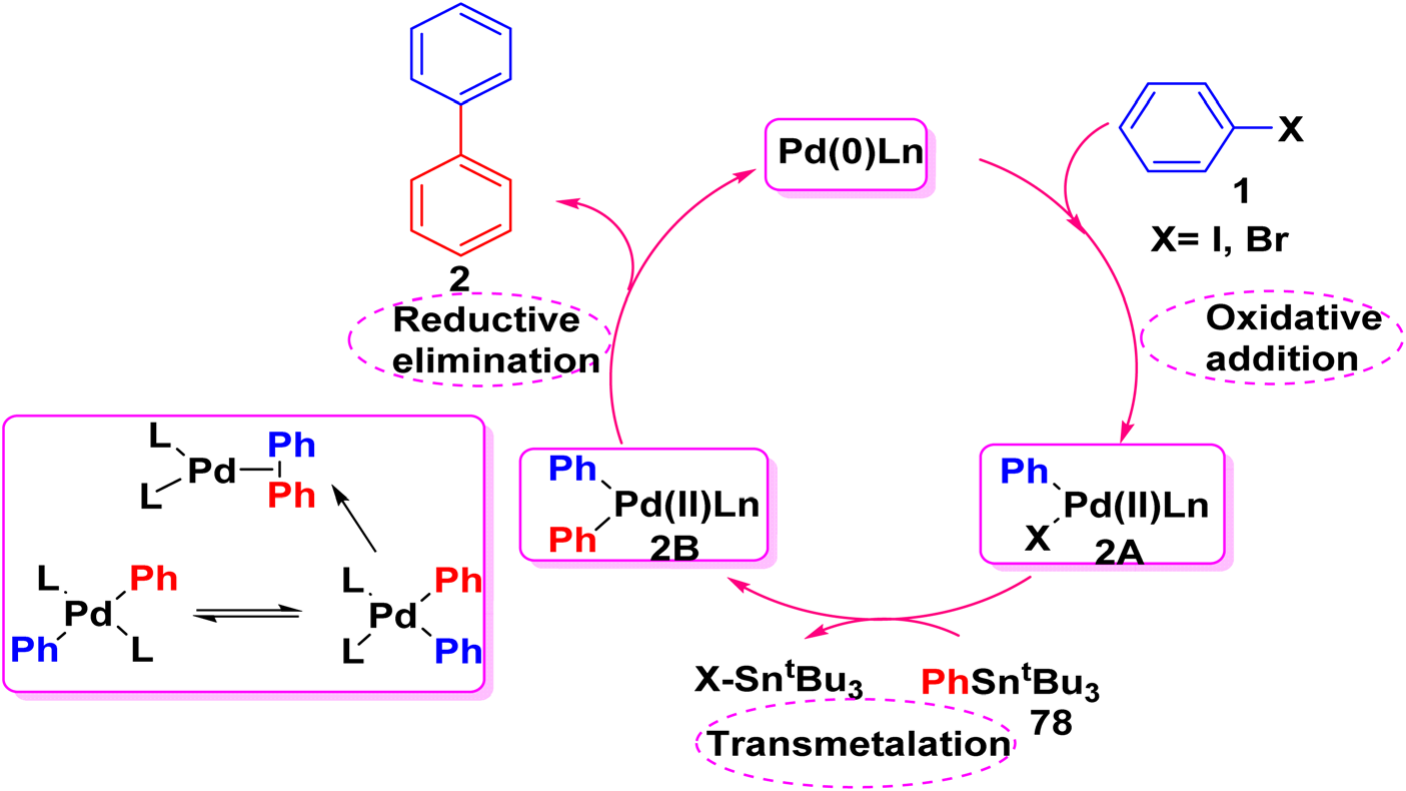
The proposed mechanistic pathway for Stille cross coupling reaction.

In 1986, Chemie John K. Stille described the possible mechanism for Stille cross coupling reaction. Stille cross coupling mechanism passed *through* three steps: oxidative addition, transmetalation and reductive elimination. In oxidative addition step, the catalytic species Pd(0)Ln is reacted with aryl halide 1 to form Pd(ii) complex 2A. In transmetalation step, Pd(ii) complex 2A cleavage the C–Sn bond of organotin reagent 78 to produce complex 2B. Finally, reductive elimination step for forming C–C bond and the catalyst is regenerated at the same time (Scheme 32).^96^

### 2.8 Negishi cross coupling

**Scheme 33:**
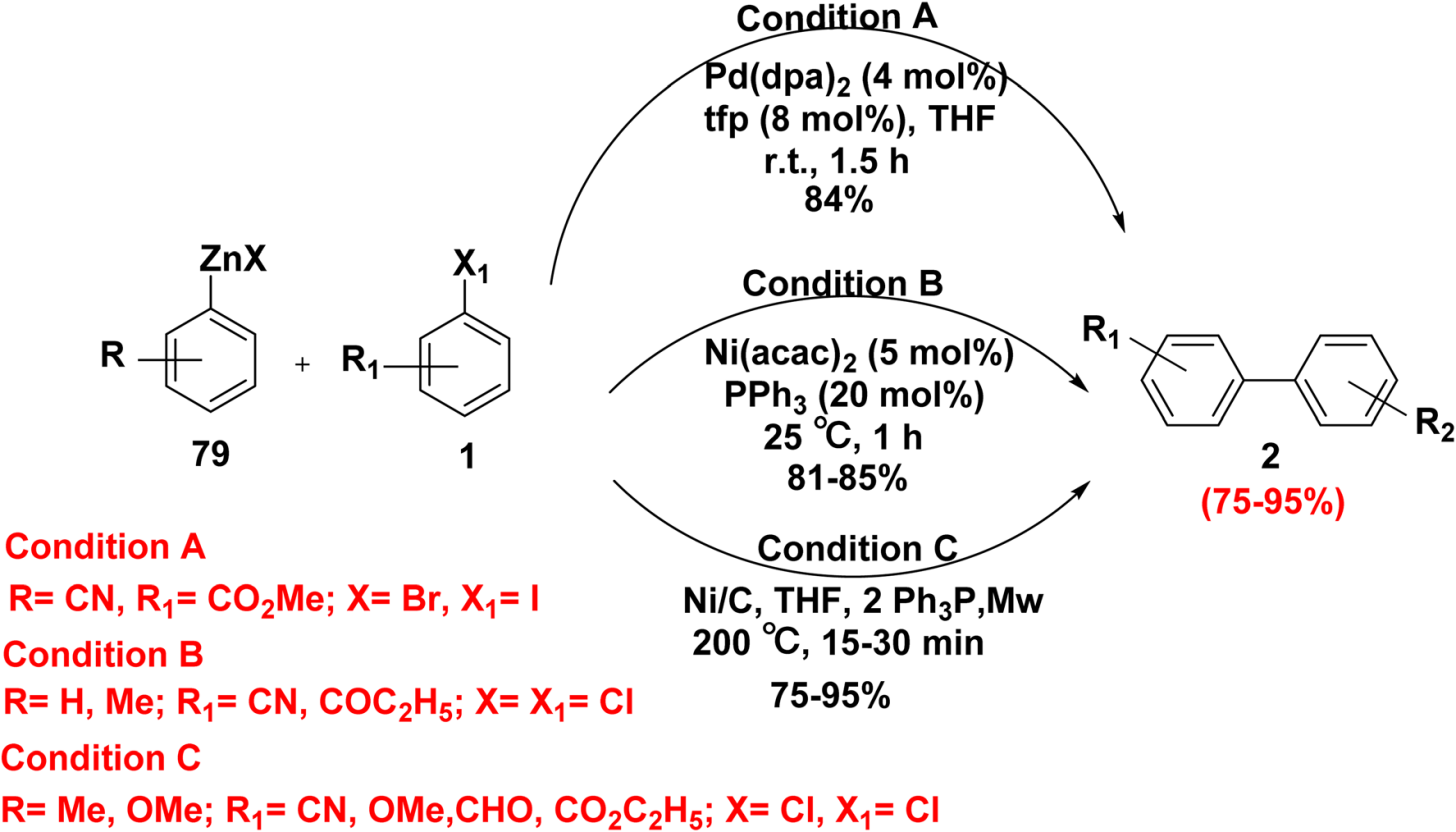
Synthesis of biphenyl derivatives *via* Negishi cross coupling reaction.

Negishi cross coupling reaction among the most important in organic chemistry for forming C–C bond between electrophiles with organozinc reagents and organic halides. Negishi reported an easy regio- and chemoselective method for the synthesis of unsymmetrical biaryl using an aryl–aryl coupling process that is catalyzed by nickel salts.^25,97^ Treatment of cyanophenyl zinc bromide 79 with *o*-iodobenzoate 1 in the presence of palladium bis(dibenzylideneacetone) (Pd(dba)_2_) and tris-*o*-furylphosphine (tfp) furnished biphenyl derivatives 2.^98^ In 2009, Phapale and Cárdenas^99^ discovered that Ni-based catalysts (Ni(acac)_2_/PPh_3_) facilitate coupling between aryl zinc chlorides 79 and haloarenes 1 affording biphenyl derivatives 2. The reaction of aryl zinc halide 79 and aryl halide 1 in the presence heterogeneous Ni catalyst on charcoal and microwave irradiation afforded biphenyls 2 in acceptable yields (75–95%) (Scheme 33).

**Scheme 34:**
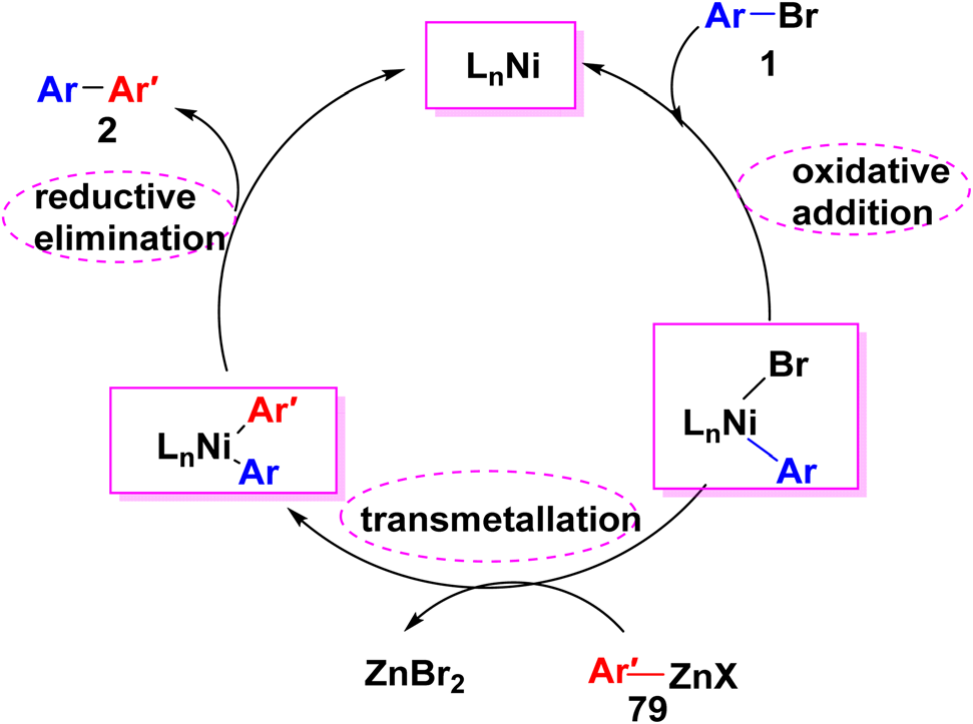
Plausible mechanism of the formation of biphenyl derivatives *via* Negishi cross coupling reaction.

Mechanism of Negishi cross coupling reaction passed *through* three steps. Initially, an organohalide or pseudohalide (such as triflate) is oxidatively added to a low valent metal complex to produce an organometallic derivative with a higher formal oxidation state on the metal center. Then, producing diorganometal species is processed, whereby, the nucleophilic carbon is transmetalate from the nucleophile to the transition metal complex. Finally, a new C–C bond is formed by further reductive elimination, which reproduces the catalytically active species (Scheme 34).^99^

### 2.9 Ball-milling method

**Scheme 35:**
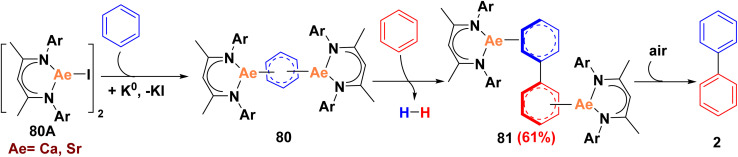
Dehydrogenative benzene–benzene coupling for biphenyl synthesis.

Recently, direct mechanocatalysis in ball-milling as a highly efficient synthetic tool applied for the cocondensation of alkali metals earth (Ae) such as K, Cs or Rb with benzene afforded C_6_H_5_˙^−^ salts and radical coupling to (biphenyl)^2−^ companied with emission of H_2_ gas. Similarly, the direct dehydrogenative coupling of benzene through low-valent alkaline-earth metals (Ae) intermediates was achieved *via* formation of metal (Ae) complex with a C_6_H_5_^2−^ dianion which acts as reducing agent to bulky β-diketiminate ligand BDI 80A to yield [(^DIPeP^BDI)Ca]_2_(biphenyl) 81 as dianionic *N*,*C*-chelating ligand, which rapidly decomposed to afford biphenyl scaffold 2 (Scheme 35).^100^

## 3. Chemical reactions

### 3.1 The Friedel–Crafts

Friedel–Crafts reactions are subdivided into two fundamental types: alkylation and acylation reactions.^101^ Whereby, ionic liquids have already been used in a number of Friedel–Crafts processes, which could significantly reduce the associated environmental concerns.^102^

#### 3.1.1 Friedel–Crafts alkylation of biphenyl with haloalkane

**Scheme 36:**
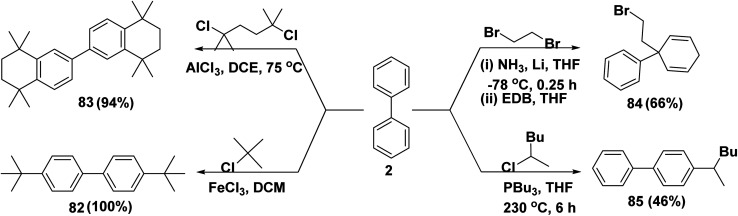
Friedel–Crafts alkylation of biphenyl with haloalkane.

For more than a century, Friedel–Crafts alkylation reaction is a vital reaction for the synthesis of alkyl substituted aromatic compounds.^103^ 4,4′-Di-*tert*-butylbiphenyl (82) was synthesized from biphenyl (2), which then, in the presence of catalytic amounts of anhydrous ferric chloride (FeCl_3_), interacted with *tert*-butyl chloride in halogenated solvents.^104^ In the same manner, Friedel–Crafts cycloannulation process produced octamethyl-octahydro-2,2′-binaphthalene 83*through* adding catalytic amounts of anhydrous AlCl_3_ to a solution of biphenyl (2) in 1,2-dichloroethane (DCE) followed by dropwise addition of readily available 2,5-dichloro-2,5-dimethylhexane.^105^ In the same context, a mixture of ammonia and lithium was added dropwisely to a solution of biphenyl (2) in THF till brick red suspension was formed. Then, a quick addition of 1,2-dibromoethane (EDB) was added to afford 2-bromoethyl-1,1′-biphenyl 84.^106^ A mixture of biphenyl (2), 2-chlorohexane and tributyl phosphine (PBu_3_) was heated for 6 h to afford 4-(hexan-2-yl)-1,1′-biphenyl (85) (Scheme 36).^103^

**Scheme 37:**
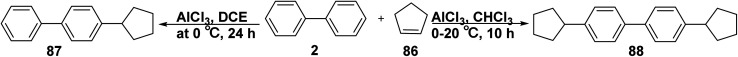
Reaction of biphenyl with cyclopentene.

Moreover, treatment of biphenyl (2) with cyclopentene (86) in the presence of AlCl_3_ and DCE at 0 °C for 24 h afforded 4-cyclopentyl-1,1′-biphenyl (87).^107^ Additionally, using trichloromethane (CHCl_3_) instead of DCE at 20 °C for 10 h led to formation of 4,4′-dicyclopentyl-1,1′-biphenyl (88) (Scheme 37).^108^

#### 3.1.2 Friedel–Crafts acylation

The Friedel–Crafts acylation is the reaction of acid anhydrides or acyl chlorides with aromatic compounds in the presence of Lewis acid catalyst for the synthesis of methyl aryl ketone, which are used in pharmaceuticals and fine chemical such as ibuprofen, naproxen, dyes, agrochemicals, and fragrances.^109,110^

##### 3.1.2.1 Reaction of biphenyl with acid anhydride

**Scheme 38:**
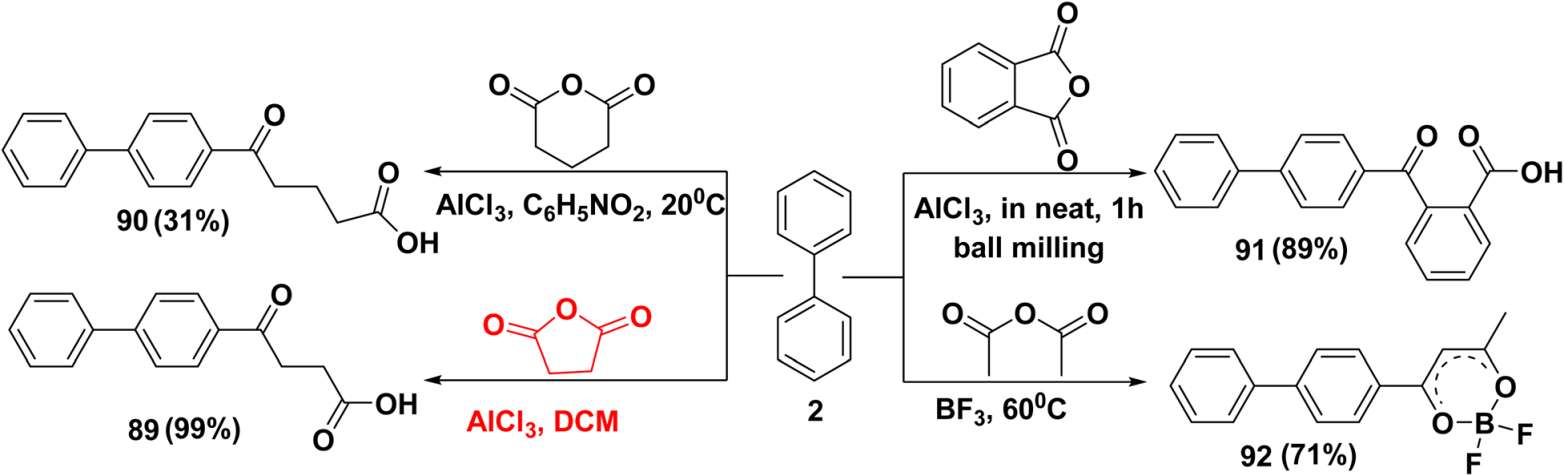
Friedel–Crafts acylation of biphenyl with anhydrides.

4-Phenylbenzoyl-propionic acid (89) can be easily prepared *via* Friedel–Crafts acylation of biphenyl (2) and succinic anhydride in the presence of AlCl_3_ and dichloromethane (DCM).^111^ While, treatment of biphenyl (2) with dihydro-2*H*-pyran-2,6(3*H*)-dione in the presence of AlCl_3_ and nitrobenzene (C_6_H_5_NO_2_) afforded biphenyl-4-yl-5-oxopentanoic acid (90).^112^ Further, biphenyl-4-carbonyl-benzoic acid (91) was synthesized *through* free-solvent mechanochemical ball milling method *via* reaction of biphenyl (2) with phthalic anhydride in the presence of AlCl_3_.^113^ Whereas, gaseous boron trifluoride (BF_3_) was passed *through* a homogenous mixture of biphenyl (2) and acetic anhydride to synthesize biphenyl-4-yl-2,2-difluoro-6-methyl-dioxaborinine (92) (Scheme 38).^114^

##### 3.1.2.2 Reaction of biphenyl with acid chloride

**Scheme 39:**
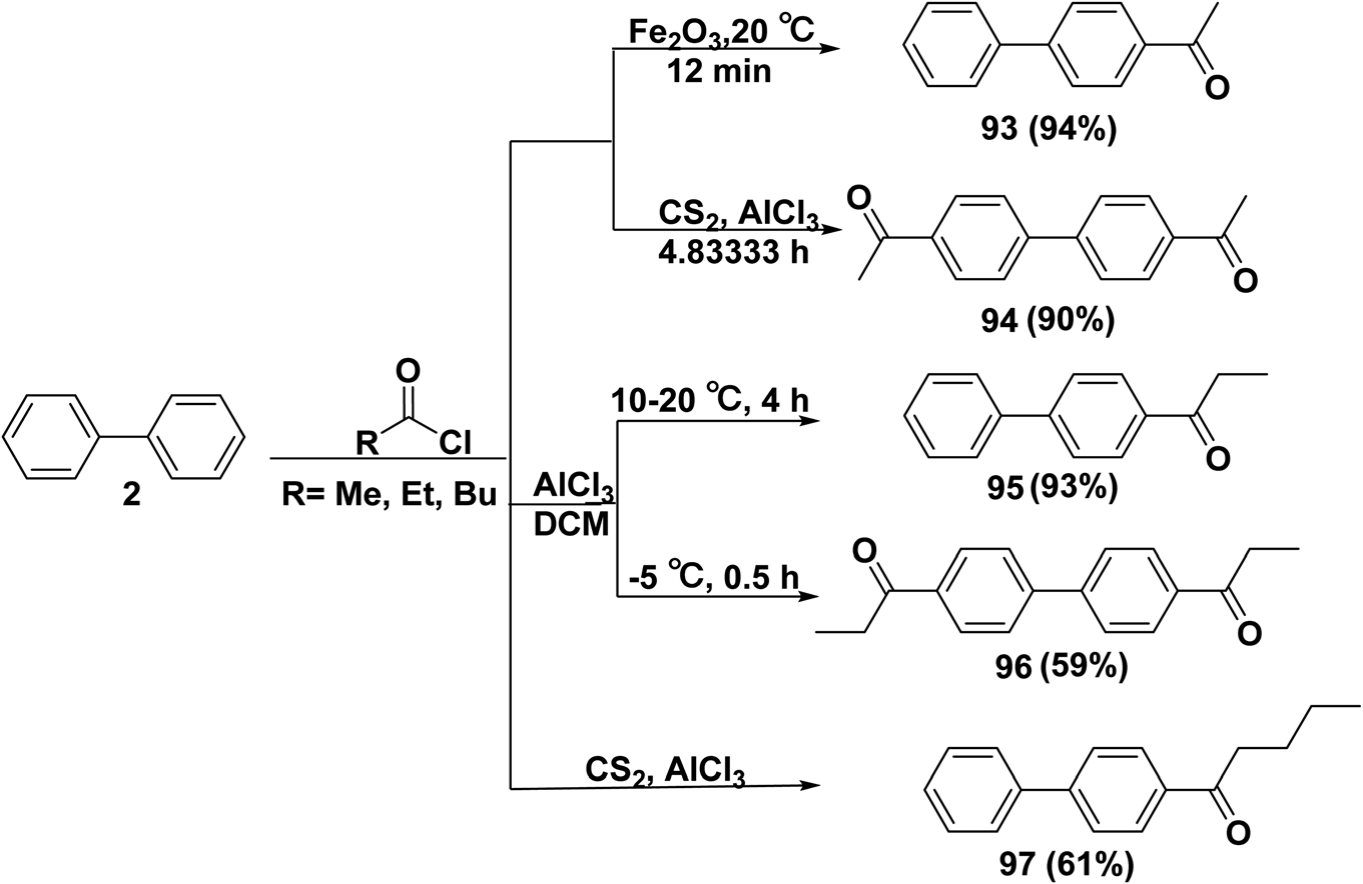
Reaction of biphenyl with acyl chloride derivatives.

Reaction of biphenyl (2) with a mixture of activated iron(iii)oxide (Fe_2_O_3_) and acetyl chloride, 1-([1,1′-biphenyl]-4-yl)ethan-1-one (93) was produced.^110^ In contrast, acetyl chloride in dried carbon disulfide (CS_2_) was added drop by drop with stirring into a suspension solution containing biphenyl (2), anhydrous AlCl_3_, and dried (CS_2_) to obtain 1,1′-([1,1′-biphenyl]-4,4′-diyl)bis(ethan-1-one) (94).^115^ Additionally, biphenyl (2) was refluxed with propionyl chloride for 4 h in DCM containing a catalytic amount of AlCl_3_ to furnish 1-([1,1′-biphenyl]-4-yl) propan-1-one (95),^116^ whereas 1,1′-([1,1′-biphenyl]-4,4′-diyl)bis(propan-1-one) (96) was produced by refluxing biphenyl (2) with propionyl chloride for 2 h at −5 °C.^117^ Under anhydrous conditions, a stirred mixture of CS_2_, biphenyl (2), and anhydrous AlCl_3_ was treated with pentanoyl chloride to afford 1,1′-biphenyl-4-pentan-1-one (97) (Scheme 39).^118^

**Scheme 40:**
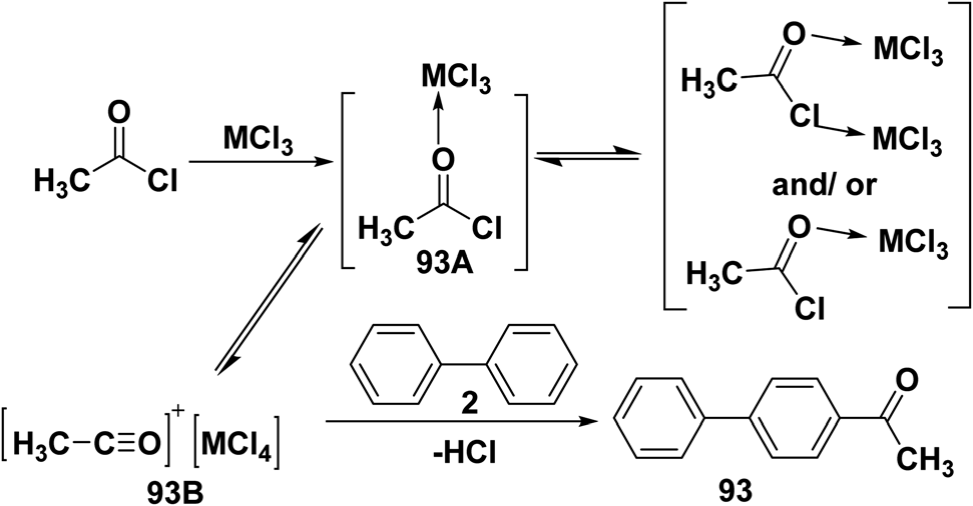
Friedel–Crafts acylation mechanism.

The proposed mechanism for Friedel–Crafts acylation shows that the reaction began with the Lewis acids MCl_3_ (M = Fe or Al) activating the acetyl chloride to produce an adduct of acyl chloride with MCl_3_ (93A). This complex is quickly equilibrated with the acylium ion [CH_3_CO]^+^[MCl_4_] (93B), which then attacks biphenyl (2) to produce biphenyl alkyl ketone 93 (Scheme 40).^102^

##### 3.1.2.3 Reaction of biphenyl with ketene

**Scheme 41:**
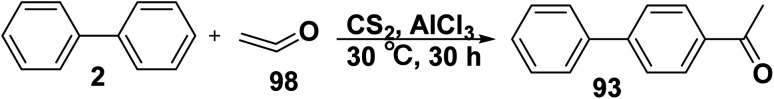
Reaction of biphenyl (2) with ethenone.

1-([1,1′-Biphenyl]-4-yl)ethan-1-one (93) was synthesized *via* Friedel–Crafts reaction by stirring ethenone (98) with a solution of biphenyl (2) in CS_2_, then adding AlCl_3_ (Scheme 41).^119^

##### 3.1.2.4 Reaction of biphenyl with oxalyl chloride

**Scheme 42:**
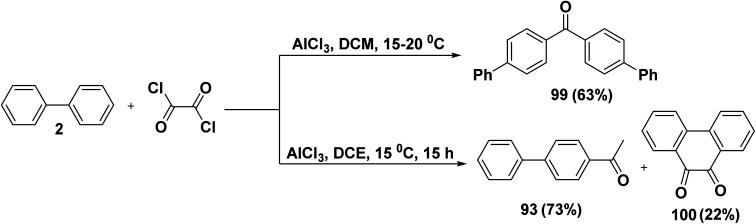
Acylation of biphenyl with (ClCO)_2_.

A facile synthesis of 4,4′-biphenyl ketone 99 was accomplished *via* the reaction of biphenyl (2) with oxalyl chloride (ClCO)_2_ in the presence of AlCl_3_ and DCM.^120^ While, the reaction of biphenyl (2) with oxalyl chloride under the influence of AlCl_3_ in DCE produced 1-([1,1′-biphenyl]-4-yl)ethan-1-one (93) and 1,2-di([1,1′-biphenyl]-4-yl)ethane-1,2-dione (100) (Scheme 42).^121^

### 3.2 Isopropylation reaction of biphenyl

**Scheme 43:**
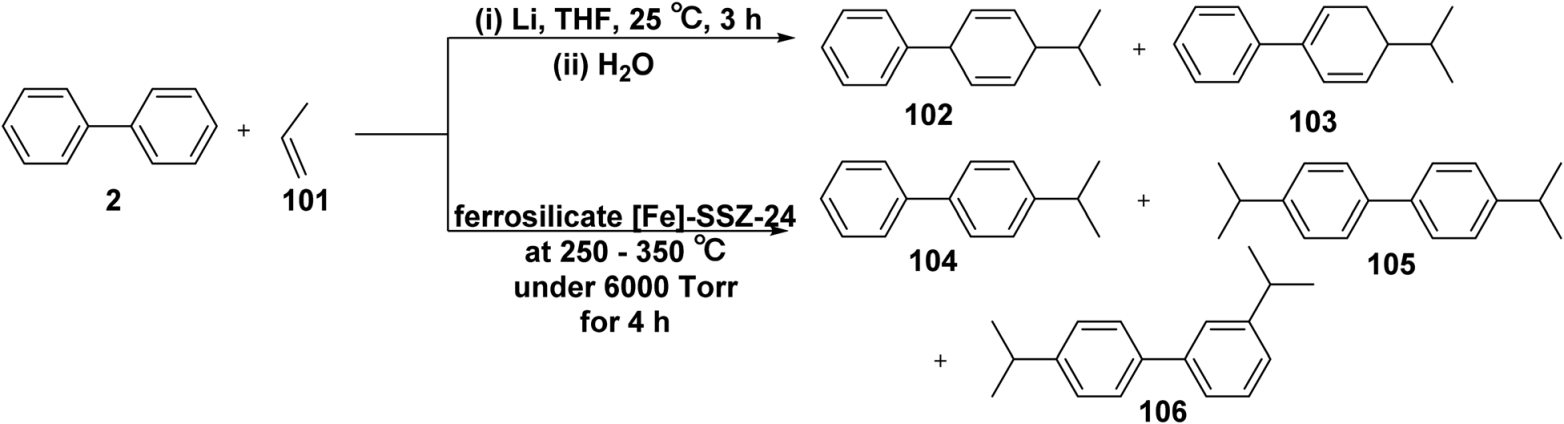
Reaction of biphenyl with propene.

Reaction of biphenyl with propene (101) in the presence of lithium metal (Li), and THF, followed by hydrolysis afforded a mixture of 4-isopropyl-1,4-dihydro-1,1′-biphenyl (102) and 4-isopropyl-3,4-dihydro-1,1′-biphenyl (103).^122^ Furthermore, a mixture of 4-isopropyl-1,1′-biphenyl (104), 4,4′-diisopropyl-biphenyl (105), and 3,4′-diisopropyl-1,1′-biphenyl (106) were produced *through* the reaction of biphenyl (2) with propene (101) in the presence of [Fe]-SSZ-24 catalyst (Scheme 43).^123^

**Scheme 44:**
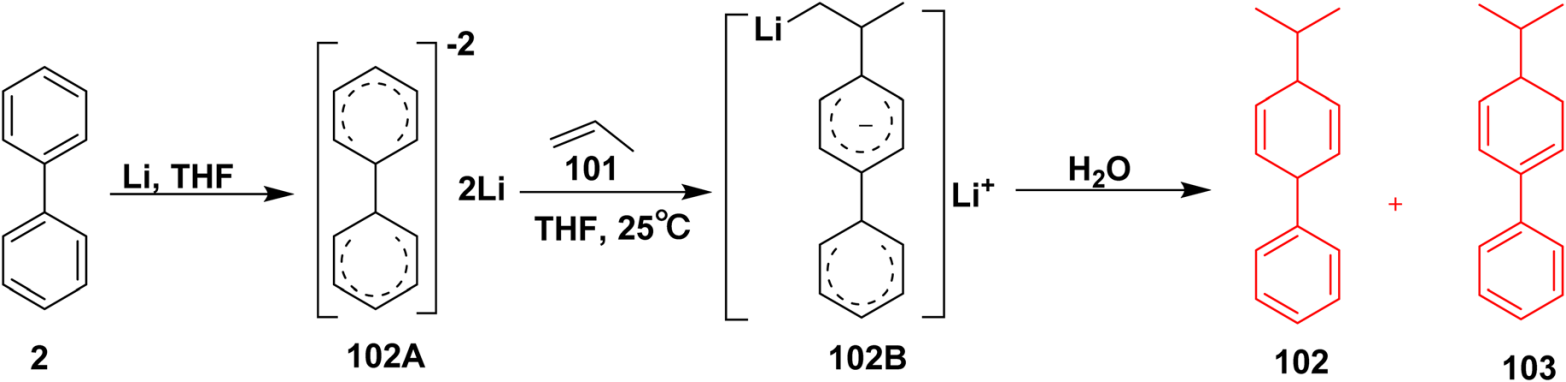
A postulated mechanism for biphenyl isopropyl synthesis.

The proposed mechanism for the synthesis of isopropyl biphenyl derivatives starts with *in situ* formation of carbolithiation step in which the biphenyl (2) is doubly reduced in a mixture of lithium metal (Li) and THF affording biphenyl dianion intermediate 102A. Then, biphenyl dianion 102A reacted with propene (101) in THF, followed by addition step to yield intermediate 102B, finally the hydrolysis step is achieved to furnish 4-isopropyl-1,4-dihydro-1,1′-biphenyl (102) and 4-isopropyl-3,4-dihydro-1,1′-biphenyl (103) (Scheme 44).^122^

### 3.3 Halogenation reactions

#### 3.3.1 Chlorination reactions

**Scheme 45:**
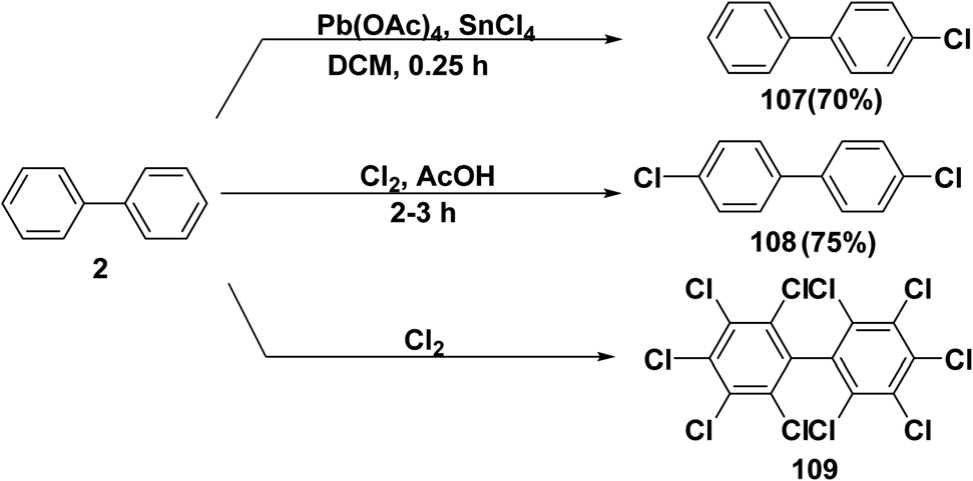
Chlorination of biphenyl.

Polychlorinated biphenyls (PCBs) are a class of chlorinated organic compounds which were widely used in commercial and industrial applications. PCBs have many industrial applications including heat transfer, electrical, lubricants, and hydraulic equipment; as plastics, rubber products, coating, adhesive, capacitors, and plasticizers in paints; carbonless copy paper, and dyes.^124^ A solution of biphenyl (2) and anhydrous tin(iv)chloride (SnCl_4_) in DCM was stirred, then lead tetraacetate Pd(OAc)_4_ was added in portion to produce 4-chloro-1,1′-biphenyl (107).^125^ Whereby, dichlorobiphenyl 108 was synthesized *through* passing chlorine gas (Cl_2_) to a solution of biphenyl (2) in glacial acetic acid (AcOH).^126^ On the other hands, biphenyl (2) was exposed to chlorine gas to obtain poly chlorinated biphenyl derivative 109 (Scheme 45).^127^

#### 3.3.2 Bromination reactions

**Scheme 46:**
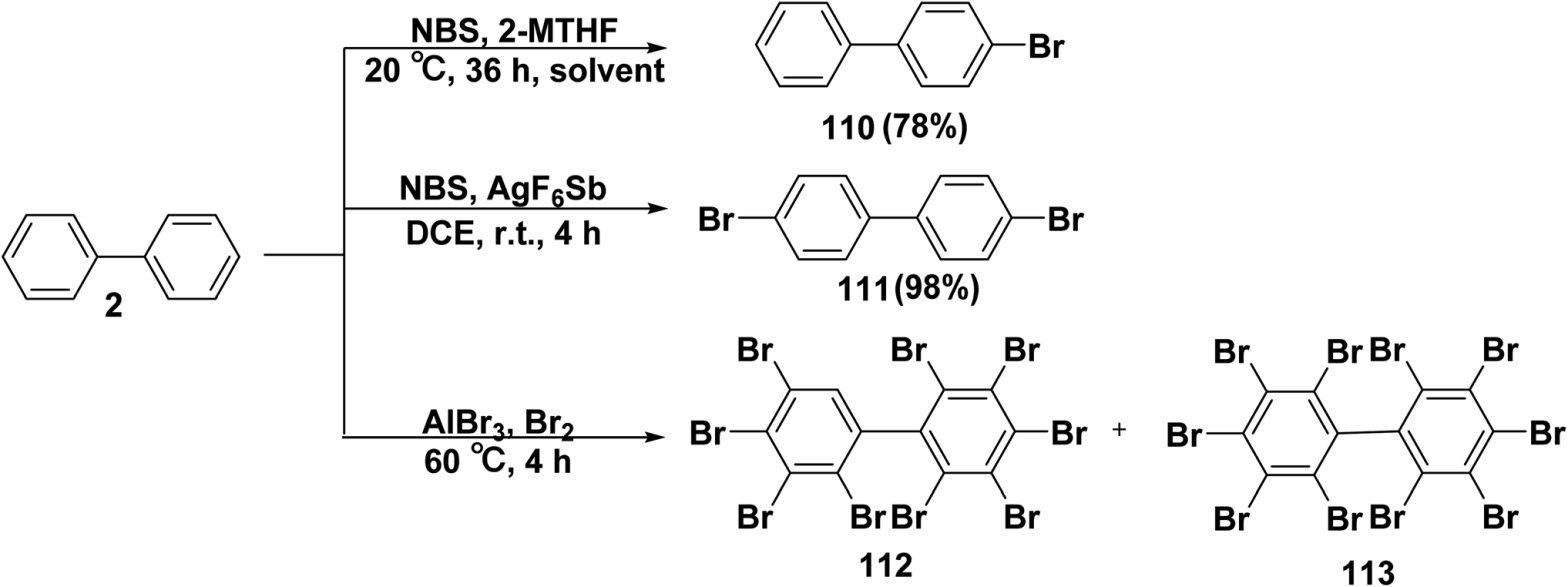
Bromination of biphenyl.

On the area of research, the study of bromine containing molecules has been found to have an extensive range of biological activities, including those that are antiparasitic, antibacterial, antiviral, antioxidant, anti-inflammatory, and anticancer. Furthermore, mixtures of polybrominated biphenyls (PBBs) have been employed as additives in polymers as well as flame retardants in the textile and electronic sectors.^128,129^ Stirring of biphenyl (2) with *N*-bromosuccinimide (NBS) in 2-methyltetrahydrofuran (2-MTHF) as solvent furnished mono bromobiphenyl 110.^130^ Whereas, the bromination of compound 2 by NBS in the presence of AgSbF_6_ and Trip-SMe in DCE furnished dibromo-biphenyl 111.^131^ Further, bromine gas (Br_2_) was mixed with biphenyl (2), cooled in an ice bath, and AlBr_3_ was added dropwise under the synthesis of HBr to afford PBBs 112, 113 (Scheme 46).^129^

#### 3.3.3 Iodination reactions

**Scheme 47:**
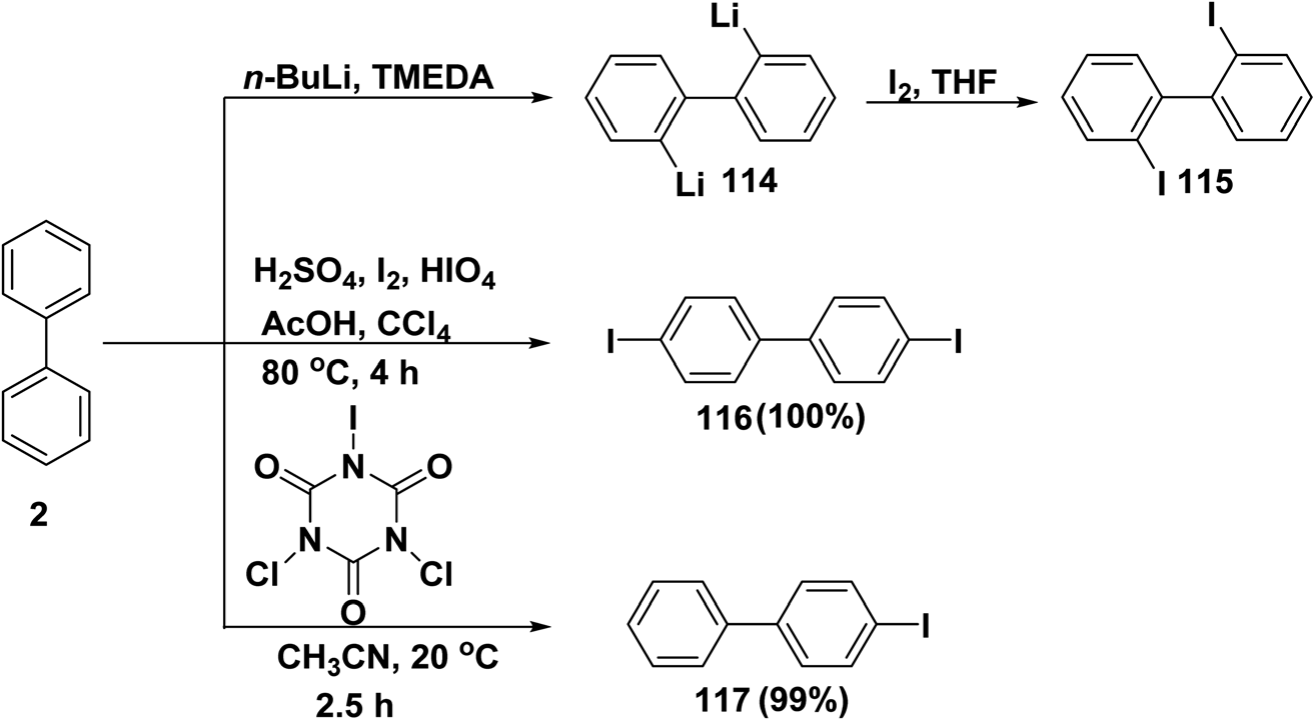
Different iodinated derivatives of biphenyl.

Iodo-organic molecules are extensible and useful synthetic intermediates which have numerous applications in pharmacology, medicine, and polymer science.^132^ Lithiation of biphenyl (2) with *n*-butyllithium (*n*-BuLi) in the presence of tetra methyl ethylenediamine (TMEDA) led to formation of 2,2′-dilithiobiphenyl (114). Then, dilithiobiphenyl 114 reacted with iodine gas (I_2_) in THF to give 2,2′-diiodobiphenyl (115).^133,134^ On the other hand, a mixture of biphenyl (2), water, AcOH, concentrated sulfuric acid (H_2_SO_4_), I_2_, periodic acid (HIO_4_), and carbon tetrachloride (CCl_4_) was heated for 4 h to produce diiodobiphenyl 116.^135^ Furthermore, stirring of biphenyl (2) with dichloroiodoisocyanuric acid (DCICA) in CH_3_CN afforded 4-iodo-1,1′-biphenyl (117) (Scheme 47).^132^

### 3.4 Chloromethylation reaction of biphenyl

**Scheme 48:**
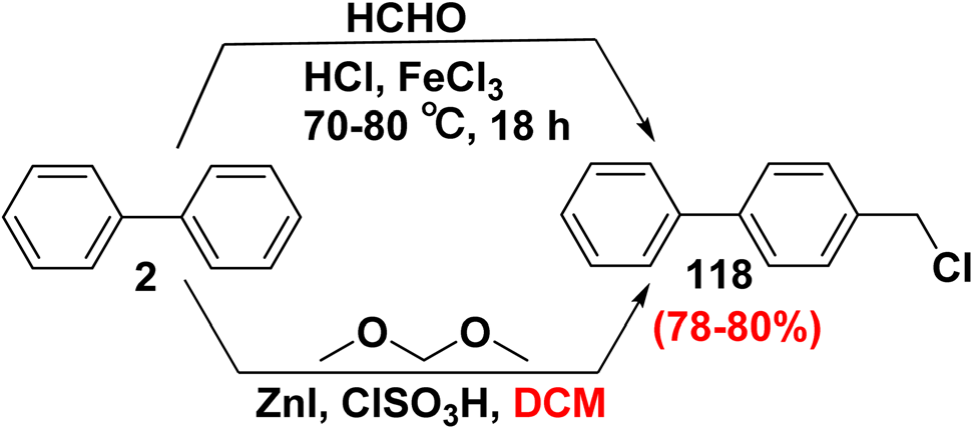
Chloromethylation of biphenyl.

Chloromethyl-substituted aromatic compounds are significant intermediates due to their easy transformation into a wide range of fine or special chemicals, pharmaceuticals, and polymers.^136^ The chloromethylation reaction of biphenyl (2) with formalin (HCHO) in the presence of HCl and FeCl_3_ produced 4-(chloromethyl)-1,1′-biphenyl (118) in 80% yield.^137^ Whereas, 4-(chloromethyl)-1,1′-biphenyl (118) was afforded in 78% yield *through* the treatment of biphenyl (2) with dimethoxymethane (CH_2_(OCH_3_)_2_) and chlorosulfonic acid (ClSO_3_H) in the presence of a catalytic amount of zinc iodide (ZnI_2_) in DCM (Scheme 48).^136^

**Scheme 49:**
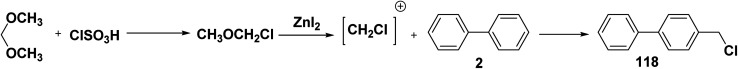
Possible mechanism of chloromethylation of biphenyl.

Scheme 49 depicts a probable mechanism for the formation of chloromethyl-1,1′-biphenyl 118. The first step involves treatment of CH_2_(OCH_3_)_2_ with ClSO_3_H affords methyl chloromethyl ether. Then, ZnI_2_ promotes the production of the chloromethyl cation ([ClCH_2_]^+^). Finally, a chloromethylated derivative 118 is obtained by the electrophilic substitution reaction of biphenyl 2 with [ClCH_2_]^+^ (Scheme 49).^136^

### 3.5 Reduction of biphenyl

**Scheme 50:**
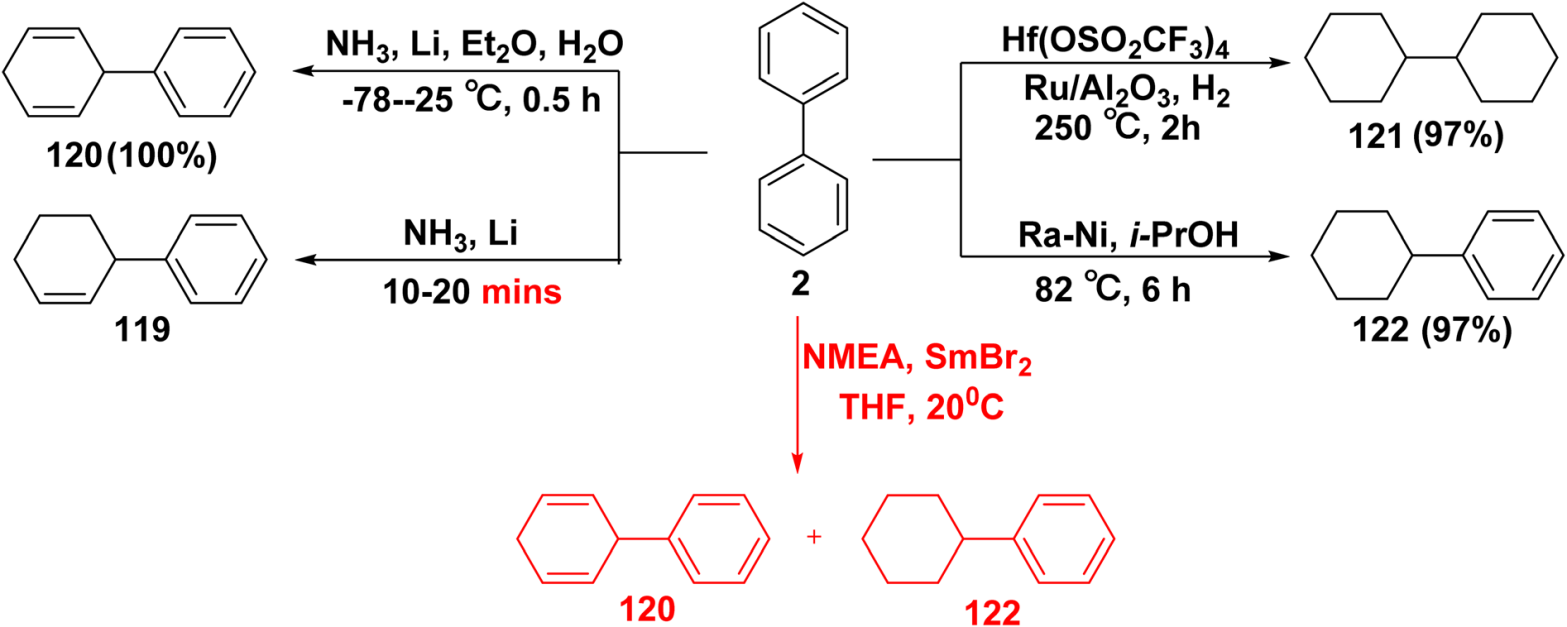
Reduction of biphenyl.

Reduction of biphenyl (2) with reducing lithium metal and ammonia afforded tetrahydro-1,1′-biphenyl 119.^138^ Analogously, 3-phenyl-1,4-cyclohexadiene (120) was synthesized when liquid ammonia was added to Et_2_O containing biphenyl (2), followed by the addition of lithium metal.^139^ While, the treatment of biphenyl (2) with a noble metal catalyst (*e.g.*, Ru/C) in the presence of Lewis acid (Hf(OTF)_4_) and octane (OCT) furnished 1,1′-bi(cyclohexane) (121).^140^ Whereas, nickel catalyzed hydrogenation of biphenyl (2) in the presence of isopropyl alcohol (i-PrOH) as a hydrogen donor afforded cyclohexylbenzene (122).^141^ Whereby, reduction of biphenyl 2 with a combination of samarium dibromide (SmBr_2_) and *N*-methylethanolamine (NMEA) as a chelating ligand furnished a mixture of 120 and 122 (Scheme 50).^142^

### 3.6 Reaction of biphenyl with triazine derivatives

**Scheme 51:**
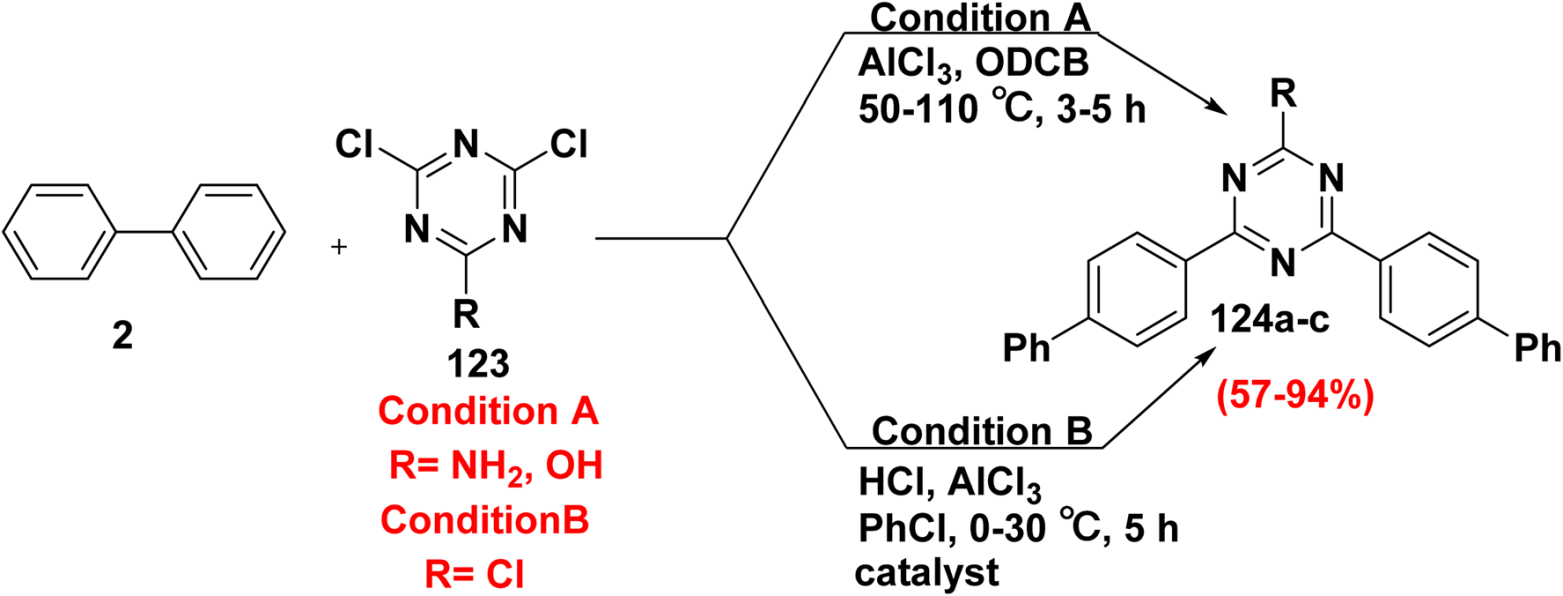
Formation of biphenyl triazine scaffolds.

4,6-Di([1,1′-biphenyl]-4-yl)-1,3,5-triazin-2-amine (124a) and 4,6-di([1,1′-biphenyl]-4-yl)-1,3,5-triazin-2-ol (124b) were synthesized by heating 4,6-dichloro-1,3,5-triazin derivatives 123 with a solution of *o*-dichlorobenzene (ODCB) with AlCl_3_, followed by adding a biphenyl solution of ODCB.^143,144^ In addition, biphenyl (2) was allowed to react with 2,4,6-trichloro-1,3,5-triazine (123) in the presence of hydrogen chloride (HCl) and AlCl_3_ in chlorobenzene yielded trisubstituted-6-chloro-1,3,5-triazine 124a–c (Scheme 51).^145^

### 3.7 Rection of biphenyl with acid

**Scheme 52:**
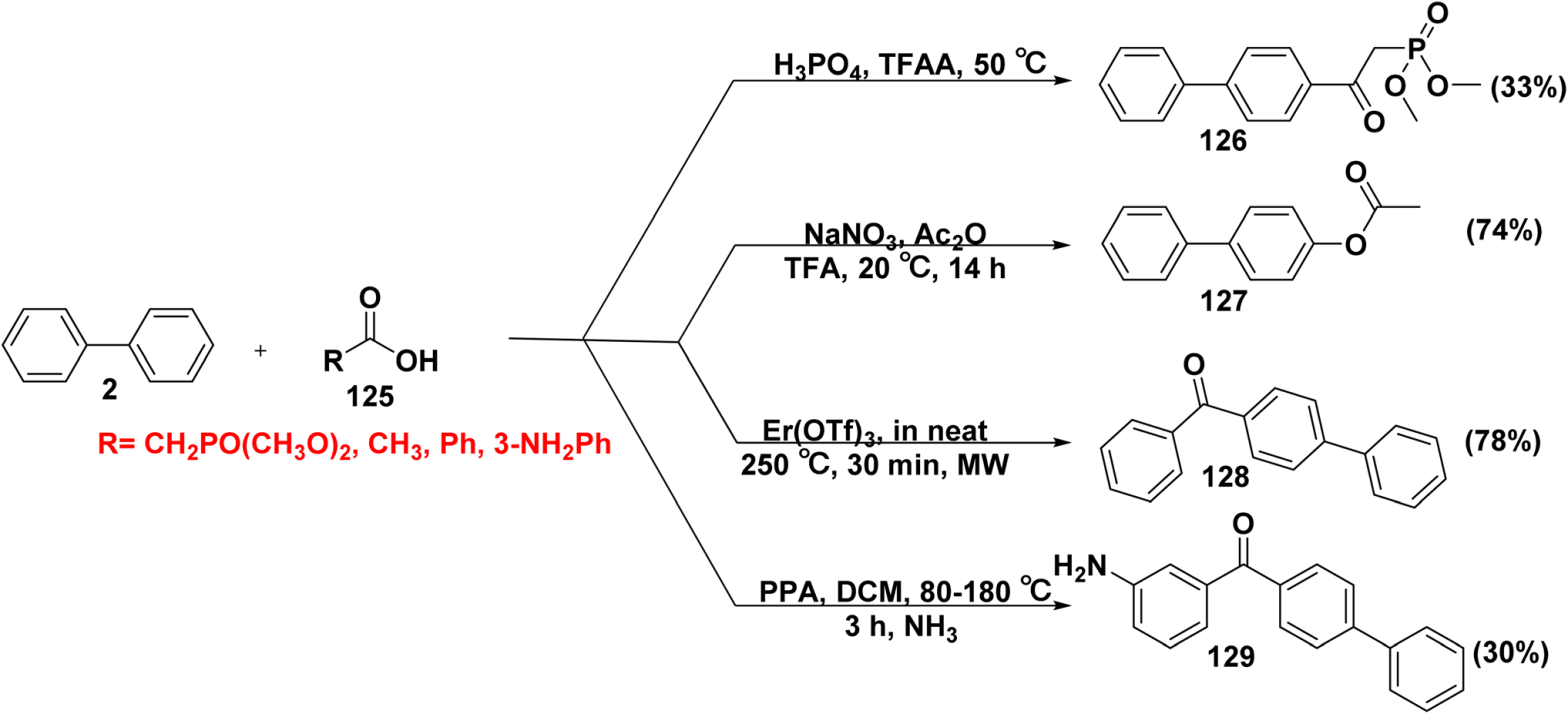
Acetoxylation of biphenyl with various acids.

Acetylation of arenes is a significant reaction and still essential need in chemistry. Oxygenated arenes, for example aryl acetates, are valuable synthetic precursors for pharmaceuticals, natural products, and functional materials.^146^ Luke *et al.*^147^ investigated the use of β-aryl-β-ketophosphonates as bone anabolic agent, thyroid receptor ligands, and tumor cell profiler inhibitors. Dimethyl(2-([biphenyl]-4-yl)-2-oxoethyl)phosphonate 126 was synthesized by stirring biphenyl (2) with trifluoroacetic anhydride (TFAA), which was then treated with dimethylphosphonoacetic acid (125) and phosphoric acid (H_3_PO_4_). Under purified nitrogen condition, a mixture of sodium nitrate (NaNO_3_) in trifluoroacetic acid (TFA), ACOH, and Ac_2_O was mixed with a solution of biphenyl (2) afforded [1,1′-biphenyl]acetate (127).^146^ Erbium(iii) triflate (Er(OTf)_3_) catalyzed Friedel–Crafts acylation of biphenyl (2) using acylating agent, such as benzoic acid (125) under microwave irradiation furnished [1,1′-biphenyl]-4-yl(phenyl)methanone (128).^148^ Furthermore, (3-amino-phenyl)-biphenyl-4-yl-methanone (129) was obtained by adding 3-aminobenzoic acid (125) and polyphosphoric acid (PPA) to biphenyl (2) (Scheme 52).^149^

### 3.8 Rection of biphenyl with diazonium salt

**Scheme 53:**
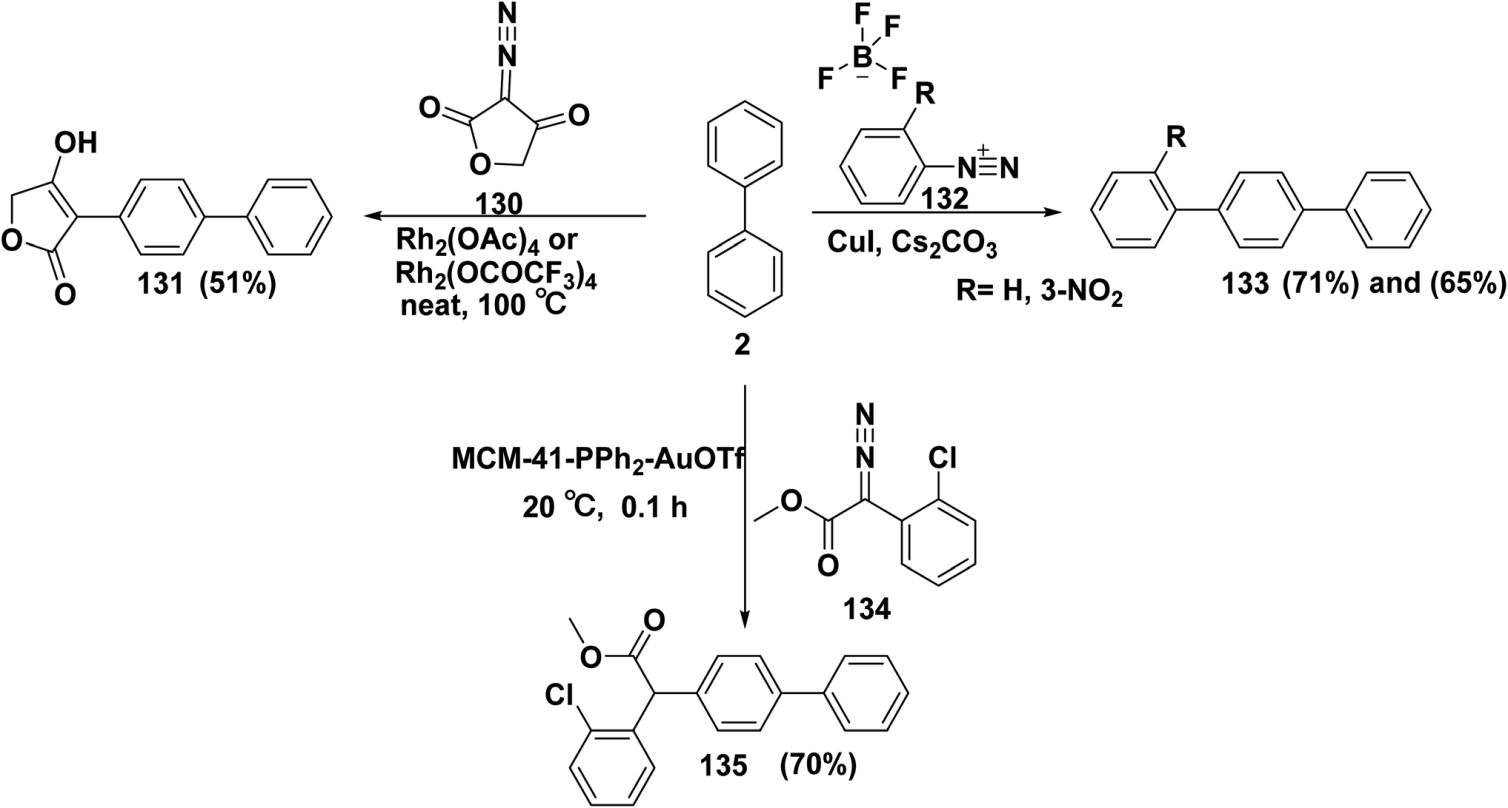
Reaction of biphenyl with diazonium salts.

Manchoju *et al.*^150^ emphasized that several tetronic acid derivatives displayed a wide range of pharmacological activities, including acaricidal, insecticidal, HIV-I protease inhibitory, anti-inflammatory, antineoplastic, and cyclooxygenase inhibitory activity. Under conventional heating, synthesis of 1,1′-bipheny-4-hydroxyfuran-2(5*H*)-one 131 was achieved in excellent yield (51%) by reaction of biphenyl (2) and 3-diazofuran-2,4-dione 130. Gomberg–Bachmann–Hey reactions of benzendiazonium tetrafluoroborate derivatives 132 with biphenyl (2) in the presence of copper catalyst and cesium carbonate (Cs_2_CO_3_) in CH_3_CN afforded terphenyl derivatives 133.^151^ In the same context, heterogeneous gold(i)-catalyzed reaction of biphenyl (2) with 2-chlorophenyl-2-diazoacetate 134 afforded methyl 2-([biphenyl]-4-yl-2-chlorophenyl)acetate 135 (Scheme 53).^152^

**Scheme 54:**
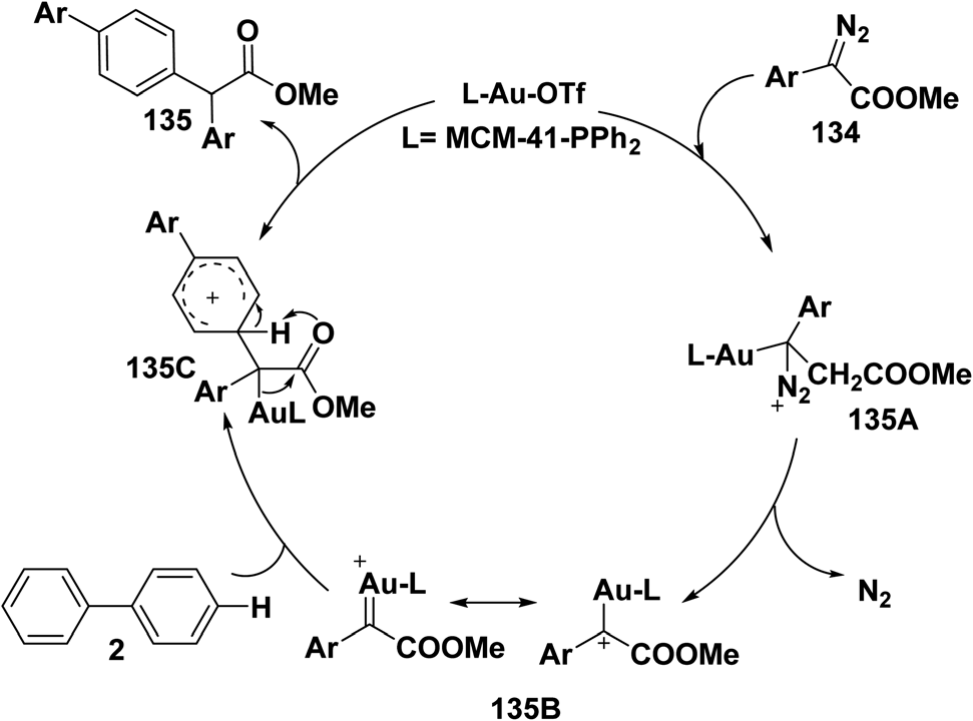
Heterogeneous gold(i)-catalytic cycle mechanism.

Heterogeneous gold(i)-catalyzed mechanism of inactivated arenes is passed *via* sequence steps. Initially, for the synthesis of diazonium ion intermediate 135A, the cationic gold MCM-41–PPh_2_–AuOTf exchanges ligands with the diazo ester 134. Then, intermediate 135A collapses to produce the MCM-41-bound gold(i)carbene intermediate 135B, followed by combination between the nucleophilic arene 2 and the electrophilic gold(i)carbene intermediate 135B, the gold ionic intermediate 135C is produced. Last, intermediate 135C goes *through* 1,4-*H* shift and deauration to afford product 135 and MCM-41–PPh_2_–AuOTf complex is regenerated (Scheme 54).^152^

### 3.9 Reaction of biphenyl with isothiazol-3(2*H*)-one-1,1-dioxide derivatives

**Scheme 55:**
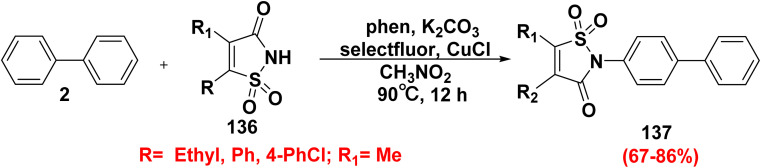
Formation of biphenyl saccharin derivatives.

Copper-catalyzed reaction of biphenyl (2) with saccharin derivatives 136 in the presence of 1,10-phenanthroline (phen), K_2_CO_3_, and selectfluor as oxidant in refluxing CH_3_CN furnished biphenyl saccharin derivatives 137 (Scheme 55).^153^

### 3.10 Nitration of biphenyl

**Scheme 56:**
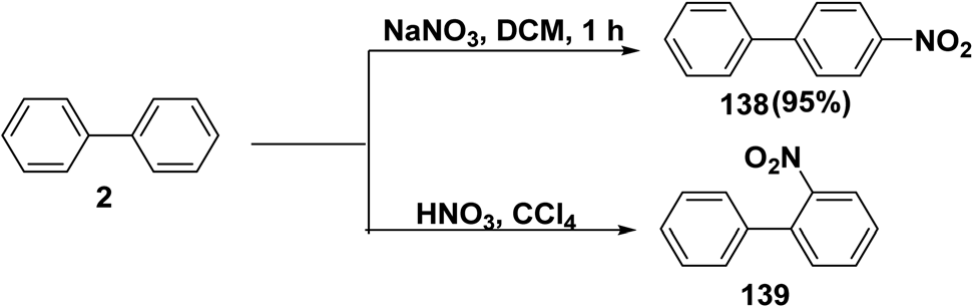
Nitration of biphenyl.

Several reagents could be used to perform the nitration of aromatic compounds. The nitro aromatic compounds are extensively used in the manufacture of perfumes, pharmaceuticals, explosives, plastics, and dyes. Nitration of biphenyl (2) gave various oriented nitrated products 138 and 139 depending upon the nature of conditions and reagents applied on the reaction, as the nitration of biphenyl (2) was employed by refluxing NaNO_3_with SO_3_H-functionalized magnetic core/shell nano catalyst in DCM furnished 4-nitro-1,1′-biphenyl (138).^154^ While, treatment of a biphenyl with nitric acid (HNO_3_) in CCl_4_ solvent afforded 2-nitro-biphenyl 139 (Scheme 56).^155^

### 3.11 Reaction of biphenyl with fused compounds

**Scheme 57:**
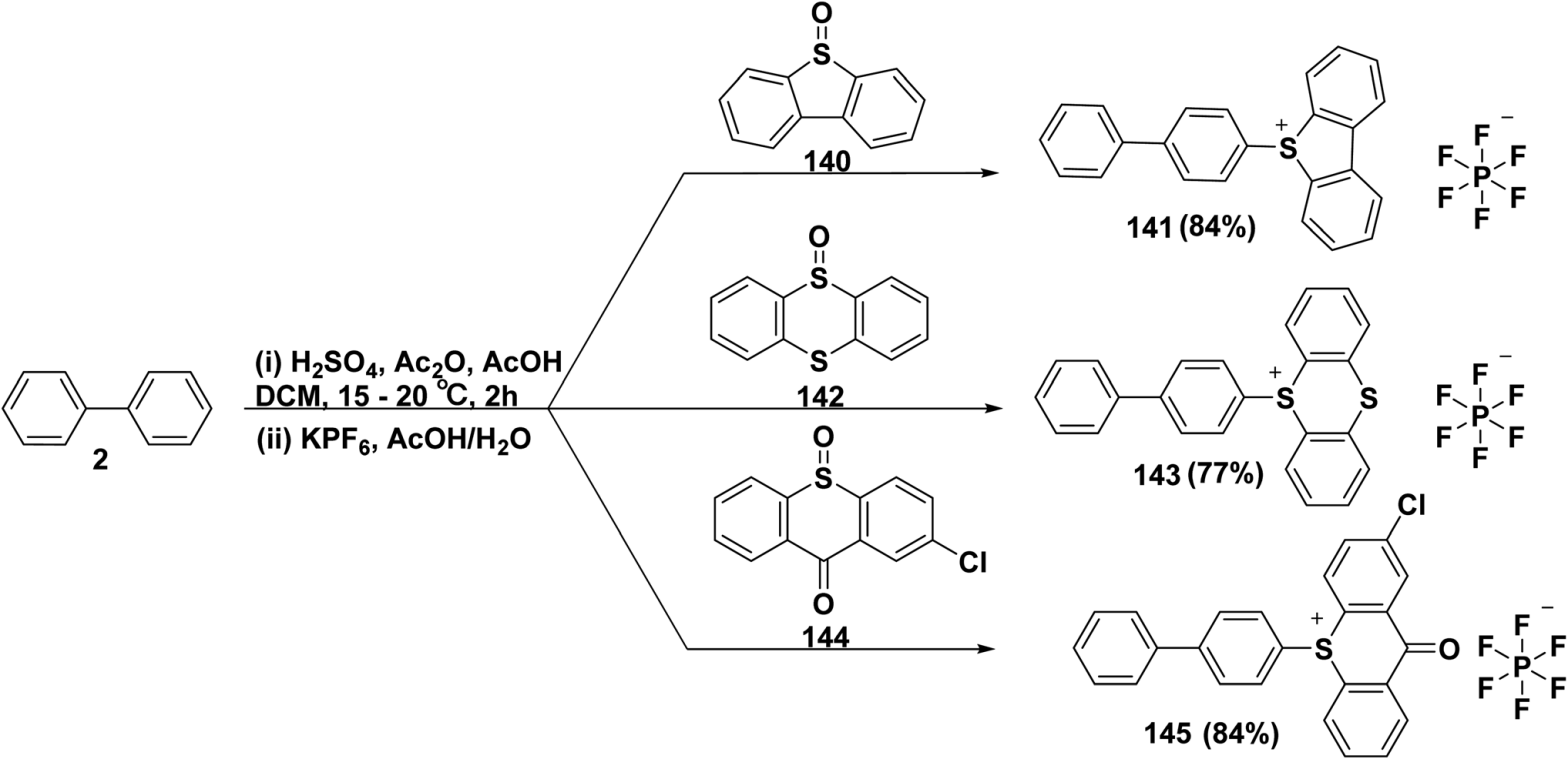
Reaction of biphenyl with fused compounds.

A class of fused ring, such as thioxanthone, dibenzothiophene, and thianthrene are applied as cationic photo-initiator, specifically for usage in surface coating applications, for example, varnishes and inks. 5-([1,1′-biphenyl]-4-yl)-5*H*-dibenzo[*b*,*d*]thiophen-5-ium-hexafluorophosphate(v) (141) was obtained in two steps (Scheme 57) starting of mixing biphenyl (2) and dibenzo[*b*,*d*]thiophene 5-oxide (140) with AcOH and Ac_2_O in DCM, followed by adding sulfuric acid (H_2_SO_4_) dropwise. In the second stage, the intermediate product was neutralized by potassium hexafluorophosphate (KPF_6_) in water. Under the same conditions, compounds 143 and 145 were produced by reacting biphenyl (2) with thianthrene 5-oxide (142) and 2-chloro-9*H*-thioxanthen-9-one 10-oxide (144), respectively (Scheme 57).^156^

### 3.12 Reaction of biphenyl with phenol

**Scheme 58:**
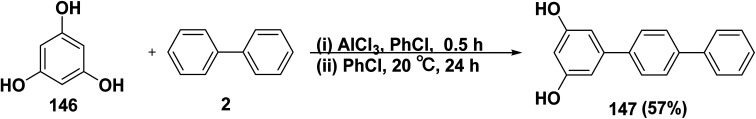
Reaction of biphenyl with phenol.

Terphenyl-3,5-diol 147 was synthesized *through* stirring phloroglucinol (146) with AlCl_3_ in chlorobenzene (PhCl), followed by adding biphenyl (Scheme 58).^157^

### 3.13 Rection of biphenyl with sulfonanilide derivatives

**Scheme 59:**
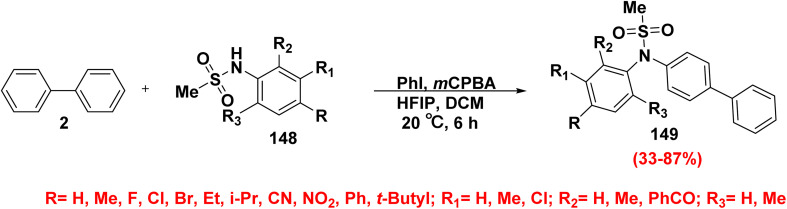
Synthesis of biphenyl sulfonanilide derivatives.

The development of new methods for C–N bond synthesis is of great importance due to the prevalence nitrogenous compounds in numerous synthetic intermediates, natural products, pharmaceutical agents, and biologically active molecules. Sulfonanilides are a crucial class of synthetic scaffolds used in organic synthesis due to their potent electron-withdrawing effect of the connected sulfonyl group, in addition to, sulfonanilides have a special reactivity toward hypervalent iodine(iii). Biphenyl sulfonanilide derivatives 149 were produced in wide range yields (33–87%) by adding *meta*-chloroperbenzoic acid (*m*CPBA) to a stirred solution of sulfonamide derivatives 148, biphenyl 2, and iodobenzene (PhI) in a mixture of hexafluoroisopropanol (HFIP) and DCM (Scheme 59).^158^

### 3.14 Amination reaction

**Scheme 60:**
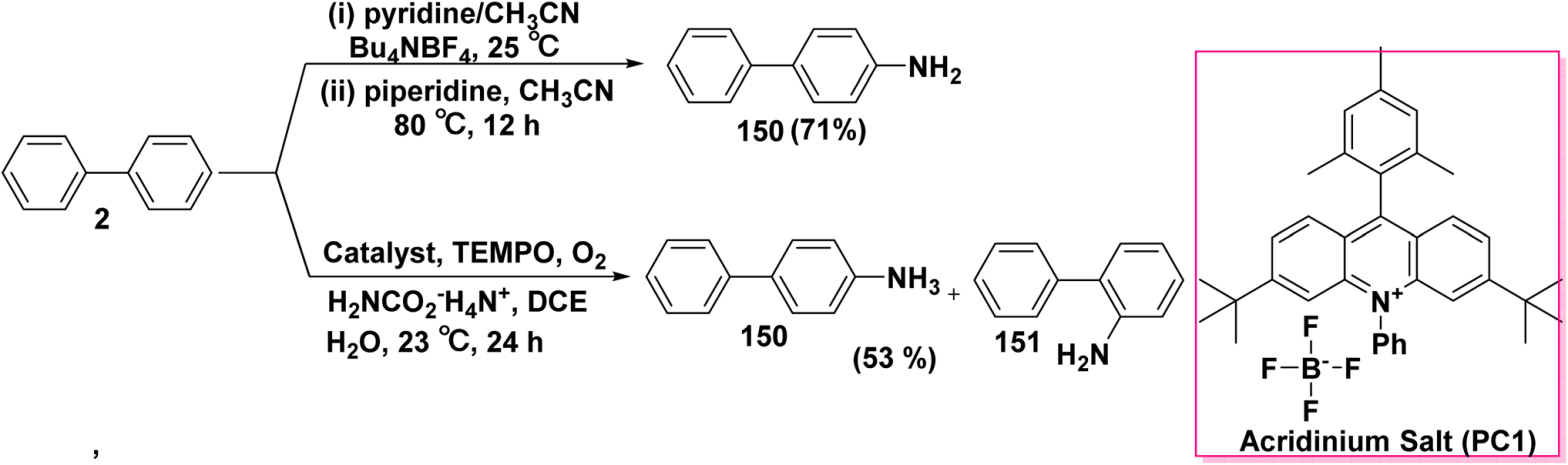
Reaction of biphenyl with different amines.

Primary aromatic amines are important building blocks for the formation of biologically active pharmaceutical and agrochemical chemicals, as well as organic functional materials like dyes and pigments. Electrochemical oxidation of biphenyl (2) in a solution of tetrabutylammonium tetrafluoroborate (Bu_4_NBF_4_) and a mixture of CH_3_CN and pyridine followed by adding piperidine afforded aminobiphenyl 150 (Scheme 60).^159^ Analogously, under an oxygen atmosphere conditions, the reaction of ammonium carbamate (H_2_NCO_2_^−^H_4_N^+^) as a nucleophile with biphenyl (2) as the arene coupling partner in the presence of a catalytic amount of mesityl acridinium salt, 2,2,6,6-tetramethylpiperidine-1-oxyl (TEMPO), and a mixture of DCE and H_2_O afforded *para* and *ortho* aminobiphenyl products 150 and 151 respectively (Scheme 60).^160^

**Scheme 61:**
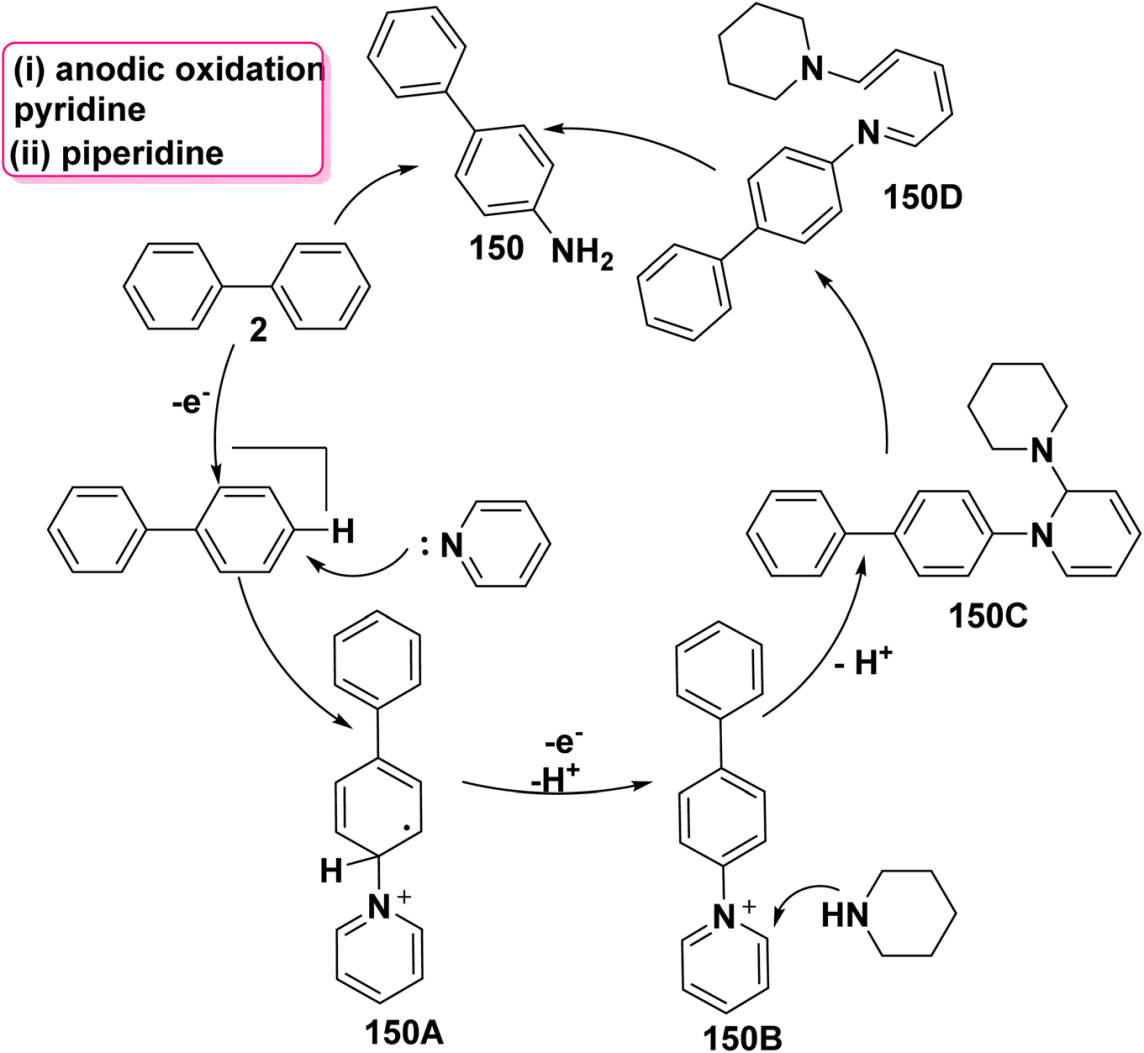
The possible mechanism for aminobiphenyl synthesis.

A proposed mechanism for 4-aminobiphenyl (150) synthesis involves the nucleophilic attack of pyridine on biphenyl (2), followed by one-electron oxidation to produce the intermediate ion 150A which is underwent aromatization process to yield *N*-arylpyridinium ion intermediate 150B. Then, piperidine is added to the 2-position of the *N*-arylpyridinium ion 150B, accompanied by ring opening and imine hydrolysis (Scheme 61).^159^

**Scheme 62:**
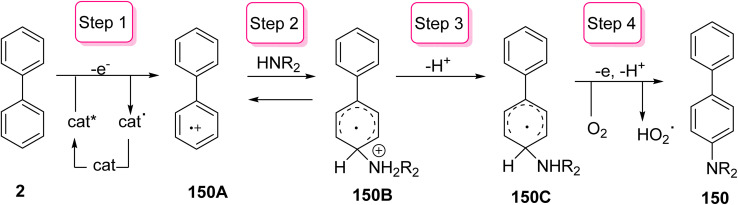
A postulated mechanism for the formation of aminobiphenyl.

On the other hand, the proposed mechanism for the formation of aminobiphenyl 150 involves photoinduced electron transfer (PET) of biphenyl 2 in the presence of acridinium salt as a catalyst to an excited state photoredox radical (cat*) and biphenyl cation radical 150A, which reacted with amine derivatives to yield distonic cation radical 150B. Then, the deprotonation reaction of 150B furnished the radical intermediate 150C, which led to production of aminobiphenyl 150 by oxidative aromatization (Scheme 62).^160^

### 3.15 Reaction of bromo-biphenyl with boronic acid

**Scheme 63:**
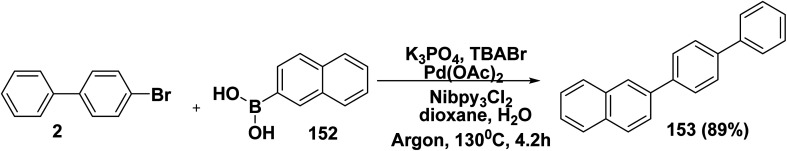
Synthesis of biphenyl naphthalene derivative.

Kashid and coworkers^161^ reported the synthesis of biphenyl-4-yl-naphthalene 153*via* the treatment of bromo-biphenyl 2 with naphthalen-2-yl-boronic acid (152) in the presence of tetrabutylammonium bromide (TBABr), K_3_PO_4_, Pd(OAc)_2_ and Ni complex in a mixture of dioxane and water (Scheme 63).

### 3.16 Reaction of biphenyl with bis(pinacol)diborane

**Scheme 64:**
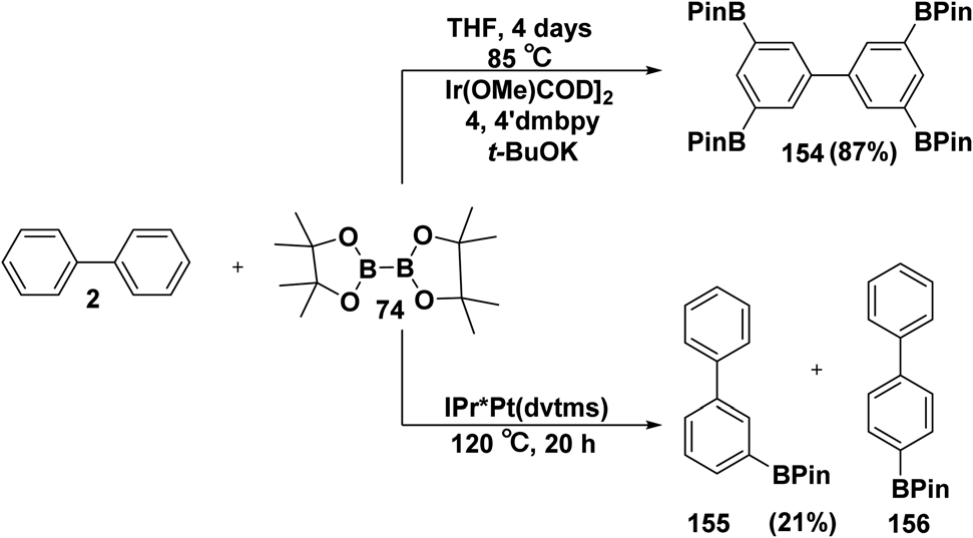
Borylation of biphenyl.

The borylation reaction of biphenyl (2) with B_2_pin_2_74 in the presence of potassium *t*-butoxide (*t*-BuOK) and (1,5-cyclooctadiene) (methoxy)iridium(i) dimer ([Ir(OMe)COD]_2_) catalyst in THF with dimethylbipyridyl (dmbpy) afforded tetrakis(Bpin)biphenyl 154.^162^ On the other hand, refluxing of biphenyl 2 with B_2_pin_2_74 and a catalytic amount of *N*-heterocyclic carbene platinum(0) complex (IPr*Pt(dvtms)) gave two different oriented products namely, *meta*-(Bpin)biphenyl (155) and *para*-(Bpin)biphenyl (156) (Scheme 64).^163^

### 3.17 MCRs for the synthesis of biphenyl amide derivative

**Scheme 65:**
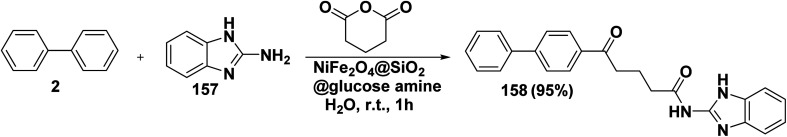
MCRs for the synthesis of biphenyl amide derivative.

Multicomponent reaction (MCRs) of biphenyl 2, 2-aminobenzoimidazole (157), and dihydro-2*H*-pyran-2,6-dione in the presence of 4-amino glucose functionalized silica propyl coated nickel ferrite nanoparticle (NiFe_2_O_4_@SiO_2_-propyl@glucosamine) catalyst and H_2_O were stirred at room temperature for 1 h afforded biphenyl amide derivative 158 (Scheme 65).^164^

### 3.18 Cyanation reaction of biphenyl

**Scheme 66:**
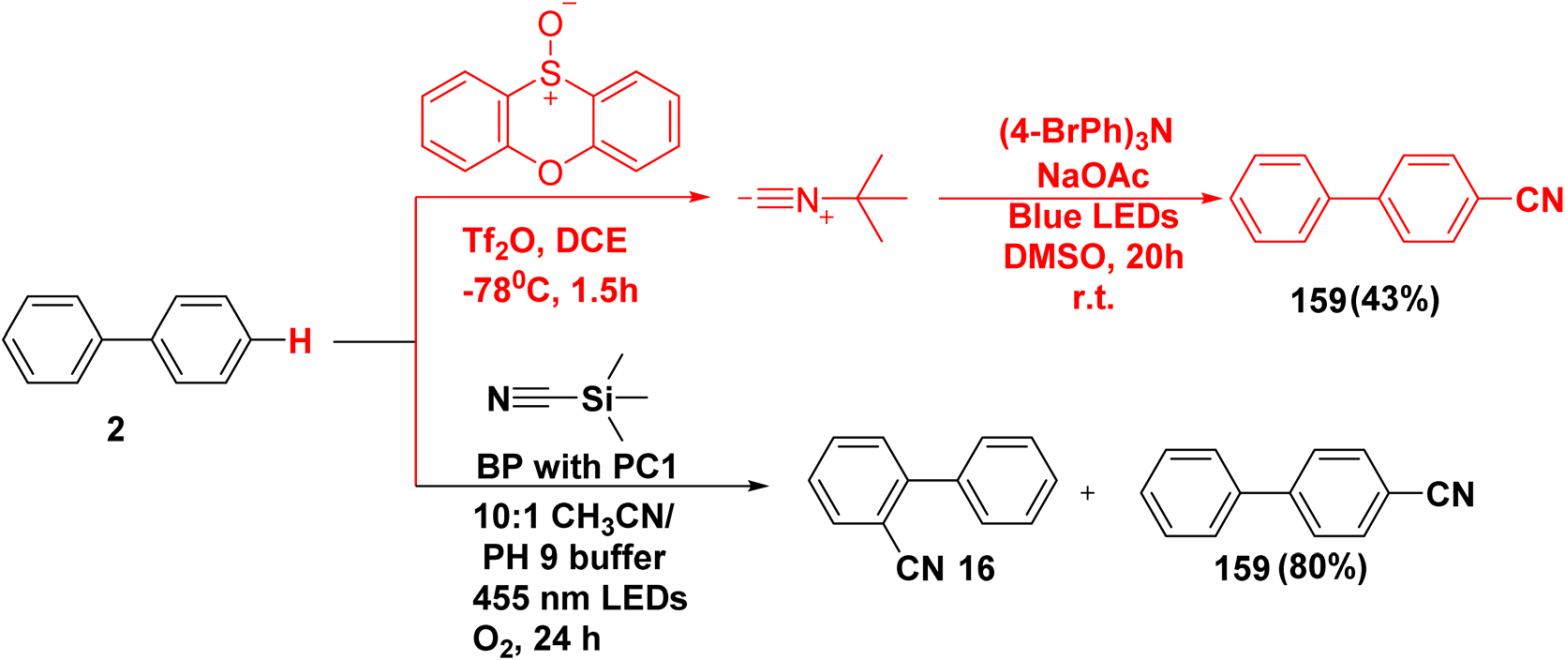
Construction of biphenyl nitrile derivative.

One-pot arene C–H cyanation reaction of biphenyl 2, phenoxathiine-10-oxide and *tert*-butyl isocyanide mediated by photoactive electron acceptor donor complex (EDA) such as tris(4-bromophenyl)amine in the presence of Tf_2_O, DCE and basic additive (NaOAc) under blue-light irradiation produced biphenyl carbonitrile 159 (Scheme 66).^165^ Whereby, phenylbenzonitrile derivatives 159 and 16 were obtained *through* the reaction of biphenyl (2) with trimethylsilyl cyanide (TMSCN) in the presence of acridinium photocatalyst and phosphate buffer in O_2_-sparged CH_3_CN.^166^

**Scheme 67:**
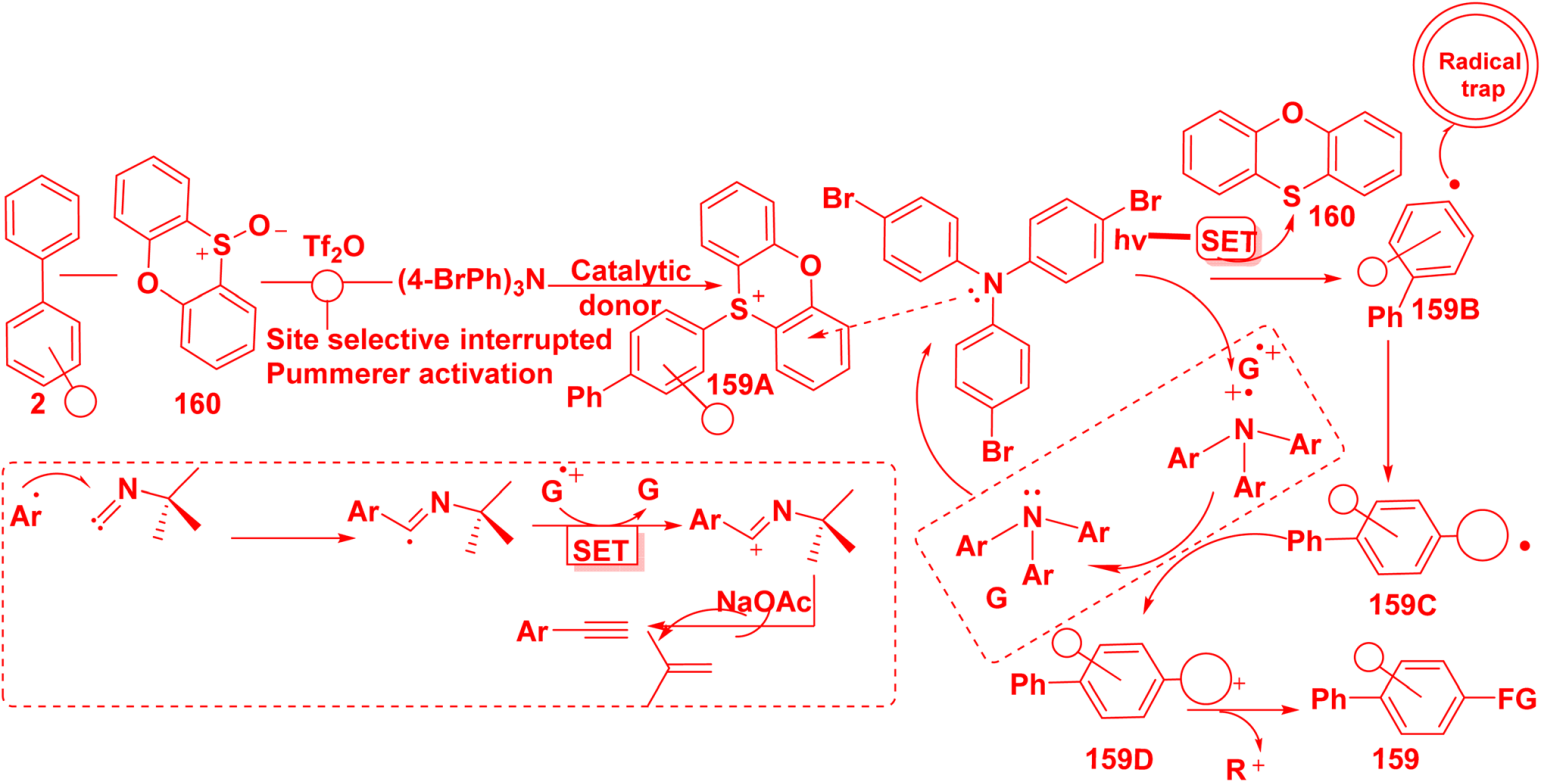
The postulated mechanism for biphenyl carbonitrile synthesis.

According to the proposed mechanism supporting the photochemical arene C–H functionalization method. Sulfonium salt 159A is produced by site-selective addition of heteroaromatic sulfoxides 160 to unfunctionalized biphenyl 2*via* interrupted Pummerer reactivity. The sulfonium salt 159A label's electron-deficient heteroaromatic system can form photoactive electron donor–acceptor (EDA) complexes with triaryl amine donors. Blue-light irradiation of the EDA complex causes single-electron transfer (SET) from the amine donor to salt 159A, which forms the radical cation of the electron donor and stimulates the synthesis of the aryl radical 159B. The radical trap 159B intercepting the aryl radical intermediate produces open shell species 159C, which are prone to oxidation to yield cations 159D, then the active electron-donor catalyst is regenerated. Finally, fragmentation of either *tert*-butyl isocyanide cation from 159D yields the required C–H functionalization products 159 (Scheme 67).^165^

**Scheme 68:**
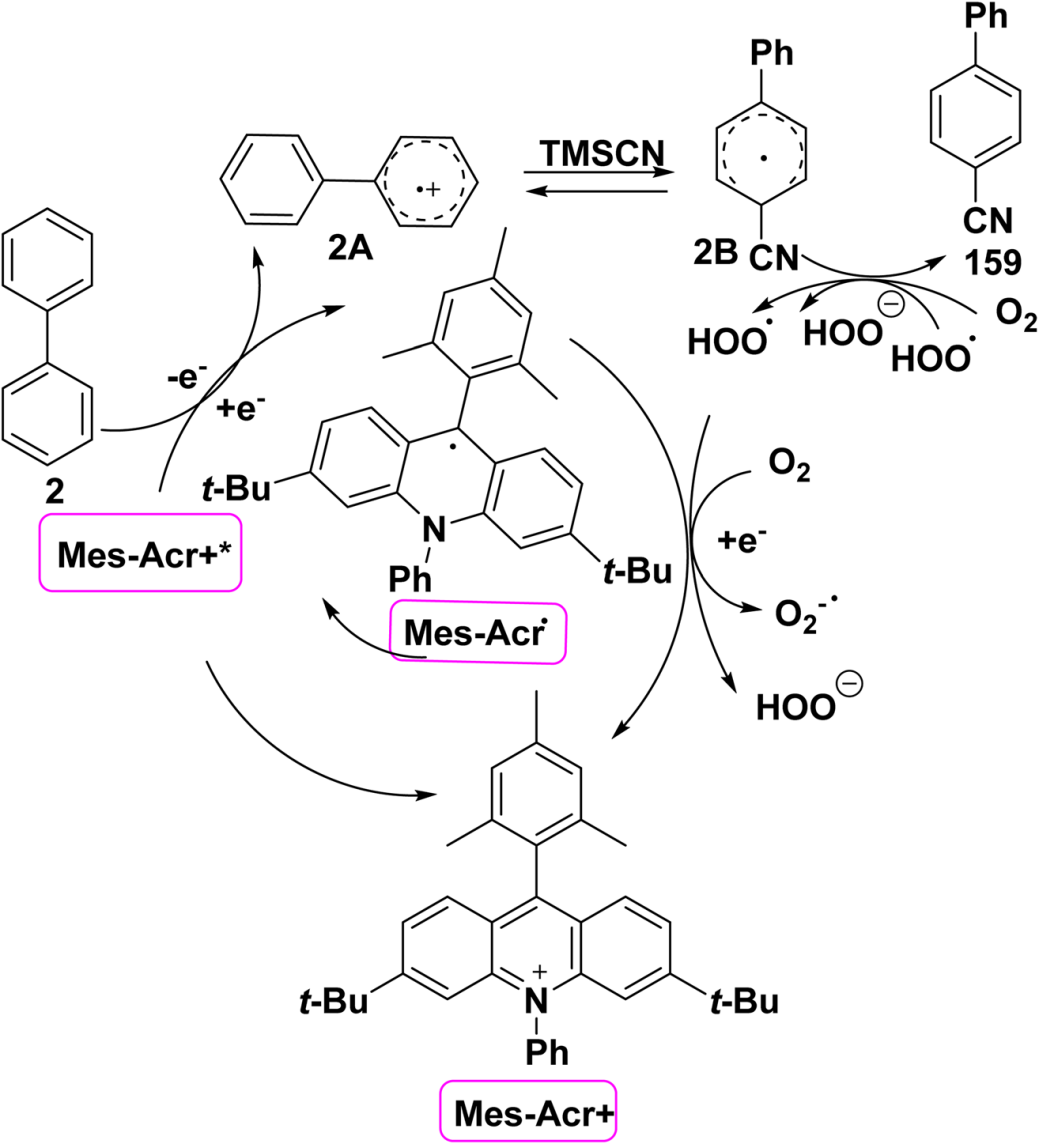
The suggested mechanism for the synthesis of cyano biphenyl.

The proposed mechanism for phenylbenzonitrile synthesis 159 starts with photo irradiation of acridinium catalyst to its oxidizing excited state Mes-Ac^+^*, followed by oxidation of biphenyl 2 to its radical cation 2A to produce the reduced acridinium Mes-Ac radical. Then, cyanide engages with 2A at *ortho* or *para* position. According to Fukuzumi cyclohexadienyl radical (2B) undergoes oxidation using molecular oxygen to give phenylbenzonitrile 159. Finally, molecular oxygen or hydroperoxy radical oxidizes Mes-Ac radical to reproduce the catalyst (Scheme 68).^166^

### 3.19 Reaction of biphenyl with sulfonium salt

**Scheme 69:**
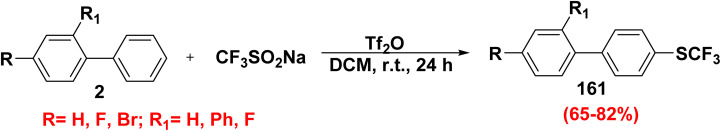
Trifluoromethylthiolation of biphenyl derivatives 161.

Several methods reported for the introduction of SCF_3_ group into biphenyl derivative, for example stirring of sodium trifluoromethanesulfinate (CF_3_SO_2_Na) with biphenyl derivative 2 in trifluoromethanesulfonic anhydride (Tf_2_O) and DCM at ambient conditions furnished [1,1′-biphenyl]-4-yl(trifluoromethyl)sulfane derivatives 161 in range yields (65–82%) (Scheme 69).^167^

**Scheme 70:**
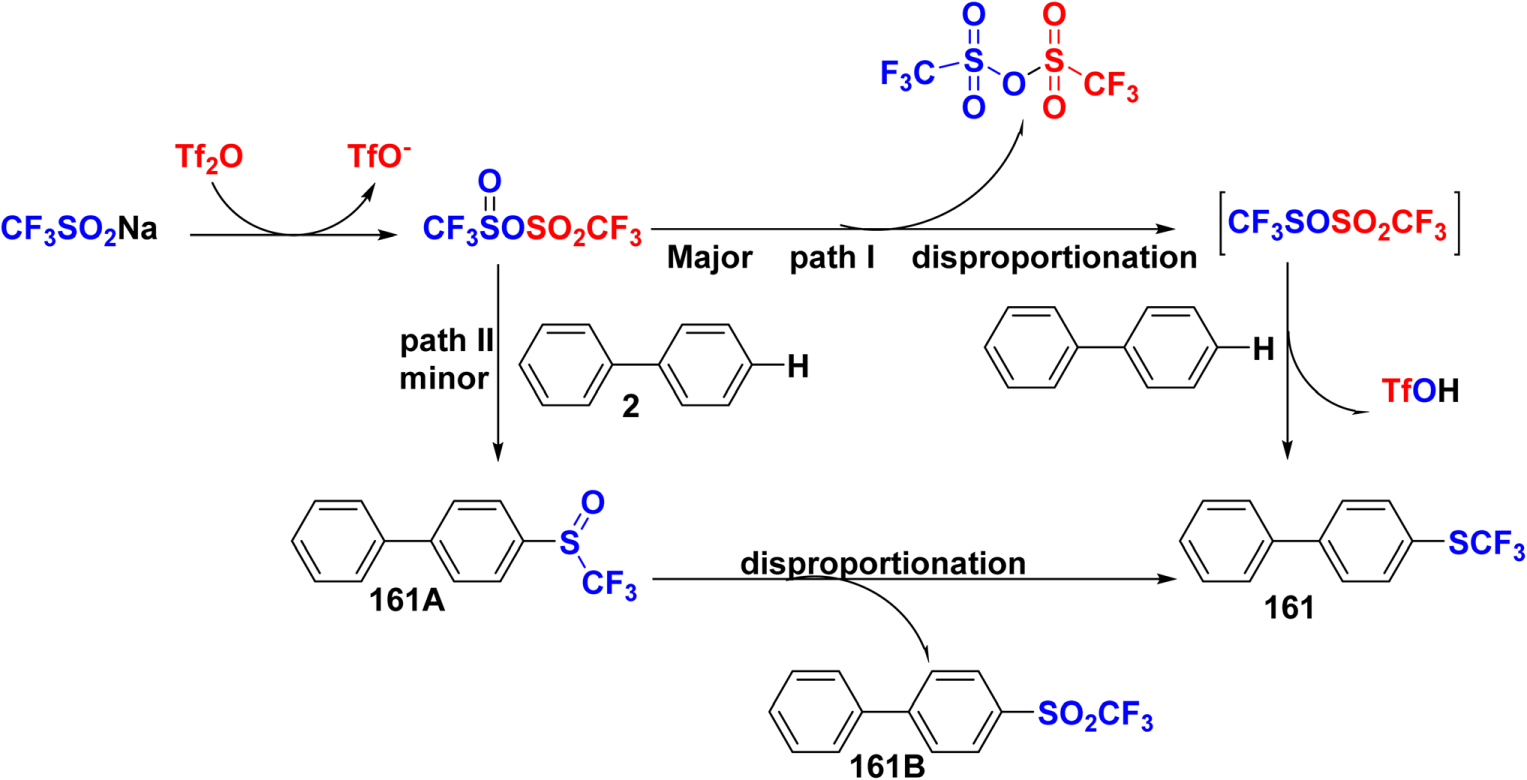
Mechanism pathway for biphenyl SCF_3_ synthesis.

The proposed mechanism for the synthesis of [1,1′-biphenyl]-4-yl(trifluoromethyl)sulfane 161 starts with the promotion of trifluoromethylthiolation using Tf_2_O and CF_3_SO_2_Na to afford an intermediate sulfonate sulfinate anhydride *through* self-disproportionation. Two possible routes lead to the final product. In path I, half of sulfonate sulfinate anhydride intermediate was oxidized to form TfO-SO_2_CF_3_ and another part is reduced to form CF_3_SOSO_2_CF_3_. Finally, the transfer of trifluoromethylthiolation from CF_3_SOSO_2_CF_3_ to biphenyl (2) to afford SCF_3_ product 161 (Scheme 70).^167^

### 3.20 Reaction of biphenyl with azole

**Scheme 71:**
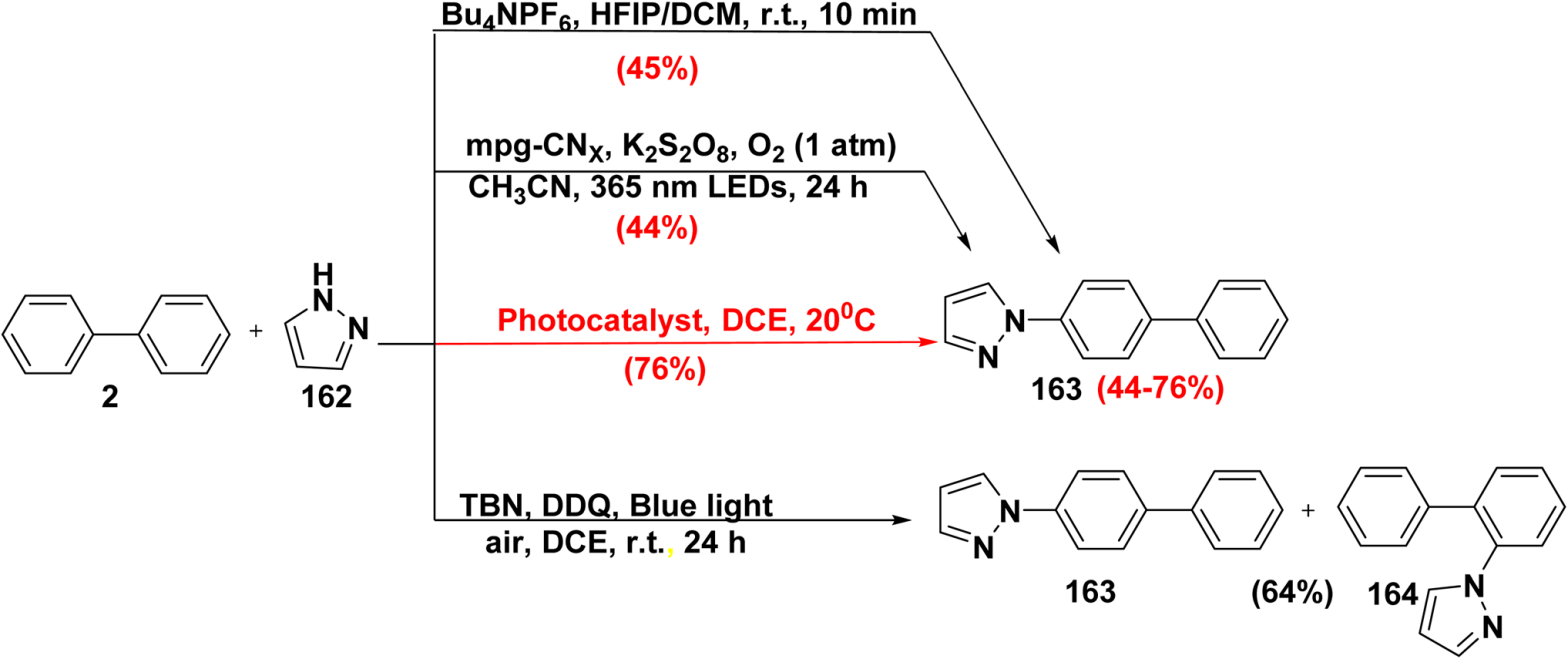
Synthesis of biphenyl pyrazole derivative.

*N*-Aryl azoles are a key motif in a number of biologically active compounds used in medicinal chemistry and crop protection science. The reaction of biphenyl (2) with parent pyrazole 162 in a mixture of HFIP and DCM,^168^ or in the presence of dipotassium peroxodisulfate (K_2_S_2_O_8_) and CH_3_CN under O_2_ atmosphere produced 4′-(1*H*-pyrazol-1-yl)-[1,1′-biphenyl]-4-ylium (163).^169^ Whereby, Sen *et al.*^170^ reported oxidative azolation of unactivated biphenyl derivatives *via* using phenalenyl-based organic Lewis acid as powerful photooxidant in DCE to afford compound 163. While, under blue light irradiation, the photocatalytic oxidation of biphenyl 2 with azole 162 using DDQ catalyst and *tert*-butyl nitrite (TBN) yielded 1*H*-pyrazol-biphenyl-4-ylium 163 and 1-([1,1′-biphenyl]-2-yl)-1*H*-pyrazole (164) (Scheme 71).^171^

### 3.21 Reaction of related biphenyl

**Scheme 72:**
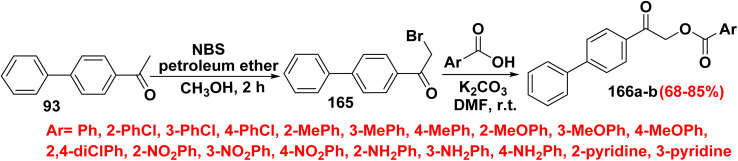
Formation of biphenyl ester.

The anti-tyrosinase properties of biphenyl ester makes it effective for treating dermatological conditions and for cosmetic lightening. A series of [1,1′-biphenyl]-4-yl-oxoethylbenzoate derivatives 166 were produced *through* two stages. First step involving a mixture of NBS, and 1-([1,1′-biphenyl]-4-yl)ethan-1-one (93) was refluxed in a mixture of petroleum ether (PET) and methanol for 2 h afforded 1-([1,1′-biphenyl]-4-yl)-2-bromoethan-1-one (165). In the second stage, compound 165 was reacted with different phenyl carboxylic acid derivatives in the presence of K_2_CO_3_ in DMF at room temperature led to formation of 2-([1,1′-biphenyl]-4-yl)-2-oxoethyl benzoate derivatives 166a. Biphenyl-4-yl-oxoethyl-pyridine carboxylate derivatives 166b were obtained by stirring of 1-([1,1′-biphenyl]-4-yl)-2-bromoethan-1-one (165) with pyridine carboxylic acid derivatives in DMF containing anhydrous K_2_CO_3_ (Scheme 72).^10^

**Scheme 73:**
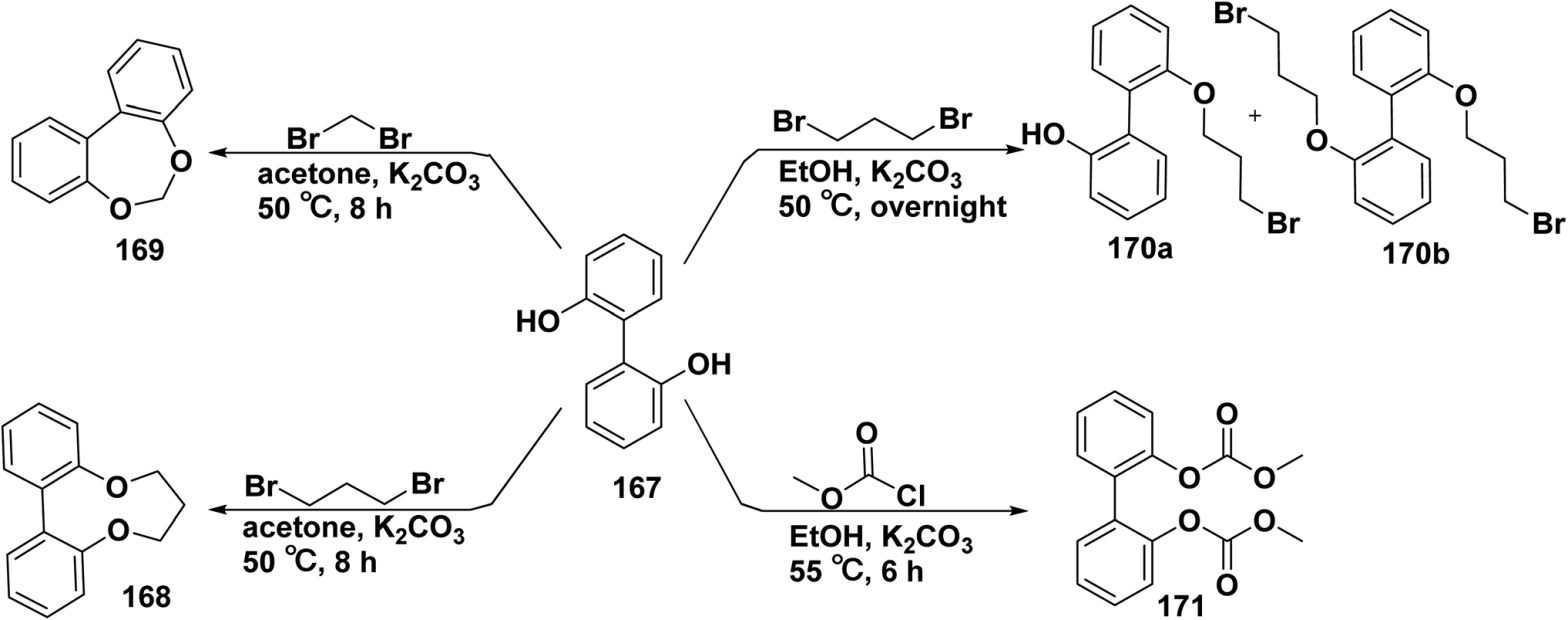
Reaction of biphenyl diol with various halo-derivatives.

Compounds containing biphenyl underwent electrophilic substitution to aromatic substrates. Dihydro-6*H*-dibenzo[1,5]dioxonine 168 was produced by refluxing biphenyl-diol 167 with dibromopropane in acetone containing catalytic amount of anhydrous K_2_CO_3_. Whereas, under the same condition, refluxing of biphenyl-2,2′-diol (167) with dibromomethane afforded dibenzo[1,3]dioxepine 169. Furthermore, treatment of biphenyl-2,2′-diol (167) with 1,3-dibromopropane and K_2_CO_3_ in EtOH gave a mixture of 2′-(3-bromopropoxy)-[biphenyl]-2-ol 170a and bis(3-bromopropoxy)biphenyl 170b. In the same manner, a mixture of biphenyl-diol derivative 167 and methyl carbonochloridate in EtOH afforded biphenyl derivative 171. It is worthy to mention that *o*-alkylated biphenyl derivatives exhibit excellent scavenging of superoxide anion radical and β-glucuronidase inhibition activity (Scheme 73).^12^

**Scheme 74:**
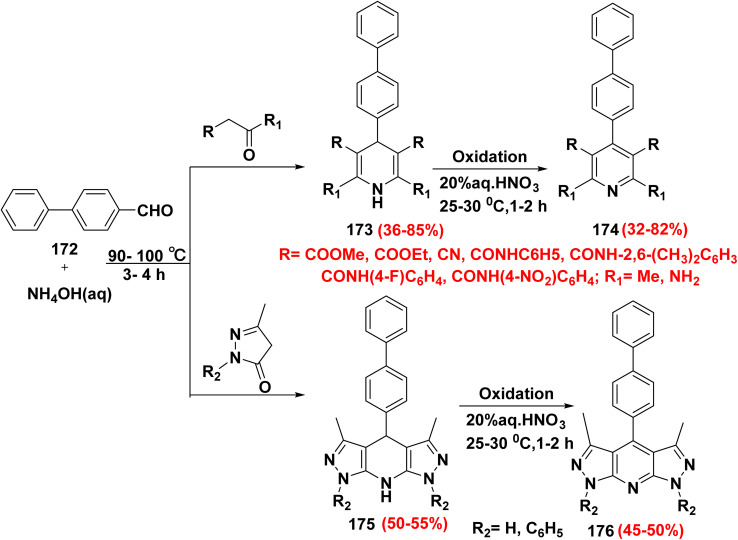
Suzuki–Miyaura and Hantazch reaction for preparation of annulated pyridine systems.

According to Suzuki–Miyaura and Hantazch reactions, treatment of 4′-formylbiphenyl (172) with active methylene namely acetone derivatives or substituted pyrazole derivatives and ammonium hydroxide (NH_4_OH) gave tricyclic ring of 4-biphenyl-1,4-dihydropyridine derivatives 173 and pentacyclic ring of [4-biphenyl]-1,7-dihydrodipyrazolo[3,4-*b*]pyridine derivatives 175, respectively. Then, oxidation of 173 and 175 with nitric acid (HNO_3_) afforded 4-(biphenyl-4-yl)-pyridine derivatives 174 and 4-biphenyl-dipyrazolo[3,4-*b*]pyridine derivatives 176, respectively which are used as anti-microbial resistance, antibacterial, antifungal and anticancer (Scheme 74).^172^

**Scheme 75:**
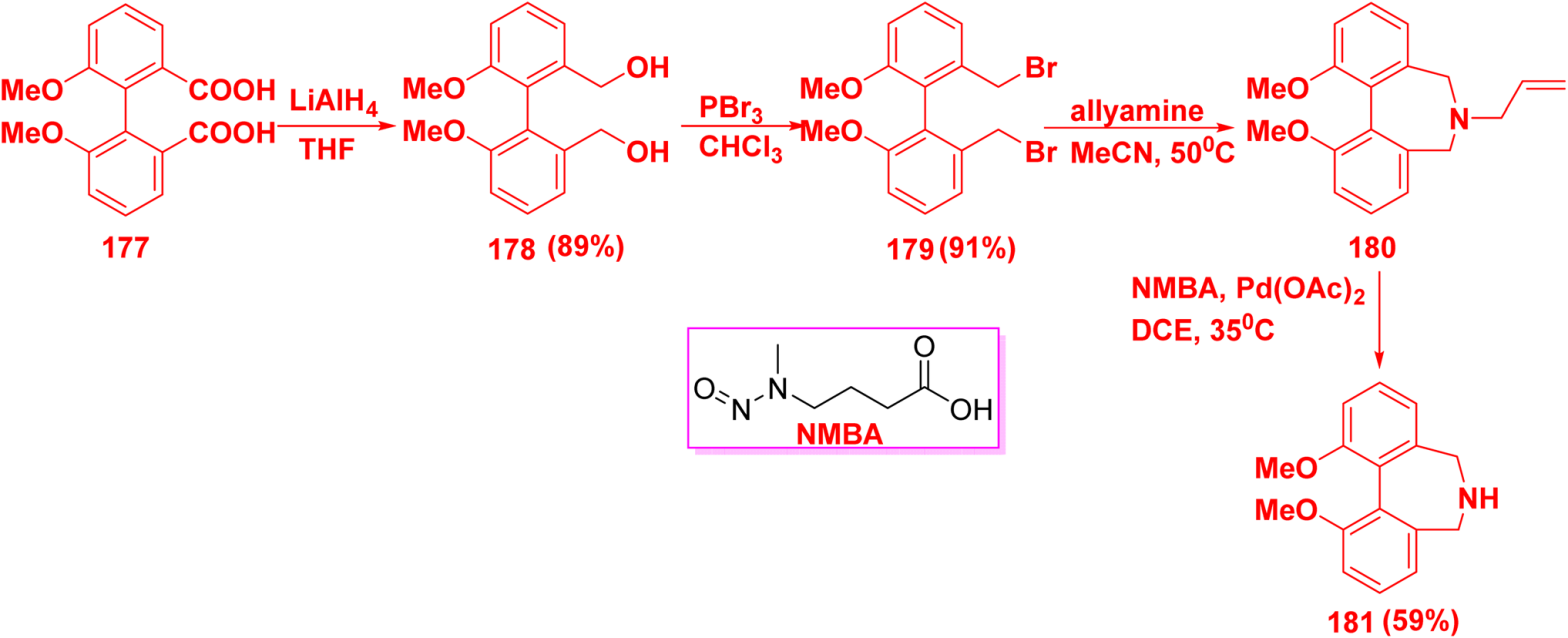
Synthesis of biphenyl diazepine derivative 181.

Reduction of biphenyl dicarboxylic acid 177 with lithium aluminum hydride (LiAlH_4_) afforded biphenyl diol 178, followed by bromination reaction with phosphorus tribromide (PBr_3_) produced biphenyl dibromide 179. Then, compound 179 underwent cyclization reaction to produce azepine derivative 180. Finally, dealkylation of compound 180 furnished biphenyl diazepine derivative 181 (Scheme 75).^173^

## 4. Biphenyl atropisomerism

**Fig. 3:**
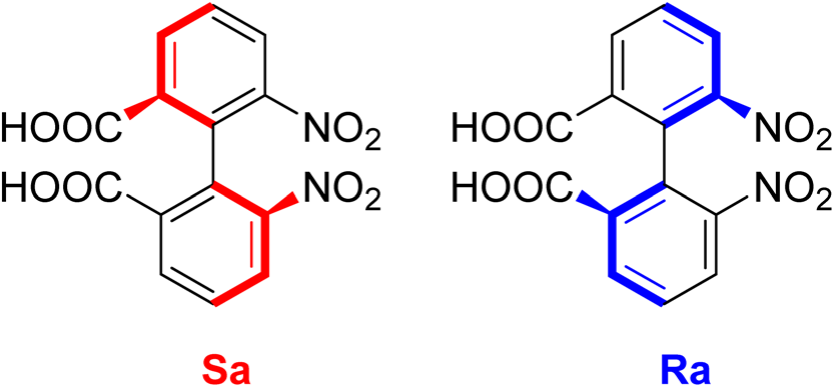
Atropisomerism of biphenyl compound 2.

As reflected in the last few years, the crest of a sizable wave in the development of new ligands in drug discovery and medicinal chemistry, the recent literature addressing atropisomerism has been hard to miss, as biphenyl atropisomers have received considerable attention as chiral building blocks for chiral catalysts in asymmetric syntheses field. The first experimentally described molecule with atropisomerism phenomenon was 6,6′-dinitro-2,2′-diphenic acid by Christie and Kenner in 1922.^174,175^ As the inclusion of the biphenyl isosteres represent 4.3% of all known drugs.^176^ Atropisomerism is defined as stereochemistry arising from highly hindered bond rotation that creates a chiral axis. The rotational barrier of an atropisomeric molecule is sufficiently high to allow for isolation of the individual conformers. Whereby, atropisomeric pharmaceutical ingredients can differ in their pharmacokinetic and selectivity properties towards a hitting target (Fig. 3).^177–183^

## 5. Applications

**Scheme 76:**
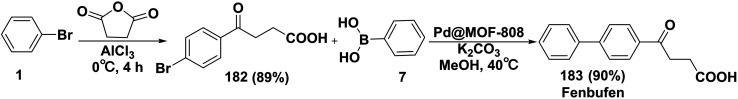
Synthetic strategy for the preparation of fenbufen.

Fenbufen is a non-steroidal anti-inflammatory, antipyretic effects (NSAIDs) and analgesic drug with less irritative effect on gastrointestinal tract (Table 1). The synthesis of fenbufen (183) was performed in two steps. First, Friedel–Crafts acylation reaction of bromobenzene (1) and succinic anhydride catalyzed by AlCl_3_ afforded 1-(4-bromophenyl)-4-hydroperoxybutan-1-one (182), then coupling the product 182 with phenyl boronic acid (7) (Scheme 76).^15^

**Table 1:** Examples of a vast array of biologically active molecules towards some diseases

Structure	Activities	Action mechanism & references
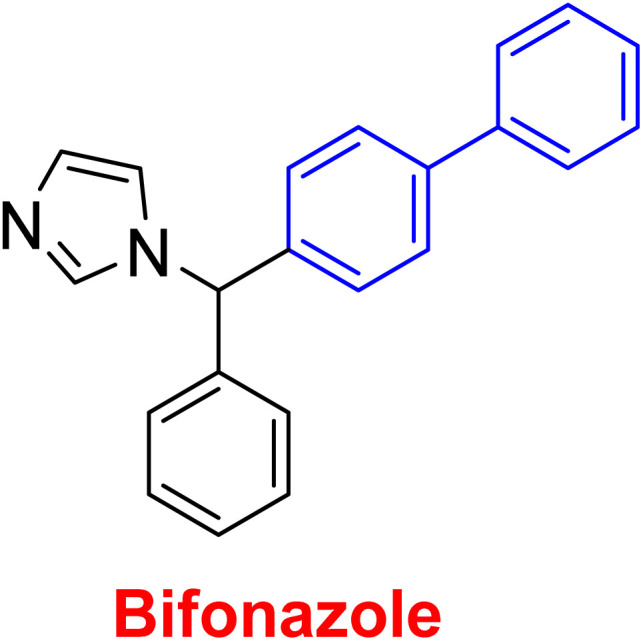	Antibacterial: (*Staphylococcus aureus* and *Salmonella* spp.)	The inducing of chlorine atoms on the biphenyl ring connected to the imidazole ring led to alterations in the physico-chemical properties, therefore these factors can affect the affinity of molecules for the iron of the heme binding site.^184^ Addition of biphenyl moiety improves its antifungal selectivity, potency and bioisosteric effects^185^
	Therapy for dermatomycoses	
	Antifungal: (*Candida albicans*, *Cryptococcus neoformans* and *Aspergillus fumigatus*)^184,185^	
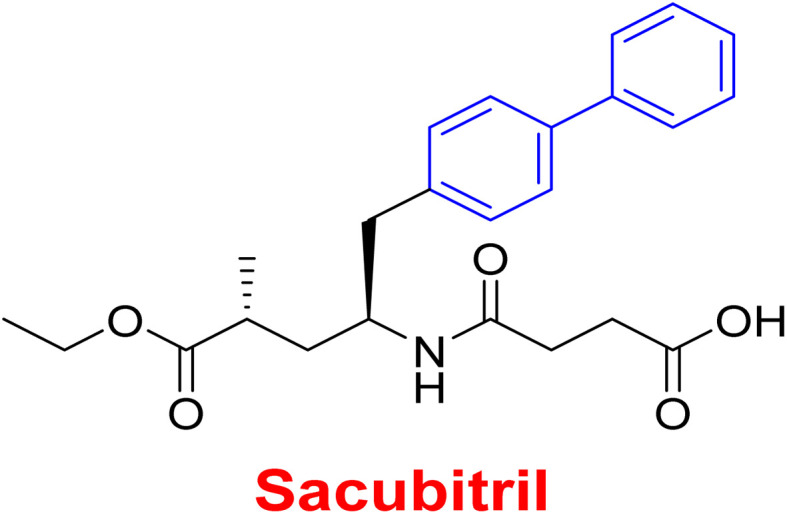	Anti-tubercular: (dormant tuberculosis)	Due to the presence of substituted biphenyl increases the polarity of the heterocyclic skeleton which might be the reason for high anti-tubercular (anti-TB) activities *via* interacting with the MurB inhibitors^186^
	Treatment of heart failure with a combination of valsartan	
	Neprilysin inhibitor^186,187^	
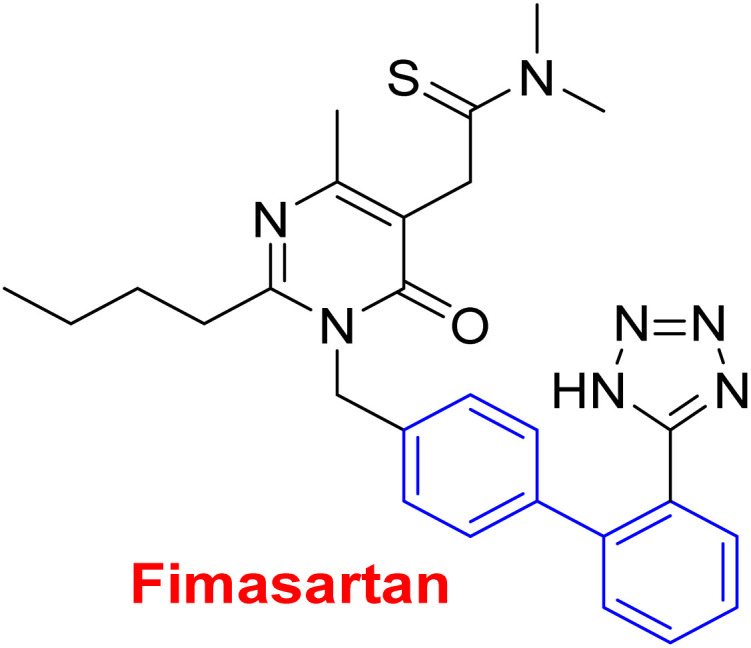	Antihypertensive	Since inhibition of angiotensin II type 1 (AT1) receptor reduces chronic inflammation associated with hypertension, we evaluated the anti-inflammatory potential and the underlying mechanism of fimasartan^188^
	Anti-inflammatory^188^	
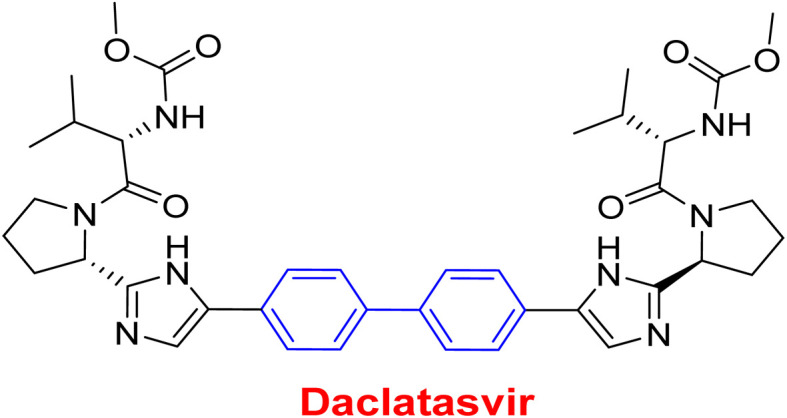	Antiviral	As a result of sp^2^ hybridization of CNTs between the drug and target protein sequence, leads to improving the fluorescence reactivity. Because the conjugated system of biphenyl and the presence of Cs/CNT can increase the electroactive surface area of the electrode, leading to an increase in the number of structural flaws^189^
	Treatment selections for hepatitis C virus^189,190^	
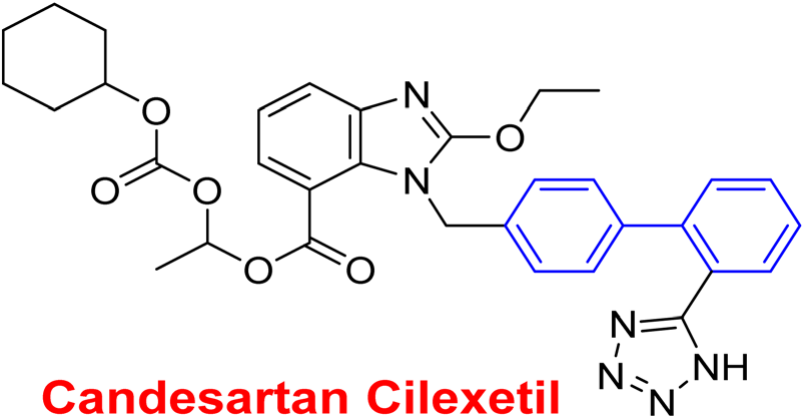	Antihypertensive	Biphenyl acts as lipophilic moiety in candesartan drug; whereas the candesartan is a selective nonpeptide angiotensin II type 1 (AT1) receptor antagonist which reduces blood pressure effectively.^191^ Sartans incorporates with membrane receptor in lipid bilayers causing highly transportation possibility of sartans *via* the receptor^192^
	Active for the AT1 receptor^191–193^	
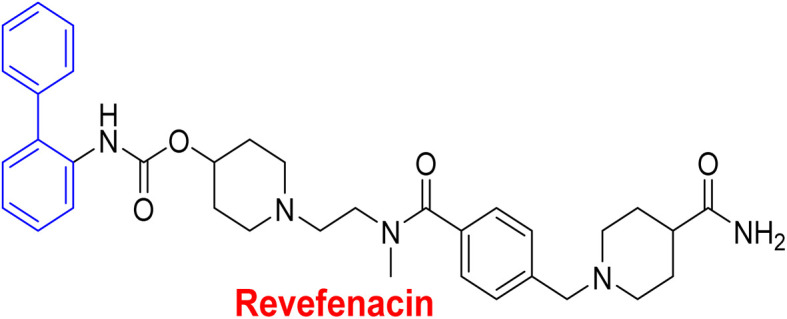	Lung-selective muscarinic cholinergic receptor (mAChR) antagonist	Biphenyl moiety enhances long-lasting and potency of mediated antagonism of mAChR-causing contraction of human bronchial tissues^194^
	Nebulized inhalation solution to produce long-acting bronchodilation^194^	
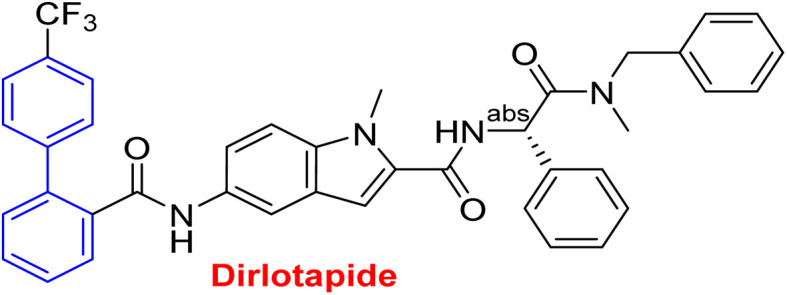	Microsomal triglyceride transfer protein inhibitor	Biphenyl scaffold for nesting
	Treatment for human dyslipidemias^195^	
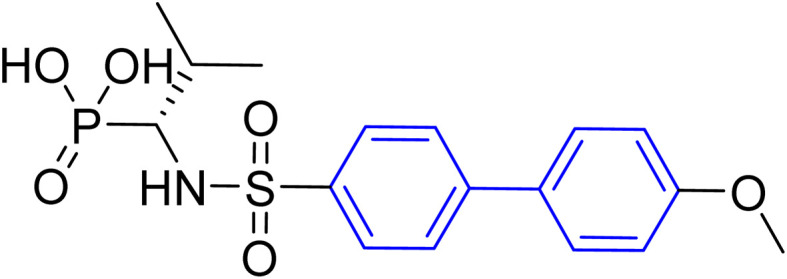	Anti-human neutrophil collagenase (MMP-8)^196^	Biphenyl residues hit the active site close the catalytic zinc ion that would consequently inhibit the collagenase activity^196^
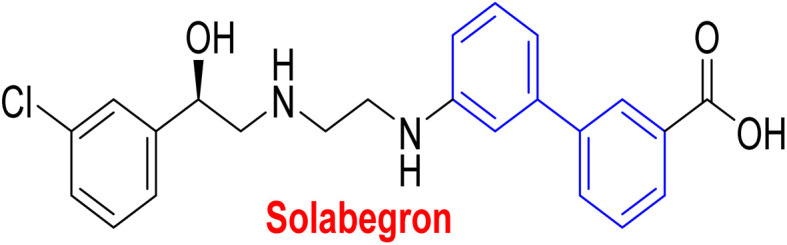	β_3_-Adrenergic receptor agonist	RHS of biphenyl ring affords potent human β3-AR agonists with a chlorophenyl ring on the LHS side^197^
	Evokes bladder relaxation	
	Overactive urinary bladder	
	Increases micturition reflex threshold in the dogs^197–199^	
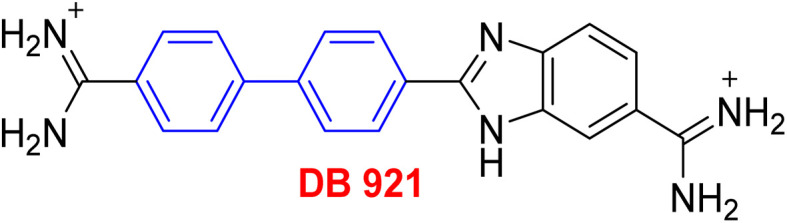	Analysis of water mediated binding in the context of a DNA complex	The interactions of the molecules containing of biphenyl with DNA AT sites increasing DNase I footprinting depending on increasing conjugation process which enhancing biosensor-surface plasmon resonance, circular dichroism microcalorimetry, and isothermal titration^200^
	Promising agent against parasites	
	Change in AT sequences with destruction of the kinetoplast and cell death^200^	
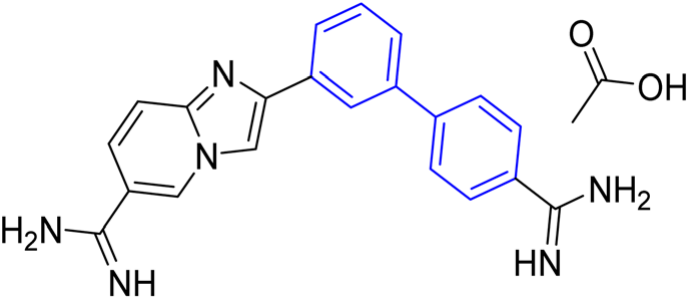	Antiprotozoal	Biphenyl scaffold for nesting
	Anti-trypanosomal^201^	
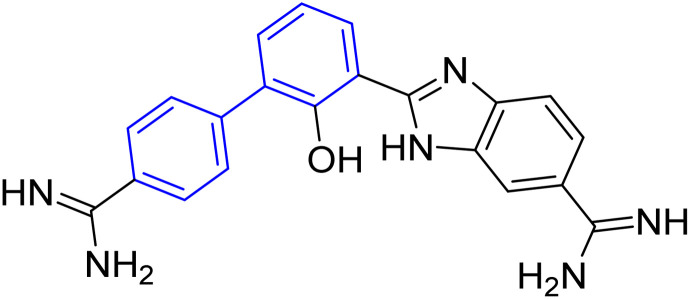	Anti-protozoan^202^	Biphenyl scaffold for nesting
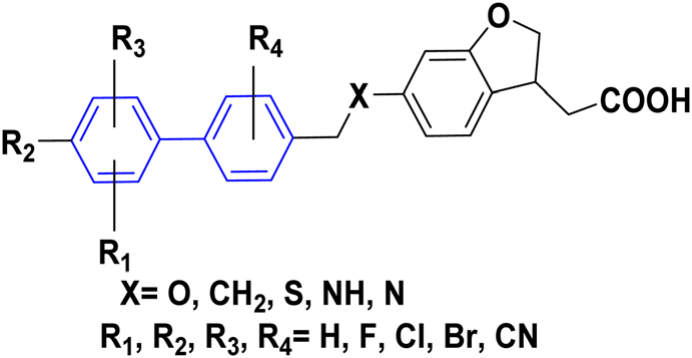	Treating diabetes mellitus^203^	Biphenyl scaffold for nesting
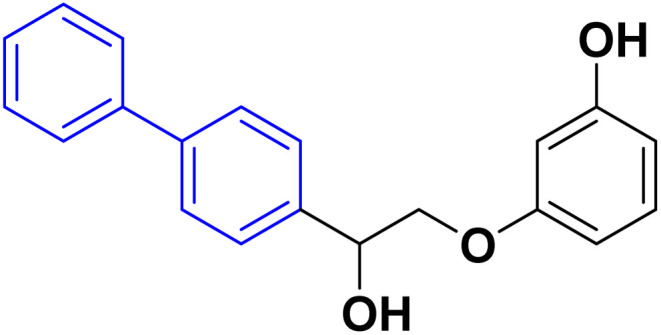	Treatment CNs impairments involving major depressive disorder^204^	Biphenyl-based NMDA negative allosteric modulator (NAM) has low affinity for the human ether-a-go-go-related gene ion channel (hERG) and the dynamics calculations suggest a various binding mode (ifenprodil-like) compared to another biaryl-based NMDA NAM EVT-101 ^204^

**Scheme 77:**
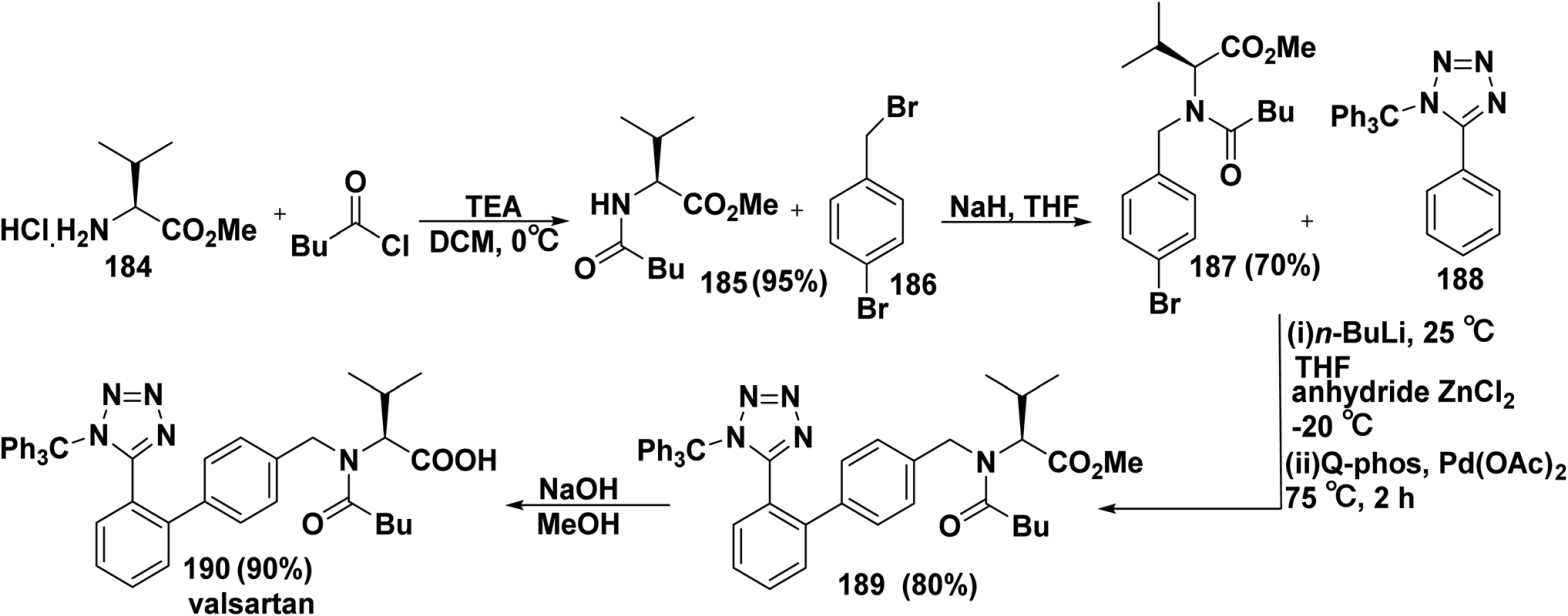
Negishi coupling reaction for valsartan synthesis.

The trade name for valsartan is Diovan, valsartan belongs to the non-peptide angiotensin (II) receptor class and is used to treat cardiovascular disorders that are connected to hypertension. Synthesis of valsartan (190) began with coupling reaction of l-valine methyl ester hydrochloride (184) with valeryl chloride in the presence of triethylamine (Et_3_N) in DCM to produce methyl *N*-pentanoyl-l-valinate (185). N-Protection of compound (185) with 1-bromo-4-(bromomethyl) benzene (186) in the presence of sodium hydride (NaH) in THF furnished methyl *N*-(4-bromobenzyl)-*N*-pentanoyl-d-valinate (187). Then, organozinc compound was synthesized *via o*-metalation of 5-phenyl-1-trityl-1*H*-tetrazole (188) with *n*-butyllithium (*n*-BuLi), followed by adding zinc chloride (ZnCl_2_). Negishi coupling reaction of organozinc chloride compound with aryl bromide 187 in the presence of palladium acetate (Pd(OAc)_2_) with 1,2,3,4,5-pentaphenyl-1′-(di-*tert*-butylphosphino)ferrocene (Q-phos) ligand in THF afforded *N*-pentanoyl-*N*-(2′-(1-trityl-1*H*-tetrazol-5-yl)-[1,1′-biphenyl]-4-yl)-valinate (189). Finally, valsartan 190 was produced *via* hydrolysis of ester 189 with sodium hydroxide (Scheme 77).^205^

**Scheme 78:**
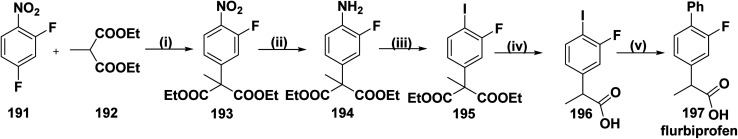
Synthesis of flurbiprofen. Reagents and conditions: (i) NaH, DMSO; (ii) H_2_, Pd/C; (iii) 1. NaNO_2_, HCl, 0 °C; 2. KI; (iv) NaOH, EtOH–H_2_O, 100 °C, 57% (four steps); (v) PhB(OH)_2_, Pd(PPh_3_)_4_, Na_2_CO_3_, DME-H_2_O, 80 °C.

The synthesis of 2-fluoro-[1,1′-biphenyl]-4-yl-propanoic acid which trade name mark (flurbiprofen) 197 has been achieved in subsequent steps. First step involving the reaction of 2,4-difluoronitrobenzene (191) with diethyl 2-methylmalonate (192) in the presence of NaH to afford diethyl 2-(3-fluoro-4-nitrophenyl)-2-methylmalonate (193), followed by catalytic hydrogenation of compound 193 to 4-amino-3-fluorophenyl-2-methylmalonate 194. After that, diazotization of the amino compound 194 into corresponding diazonium salt which then reacted with potassium iodide (KI) to produce diethyl 2-(3-fluoro-4-iodophenyl)-2-methylmalonate (195). Compound 195 underwent hydrolysis and decarboxylation reaction to yield 2-(3-fluoro-4-iodophenyl)propanoic acid (196). Finally, SMC reaction of 3-fluoro-4-iodophenylpropanoic acid 196 with phenylboronic acid in the presence of a catalytic amount of Pd(PPh_3_)_4_, 1,2-dimethoxyethane and Na_2_CO_3_ produced flurbiprofen (197). Flurbiprofen, a prostaglandin biosynthesis inhibitor, is extensively used as potent anti-inflammatory, antifungal, antipyretic, analgesic, in addition to, treatment Alzheimer's disease (AD) (Scheme 78).^206,207^

**Scheme 79:**
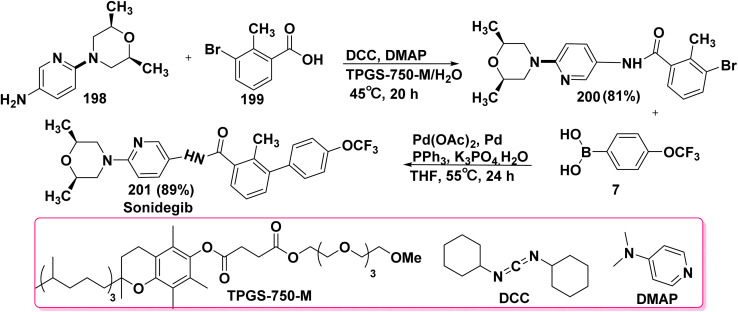
SMC reaction for biphenyl synthesis.

Sonidegib which is marketed under Odomzo trade name is a Hedgehog pathway inhibitor. Sonidegib is effective against skin cancer, tumor, and medulloblastoma. Synthesis of sonidegib 201 started with the coupling of dimethylmorpholinopyridin-3-amine 198 with 3-bromo-2-methylbenzoic acid (199) in the presence of coupling agent such as dicyclohexylcarbodiimide (DCC) and 4-(dimethylamino)pyridine (DMAP) and TPGS-750-M-H_2_O as a surfactant to afford 3-bromo-(2,6-dimethylmorpholino)pyridin-3-yl)-2-methylbenzamide 200. Then, SMC reaction of compound 200 with (4-(trifluoromethoxy)phenyl)boronic acid (7) in the presence of Pd(OAc)_2_, PPh_3_, Pd, K_3_PO_4_ and THF (Scheme 79).^208^

**Scheme 80:**
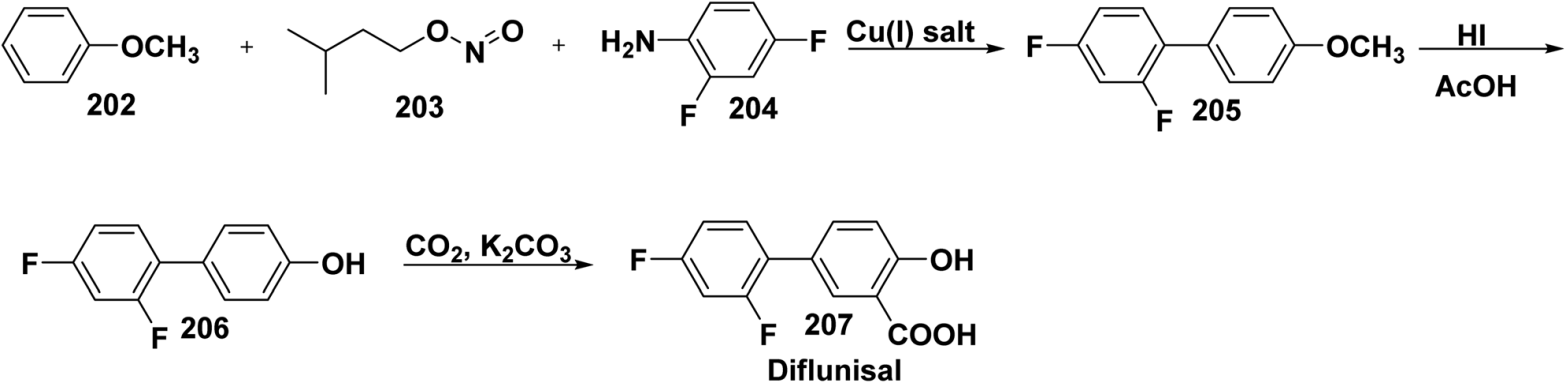
MCRs for the synthesis of diflunisal.

One-pot three component synthesis of 4-(2,4-difluorophenyl)anisole (205) *through* stirring a mixture of anisole (202), isoamyl nitrite (203), and 2,4-difluoroaniline (204) in the presence of cupper(i)salt. Demethylation reaction of difluorophenylanisole (205) with hydrogen iodide (HI) afforded 4-(2,4-difluorophenyl)-phenol (206). Kolbe–Schmitt phenol carboxylation of 206 with carbon dioxide (CO_2_) in the presence of K_2_CO_3_ afforded diflunisal (207). Whereby, diflunisal (207) has anti-inflammatory, anti-fever, and analgesic properties. In cases of osteoarthritis and rheumatoid arthritis, it is utilized to relief pain (Scheme 80).^209,210^

**Scheme 81:**
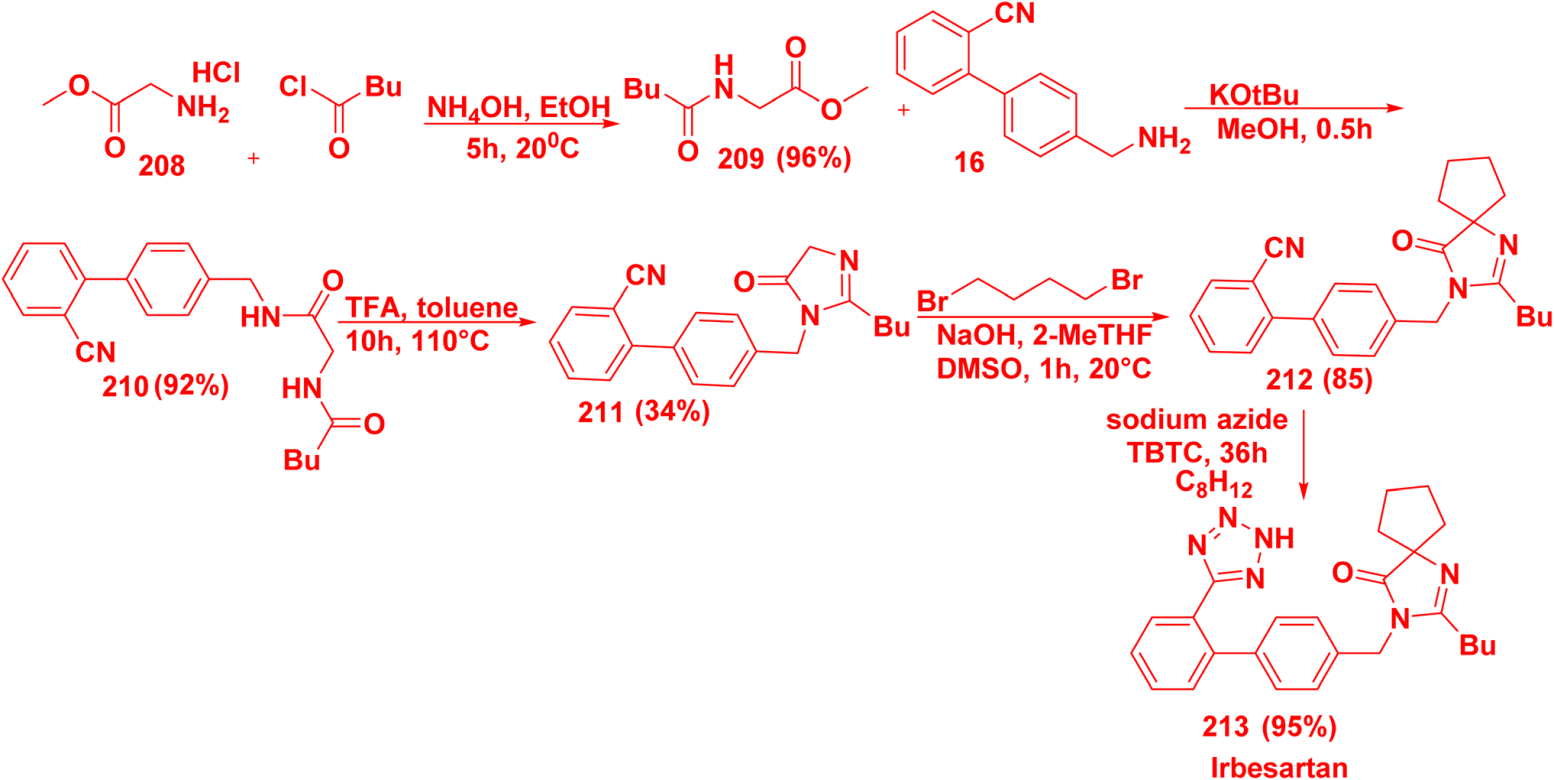
Synthetic strategies for irbesartan synthesis.

Reaction of glycine methylester 208 with pentanoyl chloride in TEA produced methyl pentanoylglycinate (209). Compound 209 reacted with biphenylcarbonitrile derivative 16 in the presence of KO*t*-Bu afforded biphenylamide derivative 210. Then, cyclization reaction of compound 210 in the presence of TFA afforded imidazobiphenyl carbonitrile derivative 211. Alkylation reaction of compound 211 with dihaloalkane furnished spirocyclic compound 212.^211^ Finally, irbesartan (213) was synthesized *via* reaction of key intermediate 212 with sodium azide, tributyltin chloride (TBTC) and dimethoxycyclohexadiene 213. In pancreatic cancer, irbesartan overcomes gemcitabine resistance by decreasing iron metabolism and stemness *via* inhibition of the Hippo/YAP1/c-Jun axis.^212^ In addition, irbesartan lowers cholesterol *via* inhibiting the expression of the low-density lipoprotein receptor. Furthermore, irbesartan is used for the treatment of hypertension and type 2 diabetes (Scheme 81).^213^

## 6. Patents

**Fig. 4:**
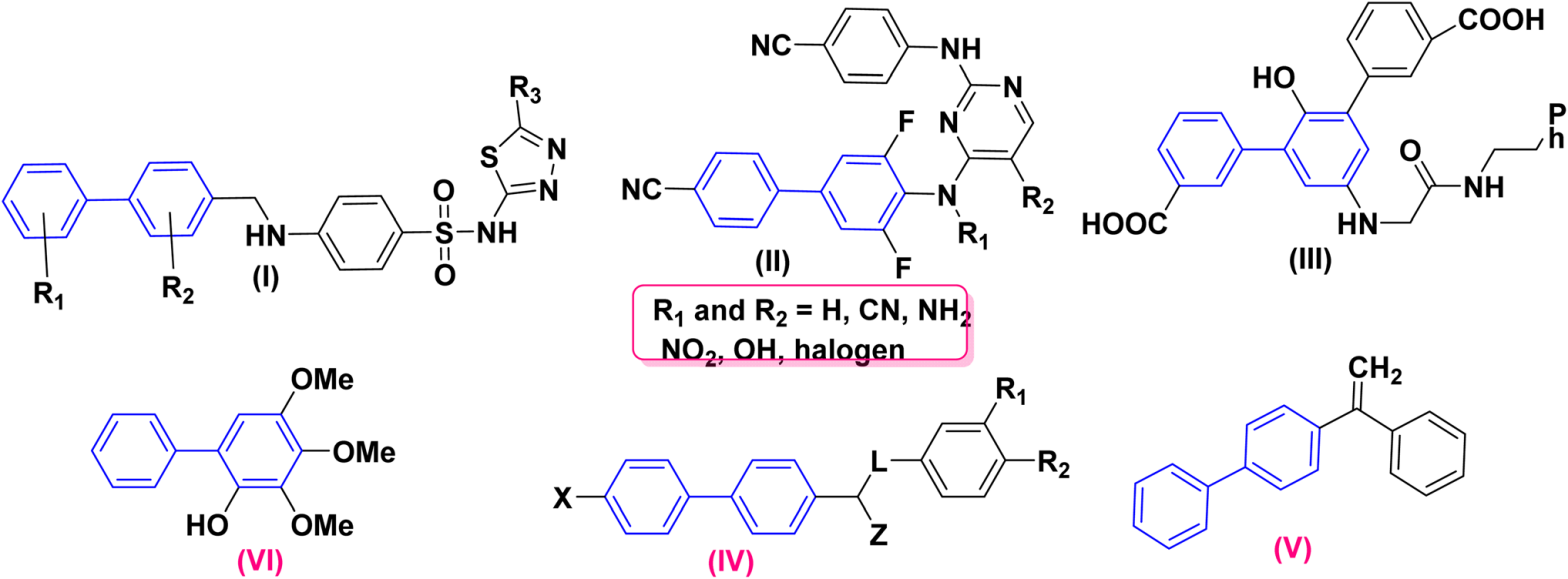
Some biologically active biphenyl derivatives.

Biphenyl sulfothiadiazole derivatives (I) are used in clinical immunotherapy of tumour patients and act as a PD1/PDL1 inhibitor.^214^ Whereas, biphenyl biaryl pyrimidine derivatives (II) are utilized to treat AIDS and are expected to be anti-HIV drugs.^215^ 5′-((2-((3,4-Dimethoxyphenethyl)amino)-2-oxoethyl)amino)-2′-hydroxy-[1,1′:3′,1′′-biphenyl]-3,3′′-dicarboxylic acid (III) is developed to treat malignant tumours and as a transcription factor.^216^ 3-(2-([1,1′-Biphenyl]-4-yl)-2-hydroxyethoxy) phenol (IV) is a therapy for a disorder or disease accompanied by high activation of the NMDA receptor.^217^ 4-(1-Substituted phenylvinyl)biphenyl derivatives (V) can be used for the manufacture of protein kinase, tubulin inhibitors and drugs for colon and breast cancer.^218^ 2-[(3,5-Difluoro-3′-methoxy-1,1′-biphenyl-4-yl)amino]nicotinic acid is used to treat psoriasis disease.^219^ (2*E*)-[2-(Biphenyl)imidazo[1,2-*a*]pyrimidine-3-yl](4-nitrophenyl)-prop-2-en-1-one, 2-(biphenyl)imidazo[1,2-*a*]pyrimidine-3-carbaldehyde and 6-[2-(biphenyl)imidazo[1,2-*a*]pyrimidine-3-yl]-4-nitro pyrimidine-2(1*H*)-one exhibit promising antitumor activity by reducing the level of liver function enzyme near the normal level after damaged by CCl_4_.^220^ Whereby, trimethoxy-[1,1′-biphenyl]-2-ol (VI) displays anti-inflammatory activity^221^ (Fig. 4).

## 7. Conclusion

With the continuously increasing importance of biphenyl scaffolds as the structurally decisive scaffolds in bioactive natural products and pharmaceuticals drugs, formidable efforts have been carried out to summarize gradual developments of biphenyl compounds and their isosteres *via* metallic catalyzed reaction which covered the literature reported in the last decades based on this thrilling area of research. In this review several chemical reactions of biphenyl with different reagents have been discussed. The profound discussion of different captivating mechanisms associated with the role of numerous key catalysts and reagents *through* this review is believed to be beneficial for the synthetic community to apply these methods for practical purposes. Additionally, the efficacy of biphenyl compounds in medicinal chemistry and academic industry has been covered, and thereby will be helpful for future research. Although, obtaining these frameworks with particular substitution patterns is still an intimidating exercise. Atropisomerization phenomena of biphenyl derivatives also has taken into account Therefore, we believe that this review can be a guiding principle for synthetic and medicinal chemists working in this field.

## Conflicts of interest

The authors confirm that this article content has no conflict of interest.

## Abbreviations

H_2_NCO_2_^−^H_4_N^+^: Ammonium carbamate
AD: Alzheimer's disease
ATRA: All-*trans* retinoic acid
NiFe_2_O_4_@SiO_2_-propyl@glucosamine: 4-Amino glucose functionalized silica propyl coated nickel ferrite nanoparticle
Ae: Alkali metals earth
Pd(dba)_2_: Bis(dibenzylideneacetone)palladium
PdCl_2_(PPh_3_)_2_: Bis-triphenylphosphine palladium dichloride
B_2_pin_2_: Bis(pinacolato)diboron
IMes: 1,3-Bis(2,4,6-trimethylphenyl)-1,3-dihydro-2-*H*-imidazol-2-ylidene
(2-BrC_6_H_4_)OPPh_2_: (2-Bromophenyl)diphenylphosphine oxide
[pd(dppf)Cl_2_CH_2_Cl_2_]: 1,1′-Bis(diphenylphosphino)ferrocene-palladium(ii)dichloride dichloromethane complex
CrCl_3_: Chromium(iii) chloride
Cy_2_PCl: Chlorodicyclohexylphosphane
CAN: Ceric ammonium nitrate
[Ir(OMe)COD]_2_: (1,5-Cyclooctadiene)(methoxy)iridium(i) dimer
CoCl_2_: Cobalt(ii) chloride
DCM: Dichloromethane
EDB: 1,2-Dibromoethane
DCICA: Dichloroiodoisocyanuric acid
K_2_S_2_O_8_: Dipotassium peroxodisulfate
dmbpy: Dimethylbipyridyl
DCC: Dicyclohexylcarbodiimide
DMAP: 4-(Dimethylamino)pyridine
FtsZ: Filamenting temperature-sensitive mutant Z protein
HTS: High-throughput screening
HFIP: 1,1,1,3,3,3-Hexafluoroisopropanol
LCDs: Liquid crystal displays
LiAlH_4_: Lithium aluminium hydride
MOF: Metal–organic frameworks
2-MTHF: 2-Methyltetrahydrofuran
*m*CPBA: *meta*-Chloroperbenzoic acid
MCRs: Multicomponent reaction
NBS: *N*-Bromosuccinimide
NSAIDs: Non-steroidal anti-inflammatory drug
NMEA: *N*-Methylethanolamine
OLEDs: Organic light-emitting diodes
(COCl)_2_: Oxalyl chloride
OMIMs: Organic polymer of intrinsic microporosity
ODCB: *O*-Dichlorobenzene
Pd/CNS: Palladium-catalyst on porous carbon nanospheres
Na[Pd-NAS]: Palladium grafted on natural asphalt sulfonate
PCBs: Polychlorinated biphenyls
PBBs: Polybrominated biphenyls
PPA: Polyphosphoric acid
phen: 1,10-Phenanthroline
KPF_6_: Potassium hexafluorophosphate
[Cp*RhCl_2_]_2_: Pentamethylcyclopentadienyl rhodium dichloride dimer
PBr_3_: Phosphorus tribromide
SMC: Suzuki–Miyaura coupling reaction
CF_3_SO_2_Na: Sodium trifluoromethanesulfinate
AgSbF_6_: Silver hexafluoroantimonate (V)
Ag_2_O: Silver oxide
SmBr_2_: Samarium dibromide
SET: Single-electron transfer
PPh_3_: Triphenylphosphine
TEA: Triethylamine
(Pd_2_(dba)_3_): Tris(dibenzylideneacetone)dipalladium(0)
SOCl_2_: Thionyl chloride
Ph_3_SnCl: Triphenyltin chloride
tfp: Tris-*o*-furylphosphine
SnCl_4_: Tin(iv)chloride
PBu_3_: Tributyl phosphine
CHCl_3_: Trichloromethane
*t*-Boc: *tert*-Butyloxycarbonyl
Pd(PPh_3_)_4_: Tetrakis(triphenylphosphine)palladium
TMEDA: Tetra methyl ethylenediamine
TFAA: Trifluoroacetic anhydride
Bu_4_NBF_4_: Tetrabutylammonium tetrafluoroborate
TEMPO: 2,2,6,6-Tetramethylpiperidine-1-oxyl
TBABr: Tetrabutylammonium bromide
TMSCN: Trimethylsilyl cyanide
Tf_2_O: Trifluoromethanesulfonic anhydride
TBN: *tert*-Butyl nitrite
TBTC: Tributyltin chloride

## Supplementary Material
